# Supplementary material for: Recent advances on the estimation of the thermal reaction norm for sex ratios
Source: PeerJ. 2020 Mar 4;8:e8451. doi: 10.7717/peerj.8451 (PMC7060753; doi:10.7717/peerj.8451)
Supplement: Supplemental Information 2 [file peerj-08-8451-s002.docx]

# Supplementary 1 – Description of the new *flexit* model.

## From logistic to flexit model

The lack of an ideal sigmoid model to describe TSD patterns (i.e., asymmetrical in the transitions toward lower and upper asymptotes) prompted us to develop a new, more versatile sigmoid function, based on the logistic law: $f\left( x \right)=\left( 1+e^{4 S \left( P-x \right)} \right)^{-1}$

This formula has the advantage that $f\left( x=P \right)=0.5$ and $f^{'}\left( x=P \right)=S$. Thus, the temperature $P$ is the temperature at which 50% of the embryos are males or females. However, this model assumes a symmetric transition around $P$. The *A-logistic* model is an asymmetric sigmoid model, with parameter $K$ being a parameter controlling the asymmetry (Godfrey et al. 2003):

$$f\left( x \right)=\left( 1+\left( 2^{K}-1 \right)e^{4 S \left( P-x \right)} \right)^{{-1}/K}$$

As for a logistic model, $f\left( x=P \right)=0.5$. When $K<1$, the transitions from $P$ to the asymptotes showed more acute angles whereas when $K>1$, the transitions from $P$ to asymptotes showed more obtuse angles, as compared to logistic model on both sides of $P$. Hulin et al. (2009) observed that the *A-logistic* model requires that both transitions are either acute, or obtuse and that it was not possible to mix both conditions on each side of $P$. We propose here a new approach to alleviate this constraint.

The first-order derivative of the *A-logistic* model is:

$$f^{'}\left( x \right)=S \frac{4}{K} \left( 2^{K}-1 \right) e^{4 S \left( P-x \right)}\left( 1+\left( 2^{K}-1 \right)e^{4 S \left( P-x \right)} \right)^{-\frac{1}{K}-1}$$

With $f^{'}\left( x=P \right)=S\frac{4}{K}{\left( 2^{K}-1 \right)\left( 2^{-K} \right)}^{\frac{1}{K}+1}$

It follows that slope at $x=P$ depends on both $S$ and $K$. As expected, when $K=1$, $f^{'}\left( x=P \right)=S$.

Then different transitions toward the asymptotes below and above $P$ can be defined with ${K=K}_{1}$ for $x<P$ and ${K=K}_{2}$ for $x\geq P$.

A smooth transition at $x=P$ requires the same $f^{'}\left( x=P \right)$, regardless of the values of $K_{1}$ and $K_{2}$. Then, we search for $S_{1}$ and $S_{2}$ values (respectively for $x<P$ and $x\geq P$) that ensure that $f^{'}\left( x=P \right)$ is equal according to $K_{1}$ and $K_{2}$. It follows that:

$S_{1}=f^{'}\left( x=P \right)\frac{\left( 2^{-K_{1}} \right)^{{-1}/{K_{1}}-1} K_{1}}{4 \left( 2^{K_{1}}-1 \right)}$ and $S_{2}=f^{'}\left( x=P \right)\frac{\left( 2^{-K_{2}} \right)^{{-1}/{K_{2}}-1} K_{2}}{4 \left( 2^{K_{2}}-1 \right)}$

Being symmetric, a logistic law can be written in two ways:

$$f\left( x \right)=\left( 1+e^{4 S \left( P-x \right)} \right)^{-1}=1-\left( 1+e^{4 S \left( x-P \right)} \right)^{-1}$$

which does not apply for the A-logistic model $f\left( x \right)=\left( 1+\left( 2^{K}-1 \right)e^{4 S \left( P-x \right)} \right)^{{-1}/K}$ as

$$\left( 1+\left( 2^{K}-1 \right)e^{4 S \left( P-x \right)} \right)^{{-1}/K}\neq1-\left( 1+\left( 2^{K}-1 \right)e^{4 S \left( x-P \right)} \right)^{{-1}/K}$$

However, both of these forms are interesting as the influence of $K$ on the acute or obtuse transitions toward the asymptotes is reversed. When $K>1$, the transition toward the asymptote is acute when $x<P$ and obtuse when $x>P$ for the form $\left( 1+\left( 2^{K}-1 \right)e^{4 S \left( P-x \right)} \right)^{{-1}/K}$. However, it becomes acute when $x>P$ and obtuse when $x<P$ for the form $1-\left( 1+\left( 2^{K}-1 \right)e^{4 S \left( x-P \right)} \right)^{{-1}/K}$.

This property was used to define the flexible-logistic model or *flexit* model:

$$\left\{ \begin{matrix} x<P & S_{1}=\frac{2^{K_{1}-1} S K_{1}}{2^{K_{1}}-1} & f\left( x \right)=\left( 1+\left( 2^{K_{1}}-1 \right)e^{4 S_{1} \left( P-x \right)} \right)^{{-1}/{K_{1}}} \\ x\geq P & S_{2}=\frac{2^{K_{2}-1} S K_{2}}{2^{K_{2}}-1} & f\left( x \right)={1-\left( 1+\left( 2^{K_{2}}-1 \right)e^{4 S_{2} \left( x-P \right)} \right)}^{{-1}/{K_{2}}} \end{matrix} \right.$$

It should be noted that $2^{K_{i}}-1$ is always different from 0, and $\lim_{K_{i}\to-\infty} S_{i}=0$, and $\lim_{K_{i}\to+\infty} S_{i}=S_{i}\infty$.

*A flexit* model uses 4 parameters and a *logistic* model is nested within it. When $K_{1}=K_{2}=1$, the *flexit* model is a *logistic* model with 2 parameters. The model is not defined for $K_{1}=0$ or $K_{2}=0$. If such a situation occurs, $K_{x}$ is replaced by ${10}^{-9}.$

The *flexit* model is included as a function in the ***HelpersMG*** R package (version 3.7 and higher) (Girondot 2019b) and is included in the tsd() function of the ***embryogrowth*** R package (version 7.5 and higher) (Girondot 2019a).

## Transitional range of temperature (*TRT*) of a *flexit* model of TSD pattern

*TRT l%* is defined as *TRT_H_*- *TRT_L_* with *TRT_H_* being the temperature at which *l* sex ratio is obtained and *TRT_L_* being the temperature at which 1-*l* sex ratio is obtained according to the definition of Girondot (1999).

When $x<P then$ $f\left( x \right)=\left( 1+\left( 2^{K_{1}}-1 \right)e^{4 S_{1} \left( P-x \right)} \right)^{{-1}/{K_{1}}}$ with $S_{1}=\frac{2^{K_{1}-1} S K_{1}}{2^{K_{1}}-1}$

$$\frac{1}{\left( 1+\left( 2^{K_{1}}-1 \right)e^{4 S_{1} \left( P-{TRT}_{L} \right)} \right)^{1/{K_{1}}}}=1-l$$

$$\left( 1+\left( 2^{K_{1}}-1 \right)e^{4 S_{1} \left( P-{TRT}_{L} \right)} \right)^{1/{K_{1}}}=1/\left( 1-l \right)$$

$$1+\left( 2^{K_{1}}-1 \right)e^{4 S_{1} \left( P-{TRT}_{L} \right)}=\left( 1/\left( 1-l \right) \right)^{K_{1}}$$

$$\left( 2^{K_{1}}-1 \right)e^{4 S_{1} \left( P-{TRT}_{L} \right)}=\left( 1/\left( 1-l \right) \right)^{K_{1}}-1$$

$$e^{4 S_{1} \left( P-{TRT}_{L} \right)}=\frac{\left( 1/\left( 1-l \right) \right)^{K_{1}}-1}{2^{K_{1}}-1}$$

$$4 S_{1} \left( P-{TRT}_{L} \right)=ln\frac{\left( 1/\left( 1-l \right) \right)^{K_{1}}-1}{2^{K_{1}}-1}$$

$$P-{TRT}_{L}=\frac{1}{4 S_{1}}ln\frac{\left( 1/\left( 1-l \right) \right)^{K_{1}}-1}{2^{K_{1}}-1}$$

$${TRT}_{L}=P-\frac{1}{4 S_{1}}ln\frac{\left( 1/\left( 1-l \right) \right)^{K_{1}}-1}{2^{K_{1}}-1}$$

When $x\geq P then$ $f\left( x \right)={1-\left( 1+\left( 2^{K_{2}}-1 \right)e^{4 S_{2} \left( {TRT}_{H}-P \right)} \right)}^{{-1}/{K_{2}}}$ with $S_{2}=\frac{2^{K_{2}-1} S K_{2}}{2^{K_{2}}-1}$

$$1-\frac{1}{\left( 1+\left( 2^{K_{2}}-1 \right)e^{4 S_{2} \left( {TRT}_{H}-P \right)} \right)^{1/{K_{2}}}}=l$$

$$\frac{1}{\left( 1+\left( 2^{K_{2}}-1 \right)e^{4 S_{2} \left( {TRT}_{H}-P \right)} \right)^{1/{K_{2}}}}=\left( 1-l \right)$$

$$\left( 1+\left( 2^{K_{2}}-1 \right)e^{4 S_{2} \left( {TRT}_{H}-P \right)} \right)^{1/{K_{2}}}=1/\left( 1-l \right)$$

$$1+\left( 2^{K_{2}}-1 \right)e^{4 S_{2} \left( {TRT}_{H}-P \right)}=\left( 1/\left( 1-l \right) \right)^{K_{2}}$$

$$\left( 2^{K_{2}}-1 \right)e^{4 S_{2} \left( {TRT}_{H}-P \right)}=\left( 1/\left( 1-l \right) \right)^{K_{2}}-1$$

$$e^{4 S_{2} \left( {TRT}_{H}-P \right)}=\frac{\left( 1/\left( 1-l \right) \right)^{K_{2}}-1}{2^{K_{2}}-1}$$

$$4 S_{2} \left( {TRT}_{H}-P \right)=ln\frac{\left( 1/\left( 1-l \right) \right)^{K_{2}}-1}{2^{K_{2}}-1}$$

$${TRT}_{H}-P=\frac{1}{4 S_{2}}ln\frac{\left( 1/\left( 1-l \right) \right)^{K_{2}}-1}{2^{K_{2}}-1}$$

$${TRT}_{H}=P+\frac{1}{4 S_{2}}ln\frac{\left( 1/\left( 1-l \right) \right)^{K_{2}}-1}{2^{K_{2}}-1}$$

It follows that ${TRT=TRT}_{H}-{TRT}_{L}$

$$TRT=P+\frac{1}{4 S_{2}}ln\frac{\left( 1/\left( 1-l \right) \right)^{K_{2}}-1}{2^{K_{2}}-1}-P+\frac{1}{4 S_{1}}ln\frac{\left( 1/\left( 1-l \right) \right)^{K_{1}}-1}{2^{K_{1}}-1}$$

$$TRT=\frac{1}{4 S_{2}}ln\frac{\left( 1/\left( 1-l \right) \right)^{K_{2}}-1}{2^{K_{2}}-1}+\frac{1}{4 S_{1}}ln\frac{\left( 1/\left( 1-l \right) \right)^{K_{1}}-1}{2^{K_{1}}-1}$$
