# Supplementary material for: Recent advances on the estimation of the thermal reaction norm for sex ratios
Source: PeerJ. 2020 Mar 4;8:e8451. doi: 10.7717/peerj.8451 (PMC7060753; doi:10.7717/peerj.8451)
Supplement: Supplemental Information 3 [file peerj-08-8451-s003.docx]

Recent advances on the estimation of the thermal reaction norm for sex ratios in sea turtle nest incubations using olive ridley data as an example

F. Alberto Abreu-Grobois, B. Alejandra Morales-Mérida, Catherine Hart, Jean-Michel Guillon, Matthew H. Godfrey, Erik Navarro, Marc Girondot

21 novembre 2019

**Worked out examples of the publication data using package *embryogrowth***

As a support to the publication, we use the data for *Lepidochelys olivacea* mentioned to illustrate the latest methods for the estimation of sex ratios from egg incubations at constant temperatures.

Interest on the thermal reaction norm for sex ratio at constant incubation temperatures for conservation is not trivial as the variation in TSD patterns can have profound consequence for conservation:

Carter, A.L., Bodensteiner, B.L., Iverson, J.B., Milne‐Zelman, C.L., Mitchell, T.S., Refsnider, J.M., Warner, D.A., Janzen, F.J., White, C., 2019. Breadth of the thermal response captures individual and geographic variation in temperature‐dependent sex determination. Functional Ecology.

Hulin, V., Delmas, V., Girondot, M., Godfrey, M.H., Guillon, J.-M., 2009. Temperature-dependent sex determination and global change: Are some species at greater risk? Oecologia 160, 493-506.

*Note: this analysis incorporates new data from Mexico not included in the package distribution at the time of publication.*

Version >7.6 of embryogrowth package must be installed, or from CRAN, or from <http://max2.ese.u-psud.fr/epc/conservation/CRAN/> site.

ToInstall <- FALSE
if (!("embryogrowth" %in% installed.packages())) {
 ToInstall <- TRUE
} else {
 x <- packageVersion("embryogrowth")
 if (x < "7.6") {
 ToInstall <- TRUE
 }
}
}

if (ToInstall) {
 install.packages("http:///max2.ese.u-psud.fr/epc/conservation/CRAN/HelpersMG.tar.gz", repos=NULL, type="source")
 install.packages("http:///max2.ese.u-psud.fr/epc/conservation/CRAN/embryogrowth.tar.gz", repos=NULL, type="source")
}

library("embryogrowth")

## Loading required package: deSolve

## Loading required package: optimx

## Loading required package: numDeriv

## Loading required package: parallel

## Loading required package: HelpersMG

## Loading required package: lme4

## Loading required package: Matrix

## Loading required package: coda

library("openxlsx")
library("maps")
library("mapdata")
library("sp")
library("car")

## Loading required package: carData

## Registered S3 methods overwritten by 'car':
## method from
## influence.merMod lme4
## cooks.distance.influence.merMod lme4
## dfbeta.influence.merMod lme4
## dfbetas.influence.merMod lme4

##
## Attaching package: 'car'

## The following objects are masked from 'package:HelpersMG':
##
## ellipse, logit

DatabaseTSD$Version[1]

## [1] "2019-11-19"

packageVersion("embryogrowth")

## [1] '7.6.7'

db <- subset(DatabaseTSD, subset=(Species=="Lepidochelys olivacea") & (!is.na(Sexed)) & (Sexed!=0),
 select=c("Area", "RMU", "Incubation.temperature", "Incubation.temperature.SD",
 "Incubation.temperature.Amplitude", "2ndThird.Incubation.temperature.Amplitude",
 "Sexed", "Males", "Females", "Intersexes",
 "Reference"))

# write.xlsx(db, file="Table1.xlsx")

unique(as.character(subset(DatabaseTSD, Species=="Lepidochelys olivacea" & (!is.na(Sexed) & Sexed!=0) & (is.na(Incubation.temperature.Amplitude) | (Incubation.temperature.Amplitude<1)), select="RMU")[, 1]))

## [1] "Atlantic, West" "Pacific, E" "Indian, NE"

Only data with amplitude of temperatures during incubation lower than 2 °C are used. Indeed, incubating with large temperature variations is known to provoke higher feminization than expected (Georges, 1989).

As a consequence, these data will not be used:

Georges, A., 1989. Female turtles from hot nests: Is it duration of incubation or proportion of development at high temperatures that matters? Oecologia 81, 323-328.

Sandoval Espinoza (2012) indicates the use of data from incubations at different varying temperatures (pages 53-55) but the sex ratio data are not available in the thesis.

Sandoval Espinoza, S., 2012. Proporción sexual en crías de tortuga *Lepidochelys olivacea* en corrales de incubación del Pacífico mexicano, In Centro Interdisciplinario de Ciencias Marinas. p. 158. Instituto Politécnico Nacional, La Paz, BCS, México.

**Data come from:**

Castheloge, V.D., de D. dos Santos, M.R., de Castilhos, J.C., de J. Filho, P.R., de C. Gomes, L., Clemente-Carvalho, R.B.G., Ferreira, P.D., 2018. Pivotal temperature and hatchling sex ratio of olive ridley sea turtles *Lepidochelys olivacea* from the South Atlantic coast of Brazil. Herpetological Conservation and Biology 13, 488-496.

McCoy, C.J., Vogt, R.C., Censky, E.J., 1983. Temperature-controlled sex determination in the sea turtle *Lepidochelys olivacea*. J. Herpetol. 17, 404-406.

Wibbels, T., Rostal, D.C., Byles, R., 1998. High pivotal temperature in the sex determination of the olive ridley sea turtle, *Lepidochelys olivacea*, from Playa Nancite, Costa Rica. Copeia 1998, 1086-1088.

Dimond, M.T., 1985. Some effects of temperature on turtle egg incubation, In Recent Advances in Developmental Biology of Animals. eds S.C. Goel, C.B.L. Srivastava, pp. 35-39. Indian Society of Developmental Biologists, Poona, India.

López Correa, J.Y., 2010. Diferenciación gonádica en crias de *Lepidochelys olivacea* (Eschscholtz, 1829) (Testudinata: Cheloniidae), p. 108. Instituto Politécnico Nacional, Centro Interdisciplinaria de Ciencias Marinas, La Paz, BCS.

Mohanty-Hejmadi, P., Behra, M., Dimond, M.T., 1985. Temperature dependent sex differentiation in the olive ridley *Lepidochelys olivacea* and its implications for conservation, In Symposium on Endangered Marine Animals and Marine Parks. pp. 1-5. Marine Biological Association of India, Cochin.

Merchant-Larios, H., Ruiz-Ramirez, S., Moreno-Mendoza, N., Marmolejo-Valencia, A., 1997. Correlation among thermosensitive period, estradiol response, and gonad differentiation in the sea turtle *Lepidochelys olivacea*. General and Comparative Endocrinology 107, 373-385.

Merchant-Larios, H., Villalpando-Fierro, I., Centeno-Urruiza, B., 1989. Gonadal morphogenesis under controlled temperature in the sea turtle *Lepidochelys olivacea*. Herpetol. Monographs 3, 43-61.

Navarro Sánchez, E.J., 2015. Efecto de la temperatura de incubación y la diferenciación sexual sobre la morfología de crías de tortuga marina *Lepidochelys olivacea*, Ciencias del mar y limnología. Universidad Nacional Autónoma de México, México, D.F., p. 89.

# Sex ratios from field studies

These data cannot be used for TSD pattern because they come from field studies with large temperature variation:

Maulany, R. I., D. T. Booth, and G. S. Baxter. 2012. Emergence Success and Sex Ratio of Natural and Relocated Nests of Olive Ridley Turtles from Alas Purwo National Park, East Java, Indonesia. Copeia :738-747. (season 2009 and 2010)

Hernandez-Echeagaray, O. E., R. Hernandez-Cornejo, M. Harfush-Melendez, and A. Garcia-Gasca. 2012. Evaluation of Sex Ratios of the Olive Ridley Sea Turtle (*Lepidochelys olivacea*) on the Arribada Nesting Beach, La Escobilla, Mexico. Marine Turtle Newsletter 133:12-16. (Season 2009-1010)

Garcia, A., G. Ceballos, and R. Adaya. 2003. Intensive beach management as an improved sea turtle conservation strategy in Mexico. Biological Conservation 111:253-261. (Playa Cuixmala, Jalisco, Mexico, season 1994)

Sandoval Espinoza, S., 2008. Pronóstico de la temperatura de los nidos de tortuga golfina (Lepidochelys olivacea) en función de la temperatura ambiente, la profundidad y el calor metabólico, In Centro Interdisciplinario De Ciencias Marinas. p. 84. Instituto Politécnico Nacional, La Paz, Mexico.

Sandoval Espinoza, S., 2012. Proporción sexual en crías de tortuga Lepidochelys olivacea en corrales de incubación del Pacífico mexicano, In Centro Interdisciplinario de Ciencias Marinas. p. 158. Instituto Politécnico Nacional, La Paz, BCS, México.

# Load data

Lo_PacificE_Mexico <- subset(DatabaseTSD, RMU=="Pacific, E" & Country =="Mexico" &
 Species=="Lepidochelys olivacea" & (!is.na(Sexed) & Sexed!=0), select=c("Males", "Females", "Sexed", "Incubation.temperature", "Reference", "Longitude", "Latitude", "Area"))

Lo_PacificE_CostaRica <- subset(DatabaseTSD, RMU=="Pacific, E" & Country =="Costa Rica" &
 Species=="Lepidochelys olivacea" & (!is.na(Sexed) & Sexed!=0), select=c("Males", "Females", "Sexed", "Incubation.temperature", "Reference", "Longitude", "Latitude", "Area"))

Lo_PacificE <- subset(DatabaseTSD, RMU=="Pacific, E" &
 Species=="Lepidochelys olivacea" & (!is.na(Sexed) & Sexed!=0), select=c("Males", "Females", "Sexed", "Incubation.temperature", "Reference", "Longitude", "Latitude", "Area"))

Lo_AtlanticWest <- subset(DatabaseTSD, RMU=="Atlantic, West" &
 Species=="Lepidochelys olivacea" & (!is.na(Sexed) & Sexed!=0), select=c("Males", "Females", "Sexed", "Incubation.temperature", "Reference", "Longitude", "Latitude", "Area"))
Lo_IndianNE <- subset(DatabaseTSD, RMU=="Indian, NE" &
 Species=="Lepidochelys olivacea" & (!is.na(Sexed) & Sexed!=0), select=c("Males", "Females", "Sexed", "Incubation.temperature"))
Lo_Global <- subset(DatabaseTSD, Species=="Lepidochelys olivacea" & (!is.na(Sexed) & Sexed!=0), select=c("Males", "Females", "Intersexes", "Sexed", "Incubation.temperature", "Reference", "Longitude", "Latitude", "Area"))

The total number of sexed embryos is 464 with 277 males, 168 females and 19 intersexes. The total number of incubation temperatures is 22.

For Costa Rica, the total number of sexed embryos is 174 with 119 males, 55 females and 0 intersexes. The total number of incubation temperatures is 8.

For Mexico, the total number of sexed embryos is 190 with 120 males, 70 females and 0 intersexes. The total number of incubation temperatures is 14.

At the scale of East Pacific RMU, the total number of sexed embryos is 364 with 239 males, 125 females and 0 intersexes. The total number of incubation temperatures is 22.

From India, the total number of sexed embryos is 31 with 13 males, 18 females and 0 intersexes. The total number of incubation temperatures is 6.

From Brazil, the total number of sexed embryos is 50 with 25 males, 25 females and 0 intersexes. The total number of incubation temperatures is 12.

# Map with locations

# pdf(file = "Figure 1.pdf", width = 7, height = 4, pointsize = 12)

par(mar=c(5, 5, 1, 1))
map('world', fill=TRUE, col="lightgrey", border=NA)


points(unique(Lo_Global$Longitude), unique(Lo_Global$Latitude), col="white", pch=16, cex=3)
points(unique(Lo_Global$Longitude), unique(Lo_Global$Latitude), col="black", pch=1, cex=3)

# text(unique(Lo_Global$Longitude), unique(Lo_Global$Latitude), unique(Lo_Global$Area))

tl <- c(1, 2, 3, 4, "", "")
tp <- c(1, 3, 2, 4, 5, 6)
text(unique(Lo_Global$Longitude)[tp], unique(Lo_Global$Latitude)[tp], labels = tl)

segments(x0=unique(Lo_Global$Longitude)[5], y0=unique(Lo_Global$Latitude)[5],
 x1=unique(Lo_Global$Longitude)[5]-20, y1=unique(Lo_Global$Latitude)[5]-3)

segments(x0=unique(Lo_Global$Longitude)[6], y0=unique(Lo_Global$Latitude)[6],
 x1=unique(Lo_Global$Longitude)[5]-20, y1=unique(Lo_Global$Latitude)[6]+3)

text(unique(Lo_Global$Longitude)[5]-25, unique(Lo_Global$Latitude)[5]-4, labels = "5")
text(unique(Lo_Global$Longitude)[5]-25, unique(Lo_Global$Latitude)[5]+5, labels = "6")

degAxis(side=1, cex.axis=1) # in package sp
par(xpd=TRUE)
degAxis(side= 2, las=1, cex.axis=1, pos=-170, at=seq(from=-90, to=90, length.out = 5))


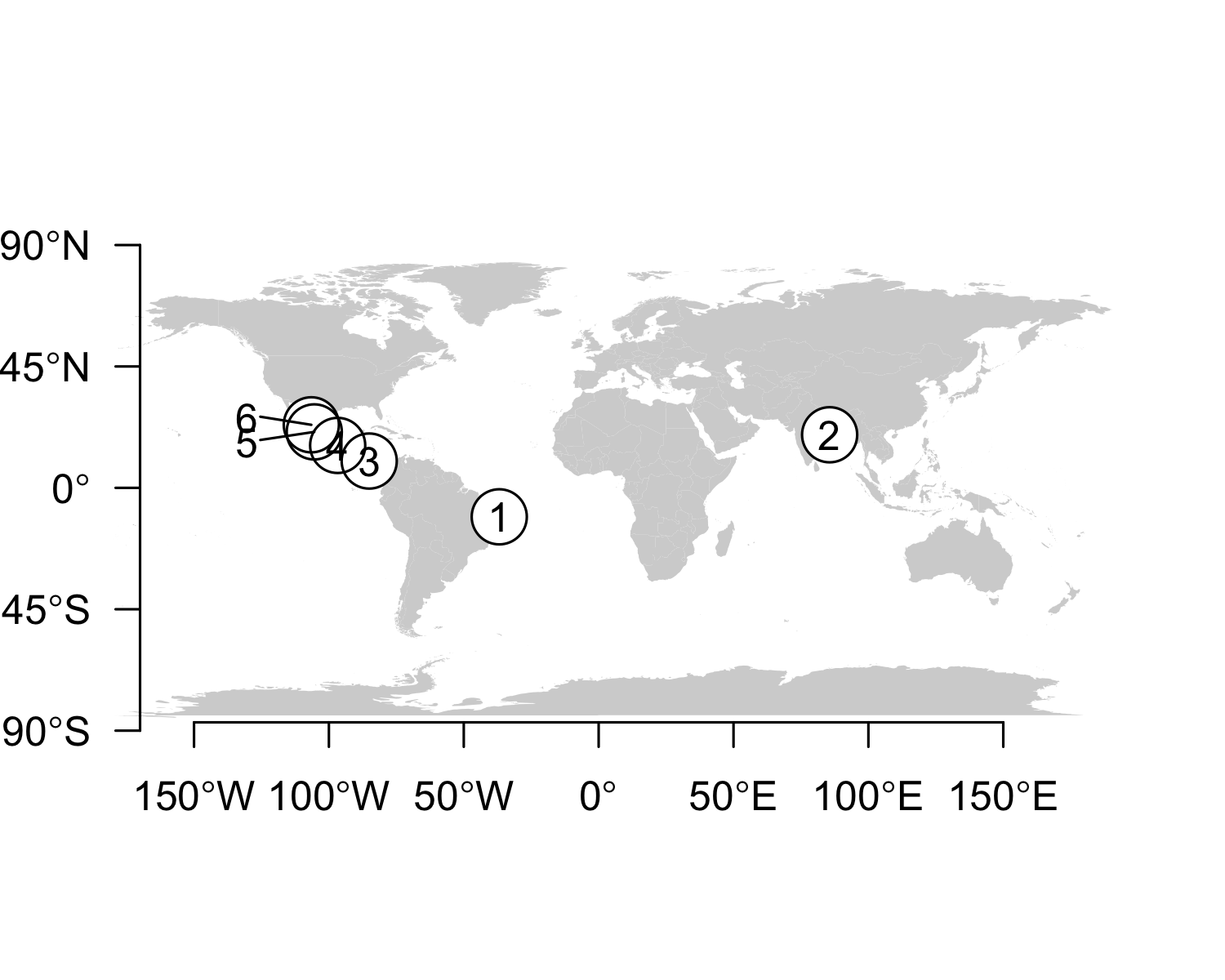


# dev.off()

# Fit TSD model using maximum likelihood with all data

The flexit model is selected but standard error of some parameters is very high. We will continue with logistic model.

Hill and A-logistic model are constrained asymetrical model. If asymetry is tested, it is better to use flexit model.

The choice of the model can have profound implication for the estimate of PT and TRT.

It will be used as a prior for Bayesian analysis

tsdL_Lo_Global_logistic <- with (Lo_Global, tsd(males=Males, females=Females,
 temperatures=Incubation.temperature,
 equation="logistic"))

## Loading required namespace: lmf

## [1] "The pivotal temperature is 30.385 CI95% 30.211;30.558"
## [1] "The transitional range of temperatures is 2.418 CI95% 1.844;2.991"
## [1] "The lower limit of transitional range of temperatures is 29.177 CI95% 28.870;29.479"
## [1] "The higher limit of transitional range of temperatures is 31.594 CI95% 31.232;31.946"

tsdL_Lo_Global_logistic$deviance

## [1] 45.68434

tsdL_Lo_Global_logistic$df

## [1] 38

tsdL_Lo_Global_logistic$pvalue

## [1] 0.1831169

par(mar=c(4, 4, 1, 1)+0.4)
par(xpd=FALSE)
hist(tsdL_Lo_Global_logistic$NullDeviance, main="", las=1,
 xlab="Deviance for null distribution", ylim=c(0, 250))
segments(x0=tsdL_Lo_Global_logistic$deviance, x1=tsdL_Lo_Global_logistic$deviance,
 y0=0, y1=ScalePreviousPlot()$ylim["end"], lty=2, lwd=2)
text(x=tsdL_Lo_Global_logistic$deviance-1, y=ScalePreviousPlot()$ylim["end"]*0.95,
 labels=round(1-tsdL_Lo_Global_logistic$NullDeviancePvalue, digits = 2), pos=2)
text(x=tsdL_Lo_Global_logistic$deviance+1, y=ScalePreviousPlot()$ylim["end"]*0.95,
 labels=round(tsdL_Lo_Global_logistic$NullDeviancePvalue, digits = 2), pos=4)


text(x=ScalePreviousPlot()$xlim["begin"]+ScalePreviousPlot()$xlim["range"]*0.70, y=210, labels = expression(bold("Logistic")), pos=4, cex=1.1)
text(x=ScalePreviousPlot()$xlim["begin"]+ScalePreviousPlot()$xlim["range"]*0.70, y=180, labels = paste("Deviance=", round(x = tsdL_Lo_Global_logistic$deviance, digits = 2)), pos=4)
text(x=ScalePreviousPlot()$xlim["begin"]+ScalePreviousPlot()$xlim["range"]*0.70, y=160, labels = paste("df=", round(x = tsdL_Lo_Global_logistic$df, digits = 0)), pos=4)
text(x=ScalePreviousPlot()$xlim["begin"]+ScalePreviousPlot()$xlim["range"]*0.70, y=140, labels = paste("p-value=", round(x = tsdL_Lo_Global_logistic$pvalue, digits = 2)), pos=4)

par(xpd=TRUE)
text(x=tsdL_Lo_Global_logistic$deviance, y=ScalePreviousPlot()$ylim["end"]*1.1, labels ="Observed deviance")


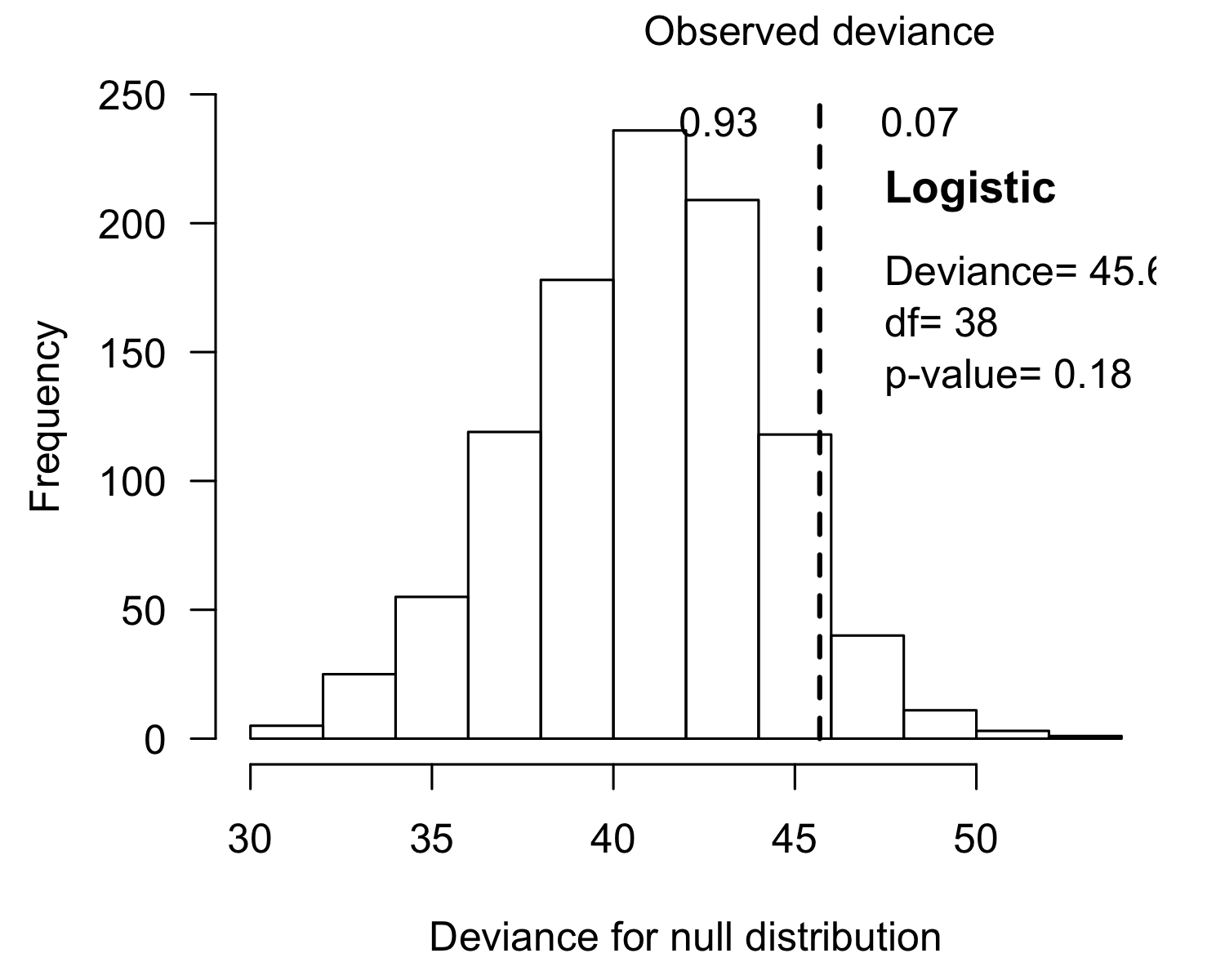


tsdL_Lo_Global_Hill <- with (Lo_Global, tsd(males=Males, females=Females,
 temperatures=Incubation.temperature,
 equation="Hill", replicate.NullDeviance = 0))

## [1] "The pivotal temperature is 30.377 CI95% 30.205;30.549"
## [1] "The transitional range of temperatures is 2.420 CI95% 1.860;2.983"
## [1] "The lower limit of transitional range of temperatures is 29.188 CI95% 28.906;29.481"
## [1] "The higher limit of transitional range of temperatures is 31.613 CI95% 31.252;31.978"

tsdL_Lo_Global_Alogistic <- with (Lo_Global, tsd(males=Males, females=Females,
 temperatures=Incubation.temperature,
 equation="A-logistic",
 parameters.initial = c(tsdL_Lo_Global_logistic$par, K = -3), replicate.NullDeviance = 0))

## SE for K too high in 6479 cases out of 16479

## Warning in P_TRT(x = result, l = l, replicate.CI = replicate.CI, probs = c((1
## - : Use results with caution; it is probably better to use MCMC

## [1] "The pivotal temperature is 30.528 CI95% 30.327;30.741"
## [1] "The transitional range of temperatures is 2.308 CI95% -2.125;3.182"
## [1] "The lower limit of transitional range of temperatures is 29.057 CI95% 28.544;31.106"
## [1] "The higher limit of transitional range of temperatures is 31.343 CI95% 28.861;31.853"

tsdL_Lo_Global_flexit <- with (Lo_Global, tsd(males=Males, females=Females,
 temperatures=Incubation.temperature,
 equation="flexit",
 parameters.initial = c(tsdL_Lo_Global_logistic$par, K1 = -3, K2 = 200)))

## SE for S too high in 72 cases out of 19425

## SE for K1 too high in 776 cases out of 19425

## SE for K2 too high in 8577 cases out of 19425

## Warning in P_TRT(x = result, l = l, replicate.CI = replicate.CI, probs = c((1
## - : Use results with caution; it is probably better to use MCMC

## [1] "The pivotal temperature is 30.563 CI95% 30.361;30.774"
## [1] "The transitional range of temperatures is 2.092 CI95% 1.386;3.619"
## [1] "The lower limit of transitional range of temperatures is 28.636 CI95% 27.219;29.193"
## [1] "The higher limit of transitional range of temperatures is 30.660 CI95% 30.417;31.186"

tsdL_Lo_Global_flexit$deviance

## [1] 33.94546

tsdL_Lo_Global_flexit$df

## [1] 36

tsdL_Lo_Global_flexit$pvalue

## [1] 0.5666485

par(mar=c(4, 4, 1, 1)+0.4)
par(xpd=FALSE)
hist(tsdL_Lo_Global_flexit$NullDeviance, main="", las=1,
 xlab="Deviance for null distribution", ylim=c(0, 250), xlim=c(20, 45))
segments(x0=tsdL_Lo_Global_flexit$deviance, x1=tsdL_Lo_Global_flexit$deviance,
 y0=0, y1=ScalePreviousPlot()$ylim["end"], lty=2, lwd=2)
text(x=tsdL_Lo_Global_flexit$deviance-1, y=ScalePreviousPlot()$ylim["end"]*0.95,
 labels=round(1-tsdL_Lo_Global_flexit$NullDeviancePvalue, digits = 2), pos=2)
text(x=tsdL_Lo_Global_flexit$deviance+5, y=ScalePreviousPlot()$ylim["end"]*0.95,
 labels=round(tsdL_Lo_Global_flexit$NullDeviancePvalue, digits = 2), pos=4)


text(x=ScalePreviousPlot()$xlim["begin"]+ScalePreviousPlot()$xlim["range"]*0.70, y=210, labels = expression(bold("Flexit")), pos=4, cex=1.1)
text(x=ScalePreviousPlot()$xlim["begin"]+ScalePreviousPlot()$xlim["range"]*0.70, y=180, labels = paste("Deviance=", round(x = tsdL_Lo_Global_flexit$deviance, digits = 2)), pos=4)
text(x=ScalePreviousPlot()$xlim["begin"]+ScalePreviousPlot()$xlim["range"]*0.70, y=160, labels = paste("df=", round(x = tsdL_Lo_Global_flexit$df, digits = 0)), pos=4)
text(x=ScalePreviousPlot()$xlim["begin"]+ScalePreviousPlot()$xlim["range"]*0.70, y=140, labels = paste("p-value=", round(x = tsdL_Lo_Global_flexit$pvalue, digits = 2)), pos=4)

par(xpd=TRUE)
text(x=tsdL_Lo_Global_flexit$deviance, y=ScalePreviousPlot()$ylim["end"]*1.1, labels ="Observed deviance")


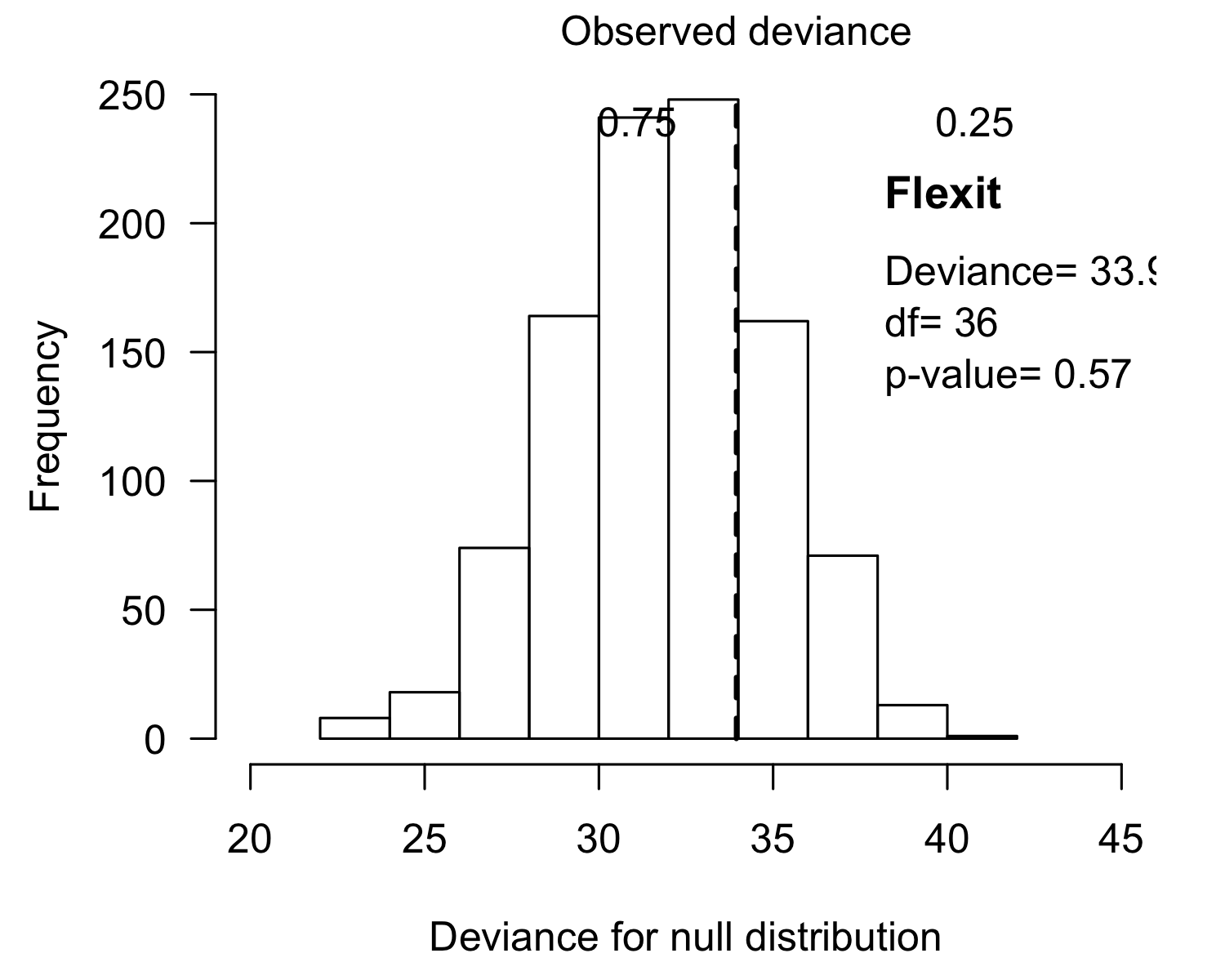


par(xpd=FALSE)


(comp0 <- compare_AICc(logistic=tsdL_Lo_Global_logistic,
 # Hill=tsdL_Lo_Global_Hill,
 # Alogistic=tsdL_Lo_Global_Alogistic,
 flexit=tsdL_Lo_Global_flexit))

## [1] "The lowest AICc (62.596) is for series flexit with Akaike weight=0.970"

## AICc DeltaAICc Akaike_weight
## logistic 69.51621 6.920342 0.03046698
## flexit 62.59586 0.000000 0.96953302

result <- tsdL_Lo_Global_logistic
out1 <- data.frame(model="Logistic",
 P=paste(round(result$par["P"], digits = 2), "SE", round(result$SE["P"], digits = 2)),
 S=paste(round(result$par["S"], digits = 2), "SE", round(result$SE["S"], digits = 2)),
 K1="",
 K2="",
 LnL=as.character(round(result$value, digits = 2)),
 AICc=as.character(round(result$AICc, digits = 2)),
 "deltaAICc"=as.character(round(comp0[1, "DeltaAICc"], digits = 2)),
 Akaike.Weight=as.character(round(comp0[1, "Akaike_weight"], digits = 2)),
 Deviance=as.character(round(result$deviance, digits = 2)),
 df=as.character(round(result$df, digits = 0)),
 p.value=as.character(round(result$pvalue, digits = 6)),
 p.Deviance.Null.model=as.character(round(result$NullDeviancePvalue, digits = 2))
)

result <- tsdL_Lo_Global_flexit
out1 <- rbind(out1, data.frame(model="Flexit",
 P=paste(round(result$par["P"], digits = 2), "SE", round(result$SE["P"], digits = 2)),
 S=paste(round(result$par["S"], digits = 2), "SE", round(result$SE["S"], digits = 2)),
 K1=paste(round(result$par["K1"], digits = 2), "SE", round(result$SE["K1"], digits = 2)),
 K2=paste(round(result$par["K2"], digits = 2), "SE", round(result$SE["K2"], digits = 2)),
 LnL=as.character(round(result$value, digits = 2)),
 AICc=as.character(round(result$AICc, digits = 2)),
 "deltaAICc"=as.character(round(comp0[2, "DeltaAICc"], digits = 2)),
 Akaike.Weight=as.character(round(comp0[2, "Akaike_weight"], digits = 2)),
 Deviance=as.character(round(result$deviance, digits = 2)),
 df=as.character(round(result$df, digits = 0)),
 p.value=as.character(round(result$pvalue, digits = 6)),
 p.Deviance.Null.model=as.character(round(result$NullDeviancePvalue, digits = 2))
)
)

# write.xlsx(x=out1, file="Table2.xlsx", asTable = FALSE)

plot(tsdL_Lo_Global_logistic)
text(x=25, y=0.1, labels = "Logistic model", pos=4)


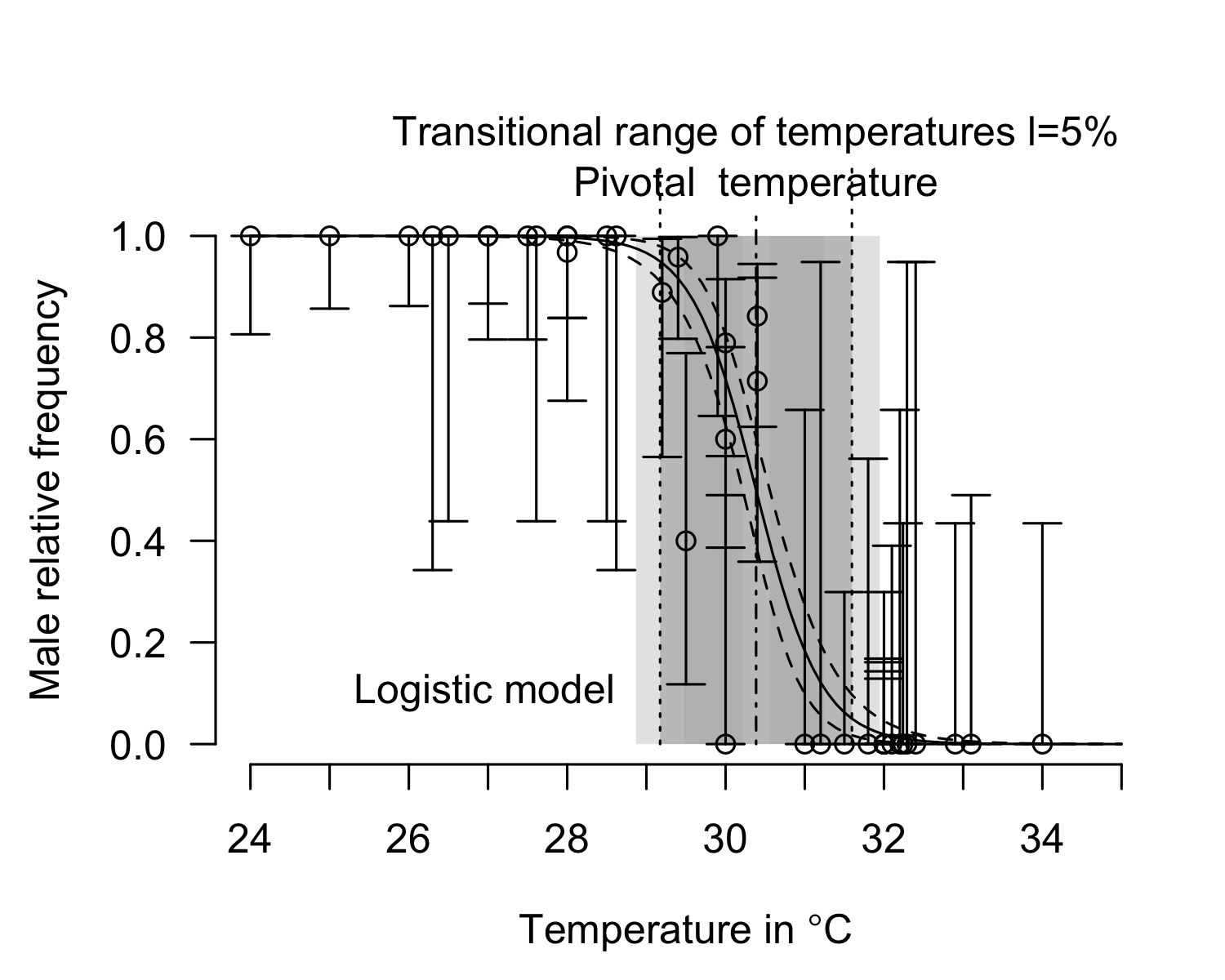


P_TRT(x=tsdL_Lo_Global_logistic, replicate.CI = 10000)$P_TRT_quantiles

## lower.limit.TRT higher.limit.TRT TRT PT
## 2.5% 28.88051 31.23897 1.859326 30.21505
## 50% 29.17592 31.59268 2.415162 30.38477
## 97.5% 29.47869 31.93918 2.960756 30.55537

plot(tsdL_Lo_Global_Hill)
text(x=25, y=0.1, labels = "Hill model", pos=4)


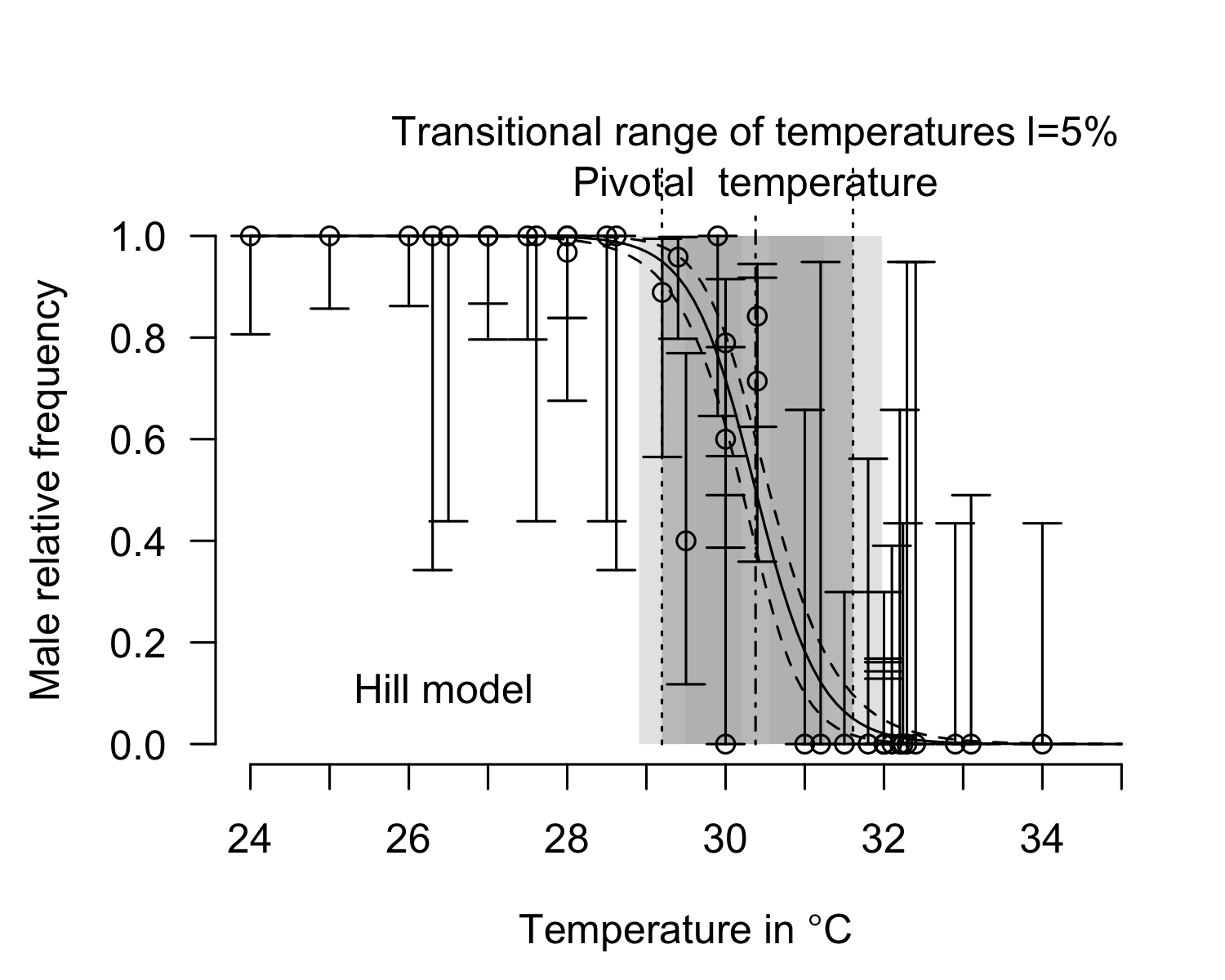


P_TRT(x=tsdL_Lo_Global_Hill, replicate.CI = 10000)$P_TRT_quantiles

## lower.limit.TRT higher.limit.TRT TRT PT
## 2.5% 28.90209 31.24573 1.865656 30.20252
## 50% 29.18872 31.61221 2.420177 30.37532
## 97.5% 29.47524 31.97374 2.973905 30.54548

plot(tsdL_Lo_Global_Alogistic)

## SE for K too high in 6520 cases out of 16520

## Warning in P_TRT(x = x, resultmcmc = resultmcmc, chain = chain, l = l,
## replicate.CI = replicate.CI, : Use results with caution; it is probably better
## to use MCMC

text(x=25, y=0.1, labels = "A-logistic model", pos=4)


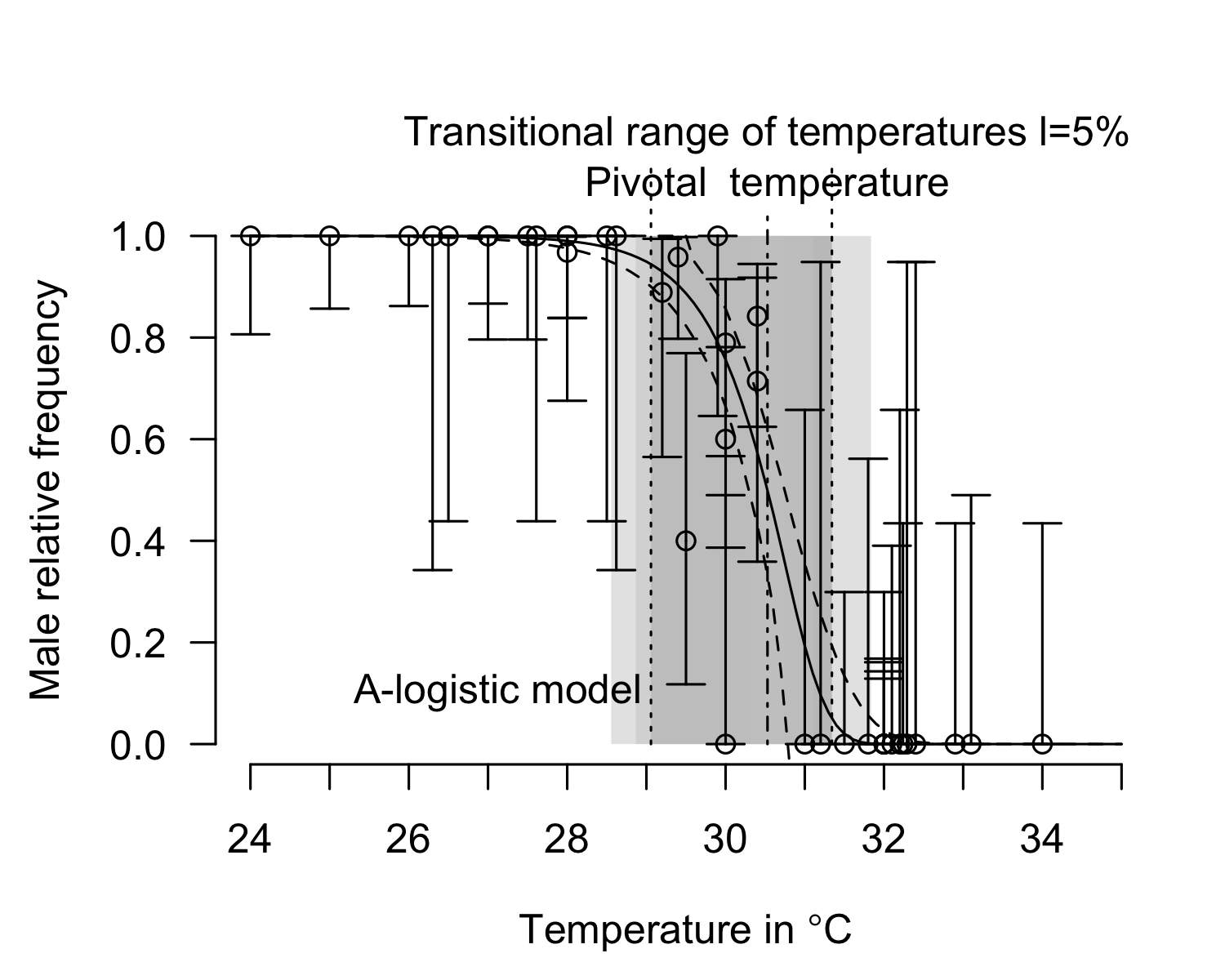


P_TRT(x=tsdL_Lo_Global_Alogistic, replicate.CI = 10000)$P_TRT_quantiles

## SE for K too high in 6478 cases out of 16478

## Warning in P_TRT(x = tsdL_Lo_Global_Alogistic, replicate.CI = 10000): Use
## results with caution; it is probably better to use MCMC

## lower.limit.TRT higher.limit.TRT TRT PT
## 2.5% 28.54566 28.86154 -2.127267 30.32220
## 50% 29.05583 31.34341 2.307115 30.52891
## 97.5% 31.10574 31.84464 3.185474 30.73141

plot(tsdL_Lo_Global_flexit)

## SE for S too high in 70 cases out of 19119

## SE for K1 too high in 747 cases out of 19119

## SE for K2 too high in 8302 cases out of 19119

## Warning in P_TRT(x = x, resultmcmc = resultmcmc, chain = chain, l = l,
## replicate.CI = replicate.CI, : Use results with caution; it is probably better
## to use MCMC

text(x=25, y=0.1, labels = "Flexit model", pos=4)


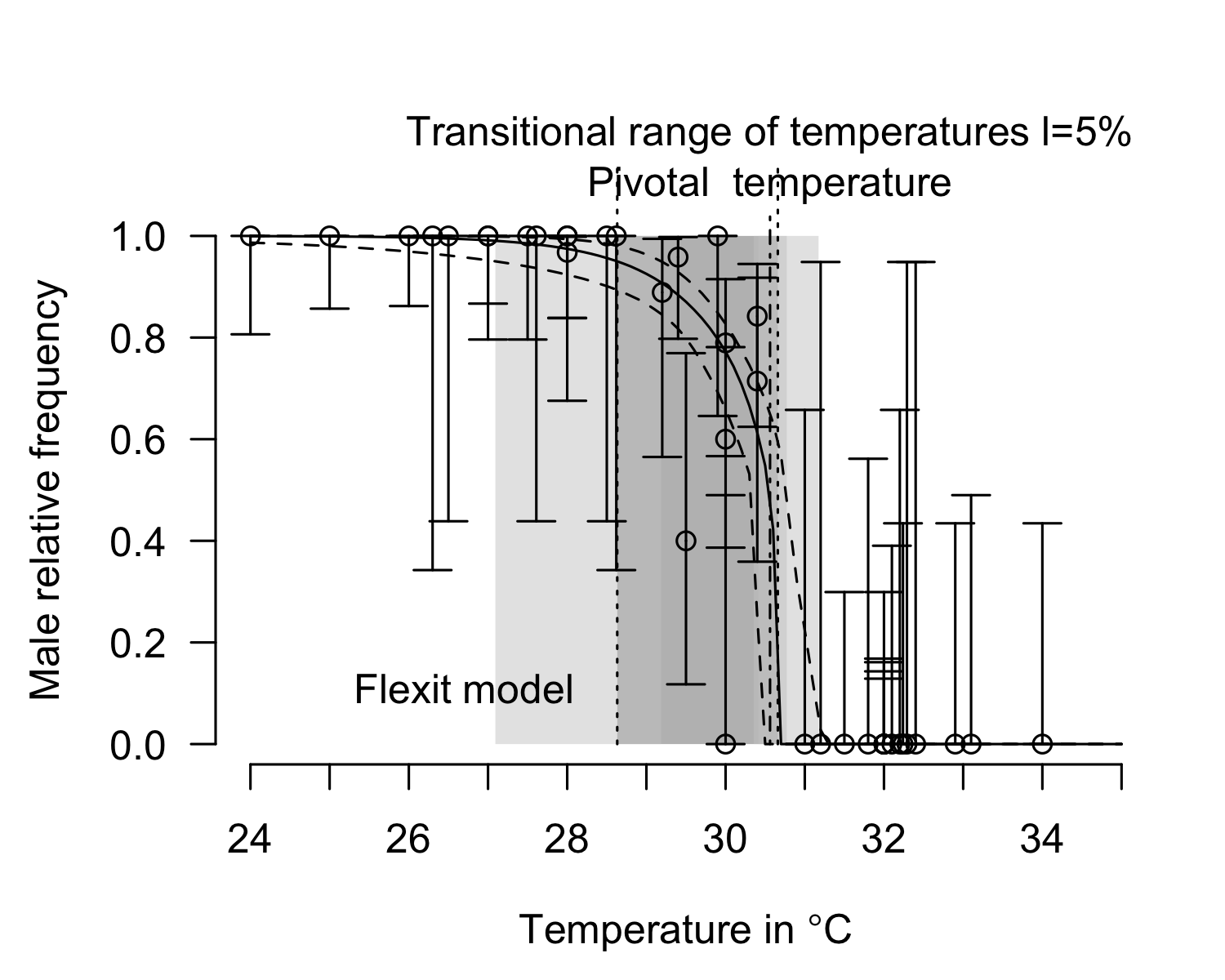


P_TRT(x=tsdL_Lo_Global_flexit, replicate.CI = 10000)$P_TRT_quantiles

## SE for S too high in 77 cases out of 19162

## SE for K1 too high in 678 cases out of 19162

## SE for K2 too high in 8407 cases out of 19162

## Warning in P_TRT(x = tsdL_Lo_Global_flexit, replicate.CI = 10000): Use results
## with caution; it is probably better to use MCMC

## lower.limit.TRT higher.limit.TRT TRT PT
## 2.5% 27.10014 30.41688 1.402533 30.35651
## 50% 28.64192 30.66392 2.090492 30.56463
## 97.5% 29.19449 31.16977 3.768957 30.77281

SEfromHessian(tsdL_Lo_Global_flexit$hessian)

## P S K1 K2
## 0.1090204 0.2951628 1.5879607 2477.5766337

abs(SEfromHessian(tsdL_Lo_Global_flexit$hessian)/tsdL_Lo_Global_flexit$par)

## P S K1 K2
## 0.003566351 0.372684228 0.924355140 12.387872493

cairo_pdf(filename = "Figure 2.pdf", width=7, height = 9, pointsize = 14)
layout(1:2)
plot(tsdL_Lo_Global_logistic, xlim=c(24, 34))
text(x=24, y=0.4, labels = expression(bold("Logistic")), pos=4, cex=1.1)
text(x=24, y=0.2, labels = paste("AICc=", round(x = comp0["logistic", "AICc"], digits = 2)), pos=4)
text(x=24, y=0.1, labels = paste("Akaike weight=", round(x = comp0["logistic", "Akaike_weight"], digits = 2)), pos=4)

plot(tsdL_Lo_Global_flexit, xlim=c(24, 34))
text(x=24, y=0.4, labels = expression(bold("Flexit")), pos=4, cex=1.1)
text(x=24, y=0.2, labels = paste("AICc=", round(x = comp0["flexit", "AICc"], digits = 2)), pos=4)
text(x=24, y=0.1, labels = paste("Akaike weight=", round(x = comp0["flexit", "Akaike_weight"], digits = 2)), pos=4)

dev.off()

# Fit TSD model for each region when it is possible with logistic model

tsdL_Lo_PacificE_CostaRica_logistic <- with (Lo_PacificE_CostaRica, tsd(males=Males,
 females=Females,
 temperatures=Incubation.temperature,
 equation="logistic",
 parameters.initial = tsdL_Lo_Global_logistic$par))

## [1] "The pivotal temperature is 30.543 CI95% 30.265;30.812"
## [1] "The transitional range of temperatures is 2.680 CI95% 1.774;3.613"
## [1] "The lower limit of transitional range of temperatures is 29.200 CI95% 28.740;29.660"
## [1] "The higher limit of transitional range of temperatures is 31.882 CI95% 31.294;32.481"

tsdL_Lo_PacificE_CostaRica_logistic$pvalue

## [1] 0.01803247

tsdL_Lo_PacificE_CostaRica_logistic$NullDeviancePvalue

## [1] 0.302

tsdL_Lo_PacificE_CostaRica_logistic$deviance

## [1] 15.30215

tsdL_Lo_PacificE_Mexico_logistic <- with (Lo_PacificE_Mexico, tsd(males=Males, females=Females,
 temperatures=Incubation.temperature,
 equation="logistic",
 parameters.initial = tsdL_Lo_Global_logistic$par))

## SE for S too high in 8622 cases out of 18622

## Warning in P_TRT(x = result, l = l, replicate.CI = replicate.CI, probs = c((1
## - : Use results with caution; it is probably better to use MCMC

## [1] "The pivotal temperature is 31.390 CI95% 30.057;34.581"
## [1] "The transitional range of temperatures is 6.189 CI95% 0.294;20.374"
## [1] "The lower limit of transitional range of temperatures is 28.293 CI95% 24.386;29.928"
## [1] "The higher limit of transitional range of temperatures is 34.492 CI95% 30.206;44.788"

tsdL_Lo_PacificE_Mexico_logistic$pvalue

## [1] 1

tsdL_Lo_PacificE_Mexico_logistic$NullDeviancePvalue

## [1] 0.412

tsdL_Lo_PacificE_Mexico_logistic$deviance

## [1] 4.783007e-05

tsdL_Lo_PacificE_logistic <- with (Lo_PacificE, tsd(males=Males, females=Females,
 temperatures=Incubation.temperature,
 equation="logistic",
 parameters.initial = tsdL_Lo_Global_logistic$par))

## [1] "The pivotal temperature is 30.475 CI95% 30.275;30.680"
## [1] "The transitional range of temperatures is 2.270 CI95% 1.656;2.881"
## [1] "The lower limit of transitional range of temperatures is 29.343 CI95% 29.021;29.666"
## [1] "The higher limit of transitional range of temperatures is 31.610 CI95% 31.203;32.020"

tsdL_Lo_PacificE_logistic$pvalue

## [1] 0.545887

tsdL_Lo_PacificE_logistic$NullDeviancePvalue

## [1] 0.291

tsdL_Lo_PacificE_logistic$deviance

## [1] 18.63142

tsdL_Lo_AtlanticWest_logistic <- with (Lo_AtlanticWest, tsd(males=Males, females=Females,
 temperatures=Incubation.temperature,
 equation="logistic",
 parameters.initial = tsdL_Lo_Global_logistic$par))

## SE for S too high in 12 cases out of 10012

## Warning in P_TRT(x = result, l = l, replicate.CI = replicate.CI, probs = c((1
## - : Use results with caution; it is probably better to use MCMC

## [1] "The pivotal temperature is 30.626 CI95% 30.173;31.082"
## [1] "The transitional range of temperatures is 2.129 CI95% 0.785;3.510"
## [1] "The lower limit of transitional range of temperatures is 29.565 CI95% 28.882;30.218"
## [1] "The higher limit of transitional range of temperatures is 31.696 CI95% 30.737;32.648"

tsdL_Lo_AtlanticWest_logistic$pvalue

## [1] 0.9068453

tsdL_Lo_AtlanticWest_logistic$NullDeviancePvalue

## [1] 0.517

tsdL_Lo_AtlanticWest_logistic$deviance

## [1] 4.756401

tsdL_Lo_IndianNE_logistic <- with (Lo_IndianNE, tsd(males=Males, females=Females,
 temperatures=Incubation.temperature,
 equation="logistic", replicate.NullDeviance = 10000,
 parameters.initial = tsdL_Lo_Global_logistic$par))

## SE for S too high in 9096 cases out of 19096

## Warning in P_TRT(x = result, l = l, replicate.CI = replicate.CI, probs = c((1
## - : Use results with caution; it is probably better to use MCMC

## [1] "The pivotal temperature is 29.253 CI95% 28.699;29.506"
## [1] "The transitional range of temperatures is 3.555 CI95% 0.179;11.688"
## [1] "The lower limit of transitional range of temperatures is 27.482 CI95% 22.836;29.400"
## [1] "The higher limit of transitional range of temperatures is 31.034 CI95% 29.577;34.541"

tsdL_Lo_IndianNE_logistic$pvalue

## [1] 1

tsdL_Lo_IndianNE_logistic$NullDeviancePvalue

## [1] 0.449

tsdL_Lo_IndianNE_logistic$deviance

## [1] 1.990495e-05

# Flexit model

tsdL_Lo_PacificE_flexit <- with (Lo_PacificE, tsd(males=Males, females=Females,
 temperatures=Incubation.temperature,
 equation="flexit", replicate.NullDeviance = 10000,
 parameters.initial = tsdL_Lo_Global_flexit$par))

## SE for K1 too high in 10576 cases out of 26059

## SE for K2 too high in 5483 cases out of 26059

## Warning in P_TRT(x = result, l = l, replicate.CI = replicate.CI, probs = c((1
## - : Use results with caution; it is probably better to use MCMC

## [1] "The pivotal temperature is 30.921 CI95% 30.601;31.233"
## [1] "The transitional range of temperatures is 2.809 CI95% 1.966;4.096"
## [1] "The lower limit of transitional range of temperatures is 28.863 CI95% 27.832;29.376"
## [1] "The higher limit of transitional range of temperatures is 31.792 CI95% 30.969;32.378"

P_TRT(tsdL_Lo_PacificE_flexit, replicate.CI = 10000)$P_TRT_quantiles

## SE for K1 too high in 10488 cases out of 25815

## SE for K2 too high in 5327 cases out of 25815

## Warning in P_TRT(tsdL_Lo_PacificE_flexit, replicate.CI = 10000): Use results
## with caution; it is probably better to use MCMC

## lower.limit.TRT higher.limit.TRT TRT PT
## 2.5% 27.82479 30.97285 1.952908 30.60389
## 50% 28.86424 31.77596 2.803181 30.92606
## 97.5% 29.38316 32.37147 4.065541 31.22642

tsdL_Lo_IndianNE_flexit <- with (Lo_IndianNE, tsd(males=Males, females=Females,
 temperatures=Incubation.temperature,
 equation="flexit", replicate.NullDeviance = 10000,
 parameters.initial = tsdL_Lo_Global_flexit$par))

## SE for S too high in 32107 cases out of 64400

## SE for K1 too high in 16280 cases out of 64400

## SE for K2 too high in 6013 cases out of 64400

## Warning in P_TRT(x = result, l = l, replicate.CI = replicate.CI, probs = c((1
## - : Use results with caution; it is probably better to use MCMC

## Warning in P_TRT(x = result, l = l, replicate.CI = replicate.CI, probs = c((1
## - : Something strange occurs; I cannot estimate TRT limits for some replicates

## [1] "The pivotal temperature is 34.345 CI95% 29.698;45.710"
## [1] "The transitional range of temperatures is 7599677882275371510230572556706669715429955688699188483387817984.000 CI95% 0.000;410648237989152069141220457970793381021776406951471617752208999654773477038407418722803537442843257986862530757644988791920121337257637426654309876573864744178198438326098001745075377274880.000"
## [1] "The lower limit of transitional range of temperatures is -7599677882275371510230572556706669715429955688699188483387817984.000 CI95% -410648237989173215890546408408455553987323586786219621482557153267086129429703361435550145917486320039153267037991599392827045717171151968364743103802316969374459650191954871624094091051008.000;40.136"
## [1] "The higher limit of transitional range of temperatures is 34.346 CI95% 29.724;45.710"

P_TRT(tsdL_Lo_IndianNE_flexit, replicate.CI = 10000)$P_TRT_quantiles

## SE for S too high in 31625 cases out of 63708

## SE for K1 too high in 16003 cases out of 63708

## SE for K2 too high in 6080 cases out of 63708

## Warning in P_TRT(tsdL_Lo_IndianNE_flexit, replicate.CI = 10000): Use results
## with caution; it is probably better to use MCMC

## Warning in P_TRT(tsdL_Lo_IndianNE_flexit, replicate.CI = 10000): Something
## strange occurs; I cannot estimate TRT limits for some replicates

## lower.limit.TRT higher.limit.TRT TRT PT
## 2.5% -3.459716e+187 29.68145 2.213332e-04 29.65421
## 50% -2.370347e+61 34.37308 2.370347e+61 34.37234
## 97.5% 4.034497e+01 45.79762 3.459716e+187 45.79740

tsdL_Lo_AtlanticWest_flexit <- with (Lo_AtlanticWest, tsd(males=Males, females=Females,
 temperatures=Incubation.temperature,
 equation="flexit", replicate.NullDeviance = 10000,
 parameters.initial = tsdL_Lo_Global_flexit$par))

## [1] "Convergence is not acheived. Optimization continues !"
## [1] "Convergence is not acheived. Optimization continues !"
## [1] "Convergence is not acheived. Optimization continues !"
## [1] "Convergence is not acheived. Optimization continues !"
## [1] "Convergence is not acheived. Optimization continues !"
## [1] "Convergence is not acheived. Optimization continues !"
## [1] "Convergence is not acheived. Optimization continues !"

## SE for S too high in 13348 cases out of 30314

## SE for K2 too high in 6966 cases out of 30314

## Warning in P_TRT(x = result, l = l, replicate.CI = replicate.CI, probs = c((1
## - : Use results with caution; it is probably better to use MCMC

## [1] "The pivotal temperature is 30.356 CI95% 30.122;30.567"
## [1] "The transitional range of temperatures is 18.107 CI95% 0.430;733776.923"
## [1] "The lower limit of transitional range of temperatures is 12.282 CI95% -733746.671;29.969"
## [1] "The higher limit of transitional range of temperatures is 30.357 CI95% 30.122;30.573"

P_TRT(tsdL_Lo_AtlanticWest_flexit, replicate.CI = 10000)$P_TRT_quantiles

## SE for S too high in 13350 cases out of 30212

## SE for K2 too high in 6862 cases out of 30212

## Warning in P_TRT(tsdL_Lo_AtlanticWest_flexit, replicate.CI = 10000): Use results
## with caution; it is probably better to use MCMC

## lower.limit.TRT higher.limit.TRT TRT PT
## 2.5% -1.029104e+06 30.12459 4.089526e-01 30.12438
## 50% 1.451637e+01 30.36147 1.580428e+01 30.36045
## 97.5% 2.998687e+01 30.57352 1.029134e+06 30.56836

# Plot the results

## Pacific East

plot(tsdL_Lo_PacificE_logistic)


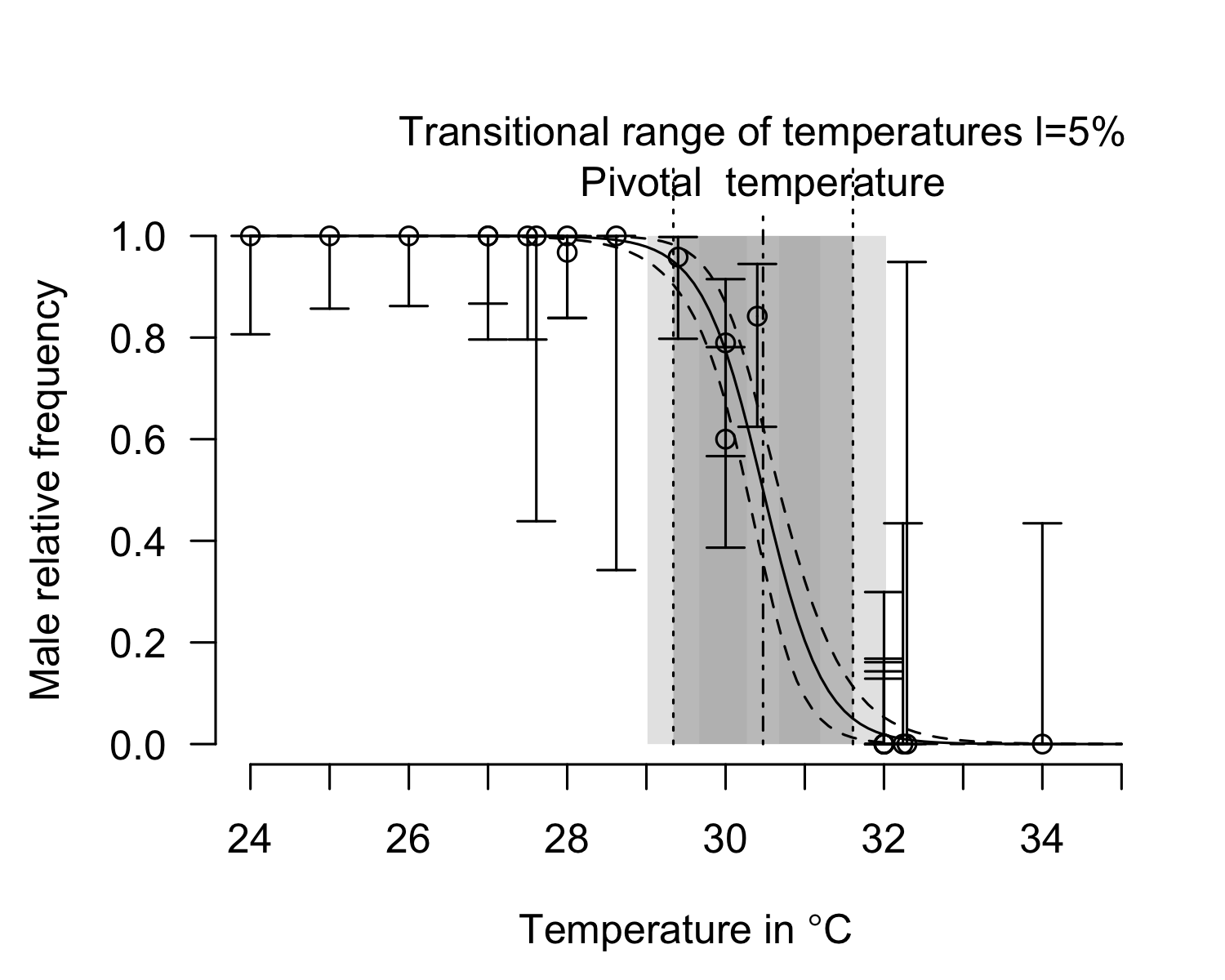


P_TRT(tsdL_Lo_PacificE_logistic, replicate.CI = 10000)$P_TRT_quantiles

## lower.limit.TRT higher.limit.TRT TRT PT
## 2.5% 29.00486 31.21280 1.661515 30.27633
## 50% 29.34131 31.60701 2.266305 30.47359
## 97.5% 29.67068 32.01124 2.900756 30.67505

## Pacific East - Mexico

plot(tsdL_Lo_PacificE_Mexico_logistic)

## SE for S too high in 9026 cases out of 19026

## Warning in P_TRT(x = x, resultmcmc = resultmcmc, chain = chain, l = l,
## replicate.CI = replicate.CI, : Use results with caution; it is probably better
## to use MCMC


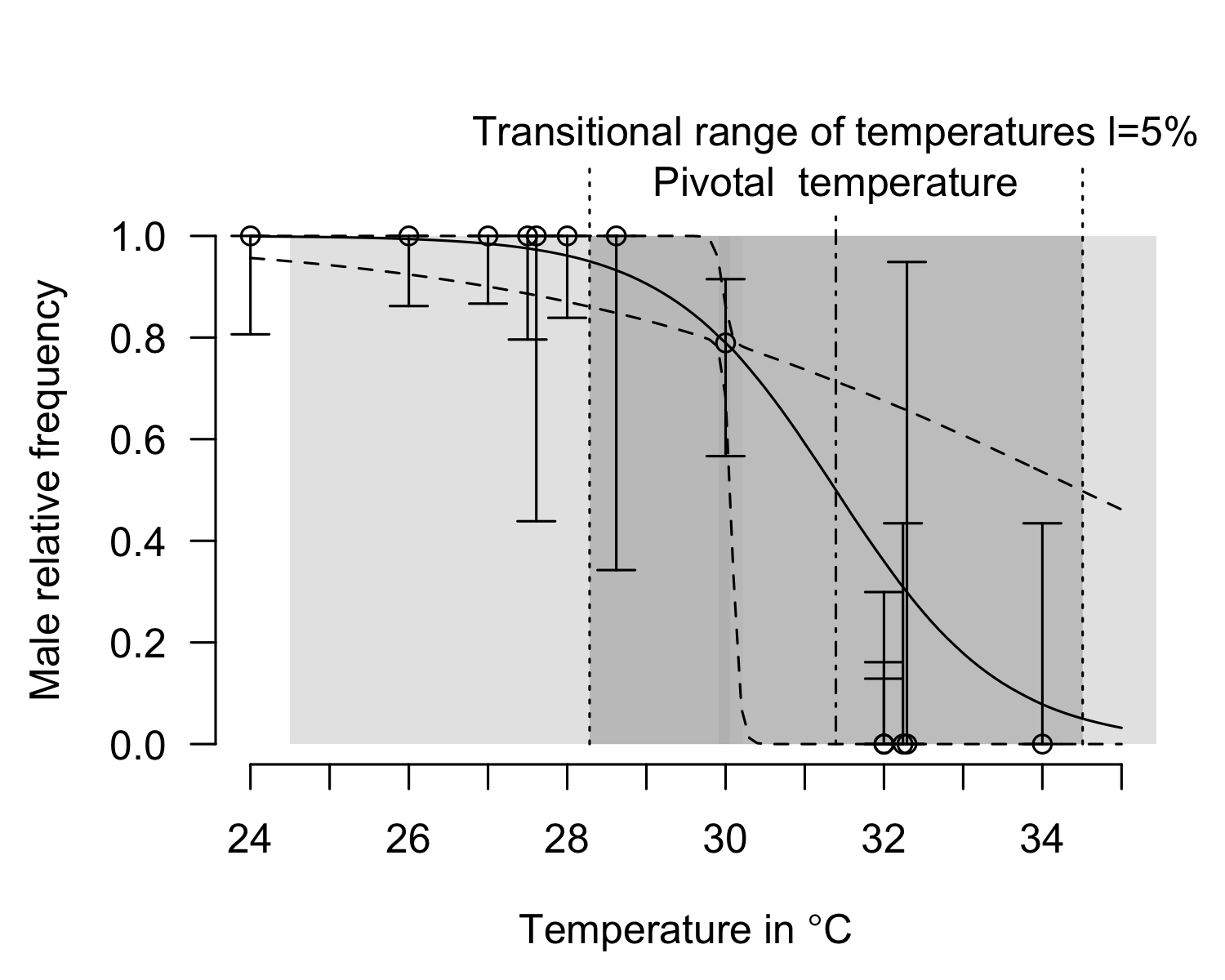


P_TRT(tsdL_Lo_PacificE_Mexico_logistic)$P_TRT_quantiles

## lower.limit.TRT higher.limit.TRT TRT PT
## 2.5% 29.80147 30.52199 0.7205173 30.16173
## 50% 29.80147 30.52199 0.7205173 30.16173
## 97.5% 29.80147 30.52199 0.7205173 30.16173

## Pacific East - Costa Rica

plot(tsdL_Lo_PacificE_CostaRica_logistic)


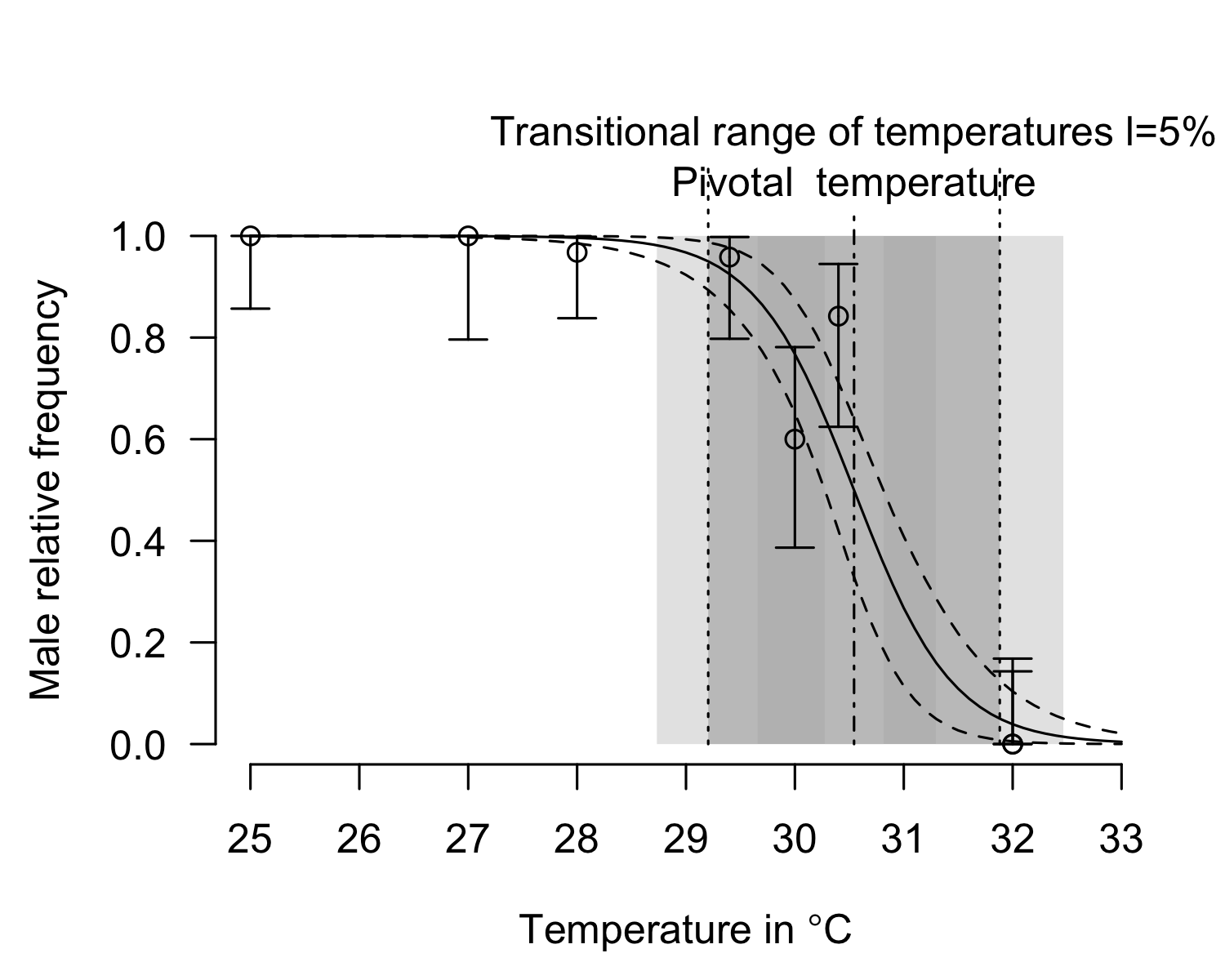


P_TRT(tsdL_Lo_PacificE_CostaRica_logistic)$P_TRT_quantiles

## lower.limit.TRT higher.limit.TRT TRT PT
## 2.5% 29.20072 31.88186 2.681144 30.54129
## 50% 29.20072 31.88186 2.681144 30.54129
## 97.5% 29.20072 31.88186 2.681144 30.54129

## Atlantic West

plot(tsdL_Lo_AtlanticWest_logistic)

## SE for S too high in 16 cases out of 10016

## Warning in P_TRT(x = x, resultmcmc = resultmcmc, chain = chain, l = l,
## replicate.CI = replicate.CI, : Use results with caution; it is probably better
## to use MCMC


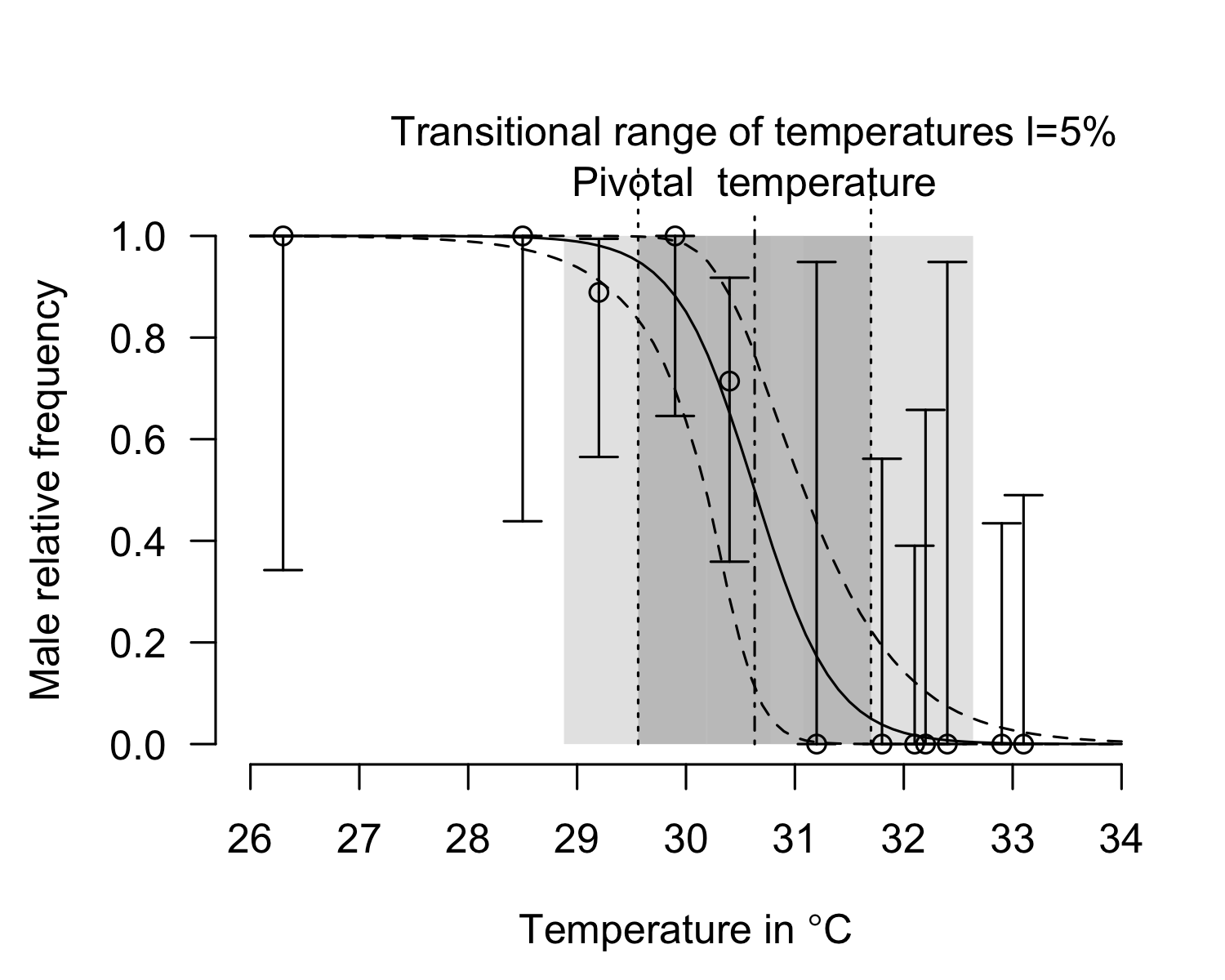


P_TRT(tsdL_Lo_AtlanticWest_logistic, replicate.CI = 10000)$P_TRT_quantiles

## SE for S too high in 3 cases out of 10003

## Warning in P_TRT(tsdL_Lo_AtlanticWest_logistic, replicate.CI = 10000): Use
## results with caution; it is probably better to use MCMC

## lower.limit.TRT higher.limit.TRT TRT PT
## 2.5% 28.88995 30.74434 0.7911599 30.18619
## 50% 29.56147 31.69772 2.1338217 30.62894
## 97.5% 30.21697 32.62973 3.4875107 31.08716

## Indian Ocean

plot(x=tsdL_Lo_IndianNE_logistic, xlim=c(24, 34))

## SE for S too high in 9384 cases out of 19384

## Warning in P_TRT(x = x, resultmcmc = resultmcmc, chain = chain, l = l,
## replicate.CI = replicate.CI, : Use results with caution; it is probably better
## to use MCMC


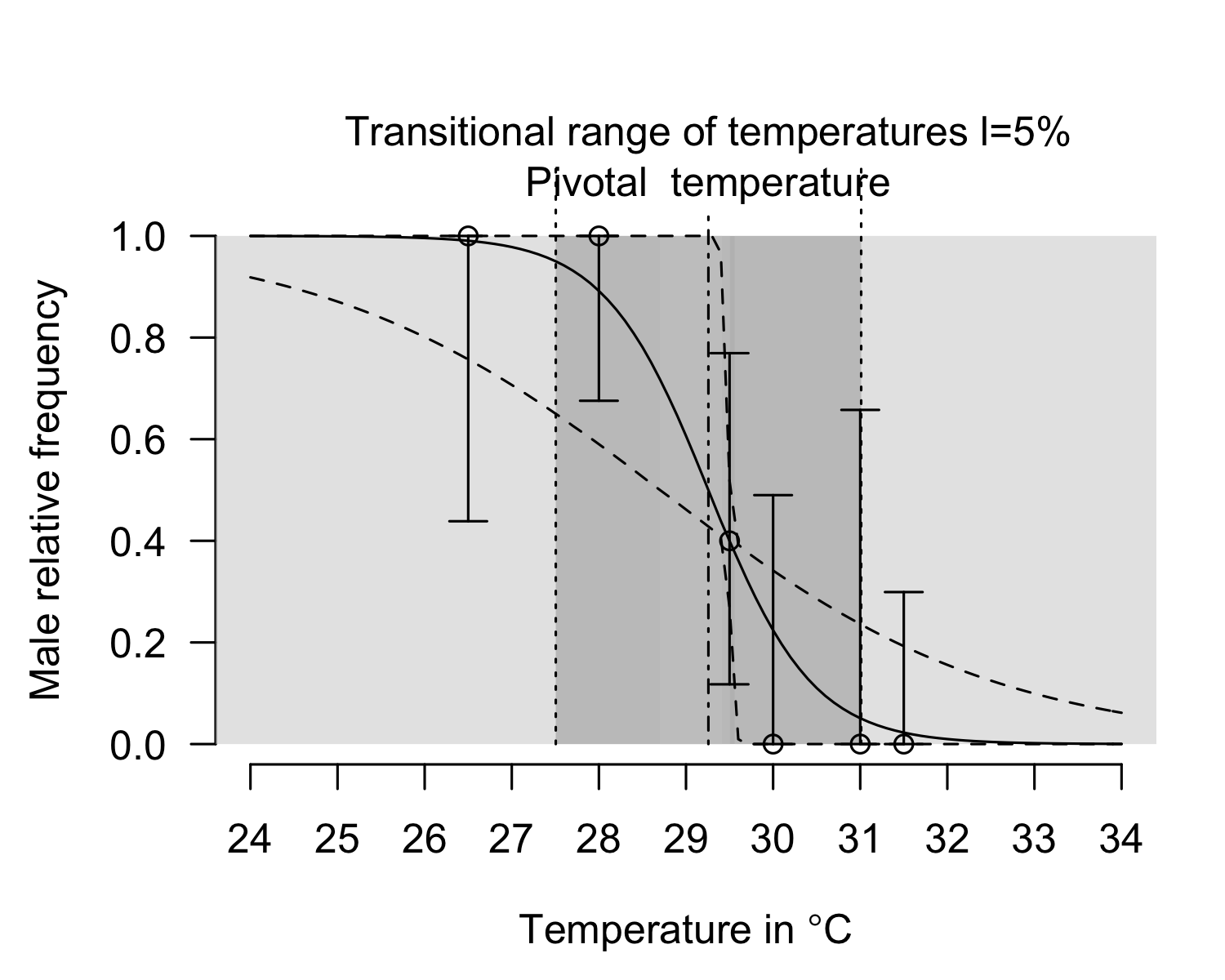


P_TRT(tsdL_Lo_IndianNE_logistic, replicate.CI = 10000)$P_TRT_quantiles

## SE for S too high in 9410 cases out of 19410

## Warning in P_TRT(tsdL_Lo_IndianNE_logistic, replicate.CI = 10000): Use results
## with caution; it is probably better to use MCMC

## lower.limit.TRT higher.limit.TRT TRT PT
## 2.5% 22.88738 29.57203 0.1610786 28.69708
## 50% 27.46949 31.04330 3.5695871 29.25211
## 97.5% 29.41138 34.48979 11.5958544 29.50784

## Plot of all series

pdf(file = "Figure 4.pdf", width=10, height = 14, pointsize = 14)
layout(mat = matrix(1:6, ncol=2, byrow = FALSE))
plot(tsdL_Lo_PacificE_logistic, xlim=c(24, 34))
text(x=ScalePreviousPlot()$xlim["begin"]+ScalePreviousPlot()$xlim["range"]*0.02,
 y=ScalePreviousPlot()$ylim["begin"]+ScalePreviousPlot()$ylim["range"]*0.1, labels="East Pacific", cex=2, pos=4)
plot(tsdL_Lo_PacificE_Mexico_logistic, xlim=c(24, 34))
text(x=ScalePreviousPlot()$xlim["begin"]+ScalePreviousPlot()$xlim["range"]*0.02,
 y=ScalePreviousPlot()$ylim["begin"]+ScalePreviousPlot()$ylim["range"]*0.1, labels="Mexico", cex=2, pos=4)

plot(tsdL_Lo_PacificE_CostaRica_logistic, xlim=c(24, 34))
text(x=ScalePreviousPlot()$xlim["begin"]+ScalePreviousPlot()$xlim["range"]*0.02,
 y=ScalePreviousPlot()$ylim["begin"]+ScalePreviousPlot()$ylim["range"]*0.1, labels="Costa Rica", cex=2, pos=4)

plot(tsdL_Lo_AtlanticWest_logistic, xlim=c(24, 34))
text(x=ScalePreviousPlot()$xlim["begin"]+ScalePreviousPlot()$xlim["range"]*0.02,
 y=ScalePreviousPlot()$ylim["begin"]+ScalePreviousPlot()$ylim["range"]*0.1, labels="West Atlantic", cex=2, pos=4)

plot(x=tsdL_Lo_IndianNE_logistic, xlim=c(24, 34))
text(x=ScalePreviousPlot()$xlim["begin"]+ScalePreviousPlot()$xlim["range"]*0.02,
 y=ScalePreviousPlot()$ylim["begin"]+ScalePreviousPlot()$ylim["range"]*0.1, labels="Northeast Indian", cex=2, pos=4)
dev.off()

# Compare a global model to a RMU specific model

A single model for all locations is not selected. It proved that there is differences among locations.

compare_BIC(global=tsdL_Lo_Global_logistic, separate=list(tsdL_Lo_AtlanticWest_logistic, tsdL_Lo_PacificE_logistic, tsdL_Lo_IndianNE_logistic))

## [1] "The lowest BIC (63.874) is for series separate with Akaike weight=0.983"

## BIC DeltaBIC Akaike_weight
## global 71.99428 8.119845 0.01695783
## separate 63.87443 0.000000 0.98304217

A single model for Mexican and Costa Rican locations is selected. It proved that there is no difference among Pacific East locations.

compare_BIC(PacificE=tsdL_Lo_PacificE_logistic, separate=list(tsdL_Lo_PacificE_Mexico_logistic, tsdL_Lo_PacificE_CostaRica_logistic))

## [1] "The lowest BIC (37.259) is for series PacificE with Akaike weight=0.820"

## BIC DeltaBIC Akaike_weight
## PacificE 37.25886 0.000000 0.8199806
## separate 40.29129 3.032432 0.1800194

# Bayesian model to compare Costa Rica and Mexico and other locations with logistic model

For all locations, priors are obtained from the global model.

## Costa Rica, East Pacific

pMCMC_LoPacificE_CostaRica <- tsd_MHmcmc_p(tsdL_Lo_PacificE_CostaRica_logistic, accept=TRUE)
pMCMC_LoPacificE_CostaRica[, "Prior1"] <- tsdL_Lo_Global_logistic$par


result_mcmc_tsd_LoPacificE_CostaRica <- tsd_MHmcmc(result=tsdL_Lo_PacificE_CostaRica_logistic,
 parametersMCMC=pMCMC_LoPacificE_CostaRica, n.iter=100000,
 n.chains = 1,
 n.adapt = 0, thin=1, trace=FALSE, adaptive = TRUE)

## Density Prior1 Prior2 SDProp Min Max Init
## P dnorm 30.3854857 2 2.0 25 35 30.5412874
## S dnorm -0.4102641 1 0.5 -2 2 -0.4552895
## Chain 1
## Best likelihood for:
## P = 30.5412874399076
## S = -0.455289504305702

plot(tsdL_Lo_PacificE_CostaRica_logistic, resultmcmc = result_mcmc_tsd_LoPacificE_CostaRica)


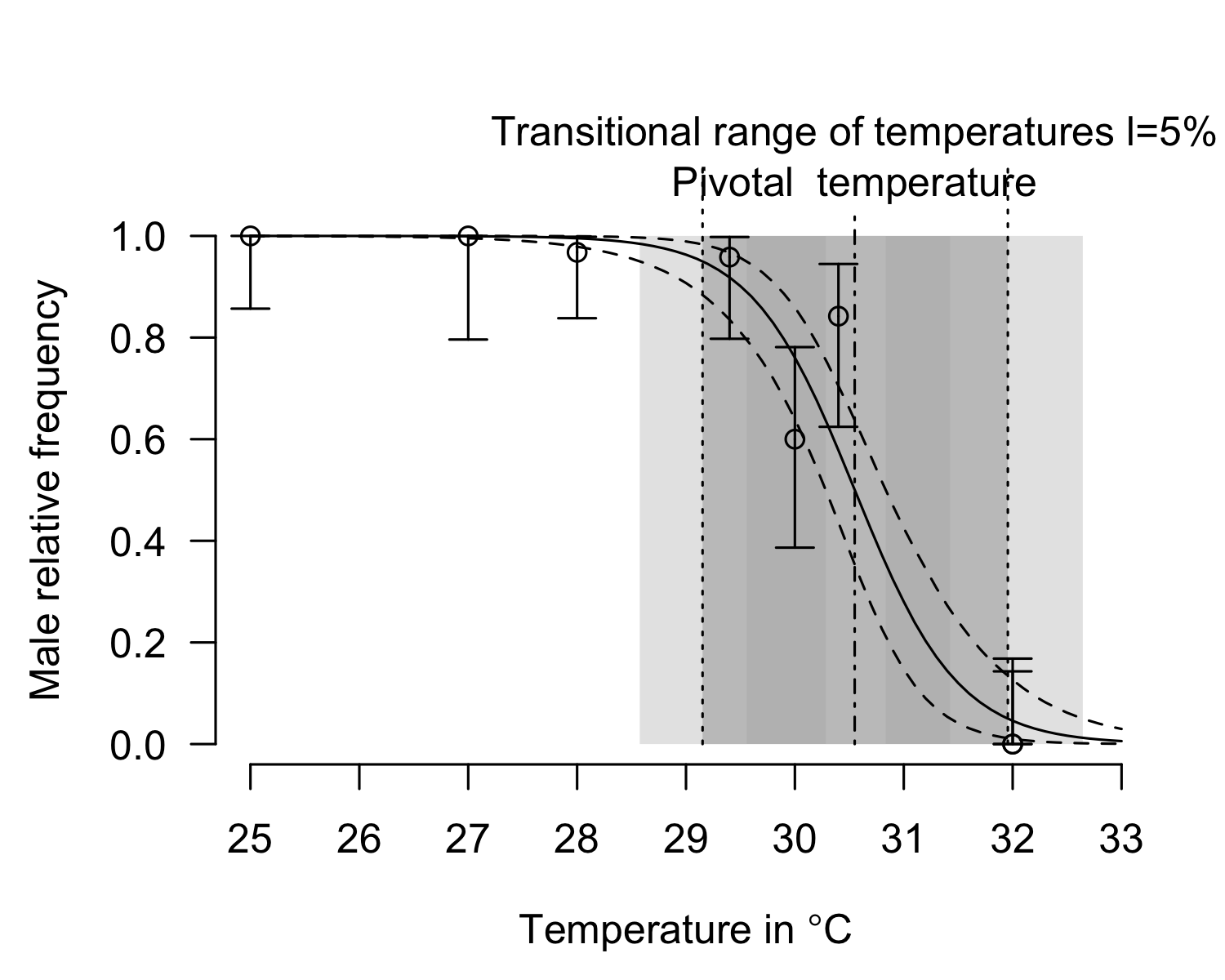


plot(result_mcmc_tsd_LoPacificE_CostaRica, parameters = "S", xlim=c(-2, 2))


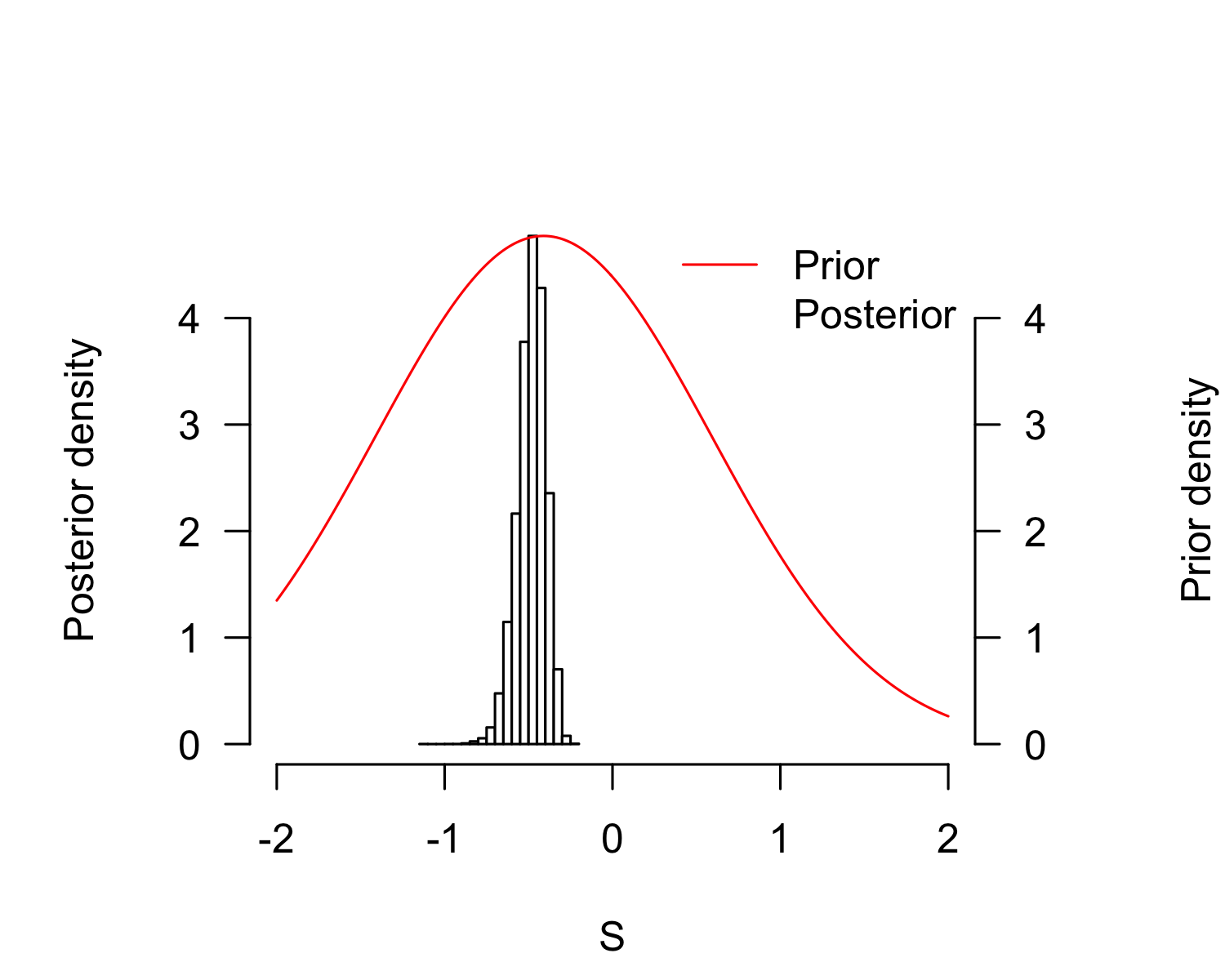


plot(result_mcmc_tsd_LoPacificE_CostaRica, parameters = "P", xlim=c(25, 35))


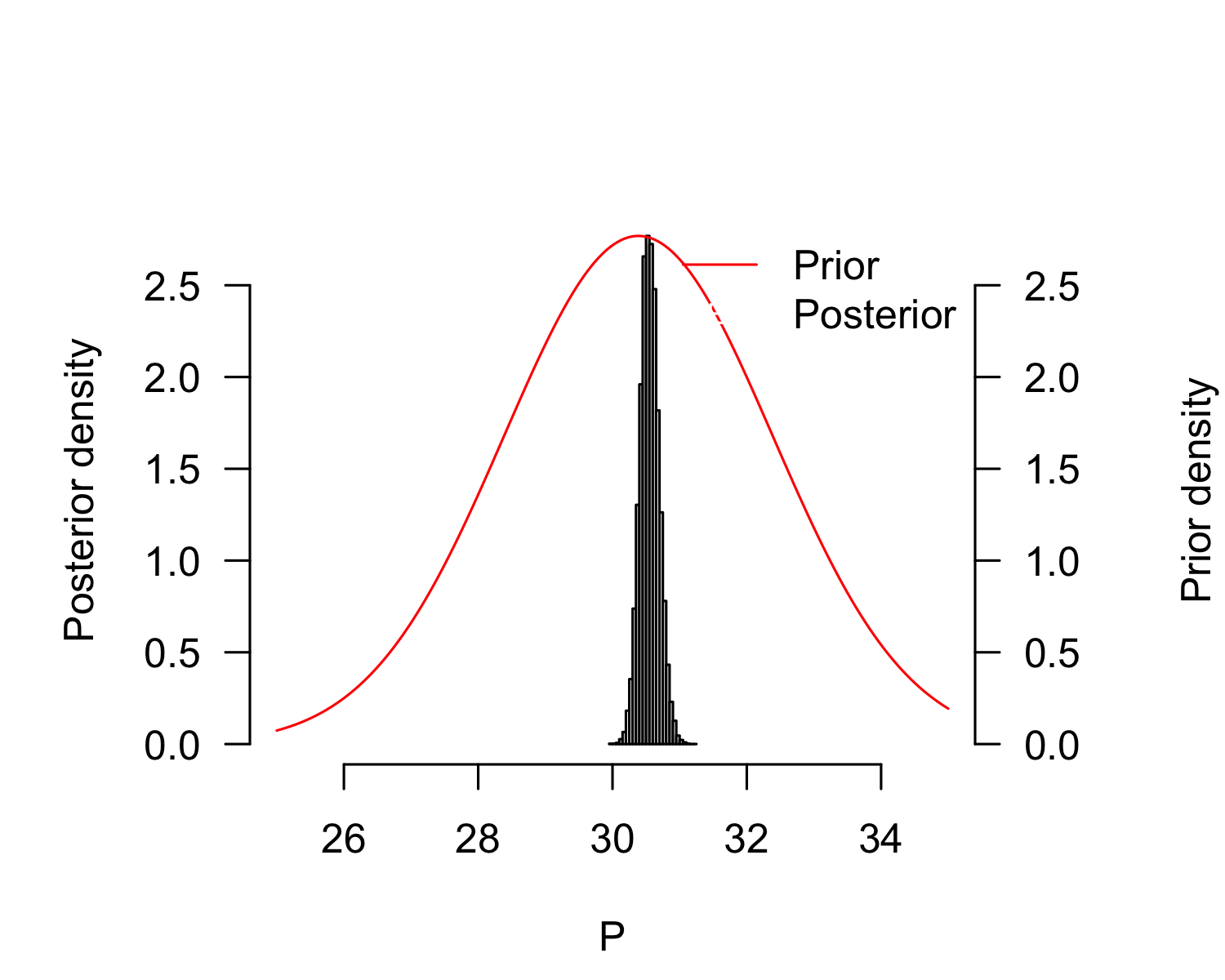


P_TRT_result_mcmc_tsd_LoPacificE_CostaRica <- P_TRT(resultmcmc = result_mcmc_tsd_LoPacificE_CostaRica,
 equation = "logistic", replicate.CI = 100000)
P_TRT_result_mcmc_tsd_LoPacificE_CostaRica$P_TRT_quantiles

## lower.limit.TRT higher.limit.TRT TRT PT
## 2.5% 28.56808 31.42271 2.006444 30.28309
## 50% 29.14930 31.95603 2.808647 30.54813
## 97.5% 29.55650 32.64471 3.939233 30.83732

## Mexico, East Pacific

pMCMC_LoPacificE_Mexico <- tsd_MHmcmc_p(tsdL_Lo_PacificE_Mexico_logistic, accept=TRUE)
pMCMC_LoPacificE_Mexico[, "Prior1"] <- tsdL_Lo_Global_logistic$par


result_mcmc_tsd_LoPacificE_Mexico <- tsd_MHmcmc(result=tsdL_Lo_PacificE_Mexico_logistic,
 parametersMCMC=pMCMC_LoPacificE_Mexico, n.iter=100000,
 n.chains = 1,
 n.adapt = 0, thin=1, trace=FALSE, adaptive = TRUE)

## Density Prior1 Prior2 SDProp Min Max Init
## P dnorm 30.3854857 2 2.0 25 35 30.1617279
## S dnorm -0.4102641 1 0.5 -2 2 -0.1223522
## Chain 1
## Best likelihood for:
## P = 30.0677471976356
## S = -0.0512703533381977

plot(tsdL_Lo_PacificE_Mexico_logistic, resultmcmc = result_mcmc_tsd_LoPacificE_Mexico)


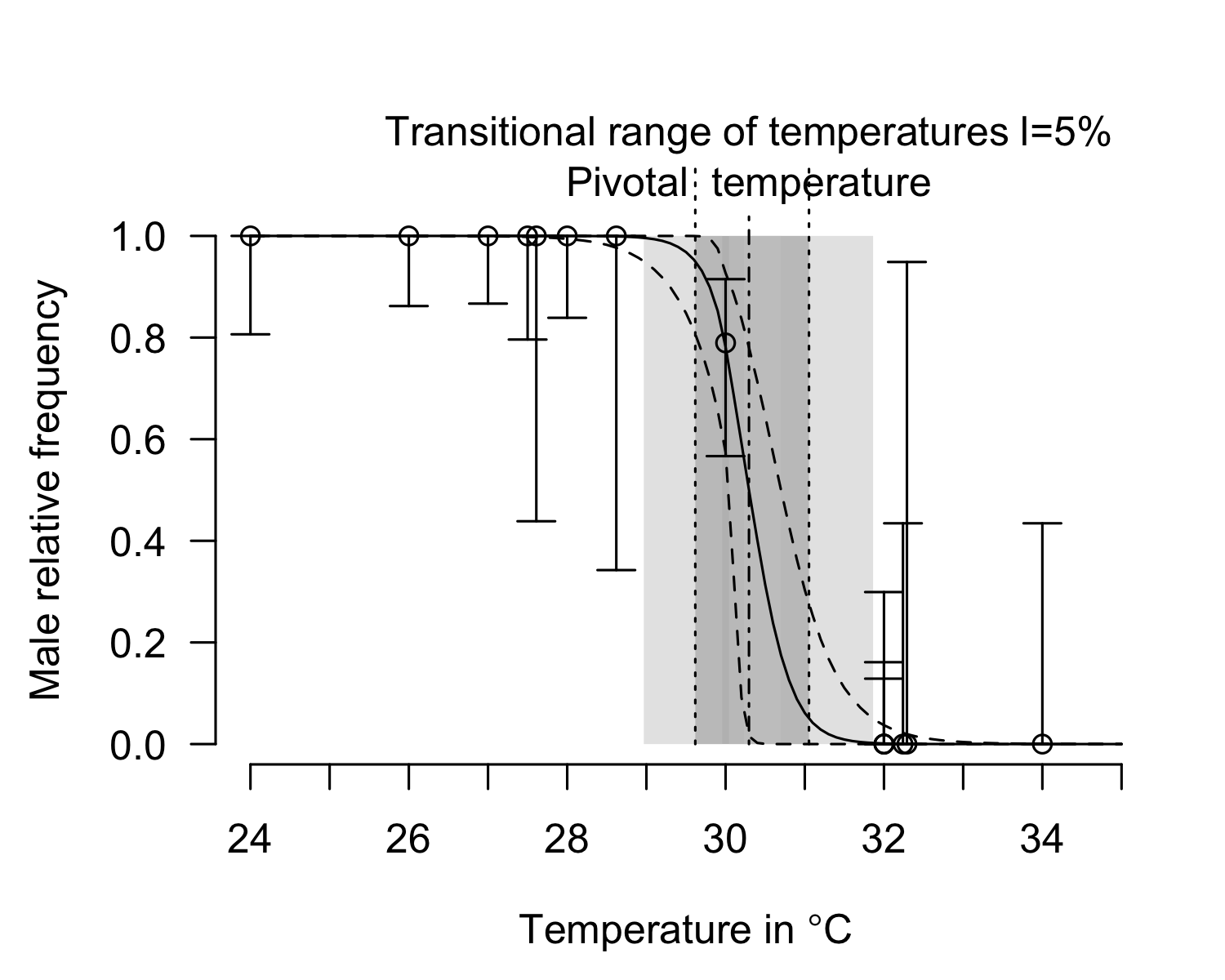


plot(result_mcmc_tsd_LoPacificE_Mexico, parameters = "S", xlim=c(-2, 2))


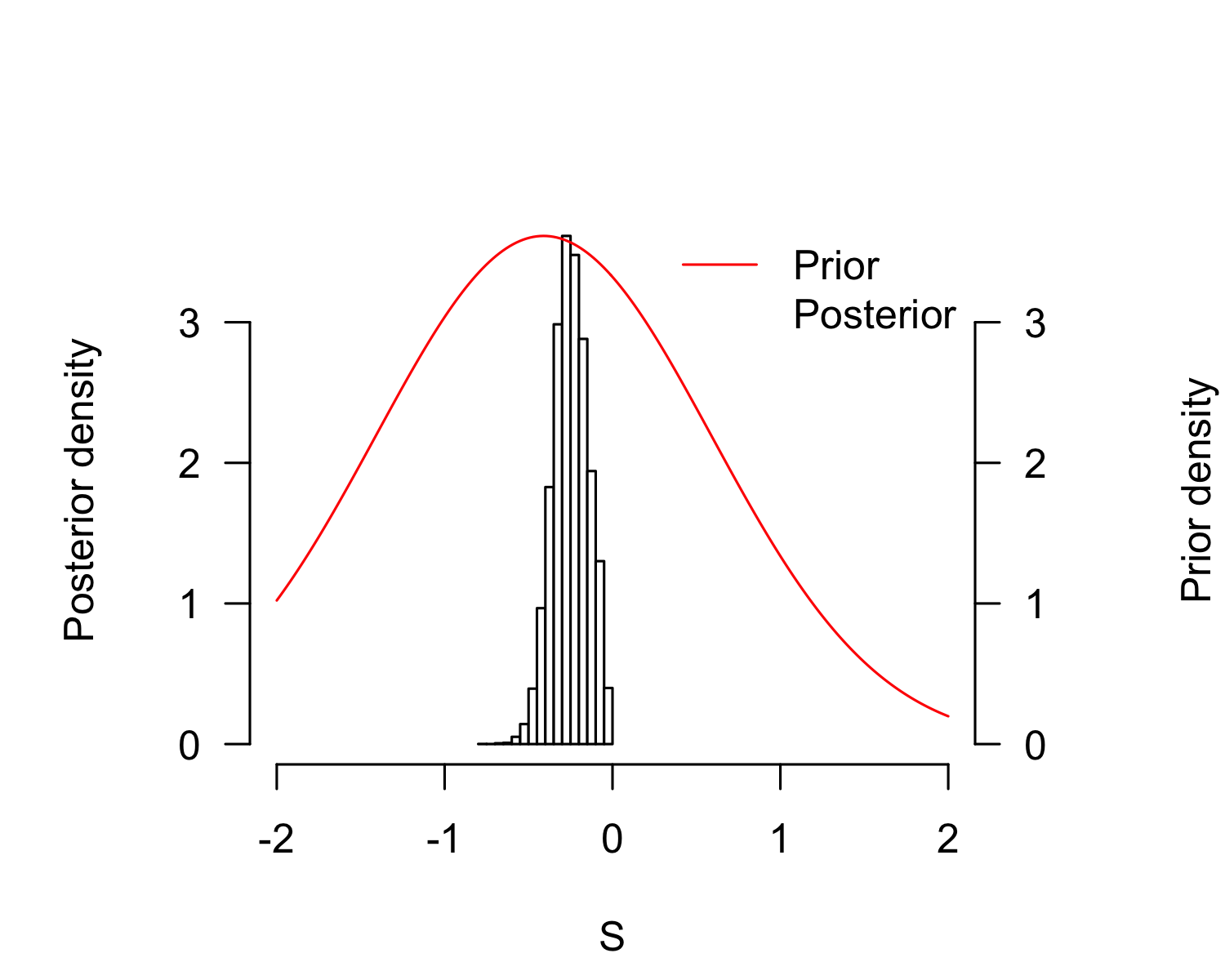


plot(result_mcmc_tsd_LoPacificE_Mexico, parameters = "P", xlim=c(25, 35))


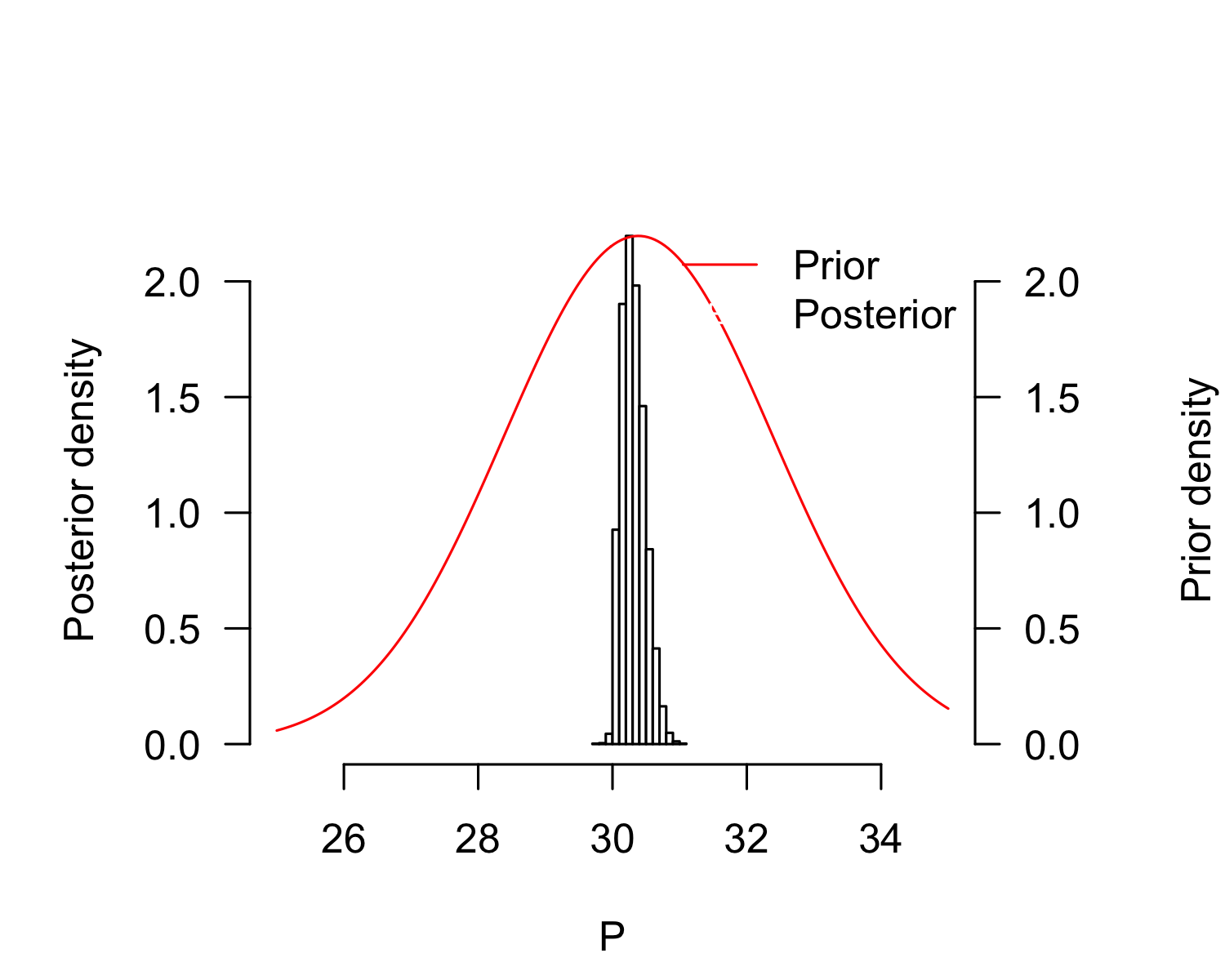


P_TRT_result_mcmc_tsd_LoPacificE_Mexico <- P_TRT(resultmcmc = result_mcmc_tsd_LoPacificE_Mexico,
 equation = "logistic", replicate.CI = 100000)
P_TRT_result_mcmc_tsd_LoPacificE_Mexico$P_TRT_quantiles

## lower.limit.TRT higher.limit.TRT TRT PT
## 2.5% 28.96153 30.23307 0.3234408 30.04076
## 50% 29.61784 31.06423 1.4712989 30.29637
## 97.5% 29.95560 31.86146 2.7050405 30.69299

## Northeast India

pMCMC_LoIndianNE <- tsd_MHmcmc_p(tsdL_Lo_IndianNE_logistic, accept=TRUE)
pMCMC_LoIndianNE[, "Prior1"] <- tsdL_Lo_Global_logistic$par


result_mcmc_tsd_LoIndianNE <- tsd_MHmcmc(result=tsdL_Lo_IndianNE_logistic,
 parametersMCMC=pMCMC_LoIndianNE, n.iter=100000,
 n.chains = 1,
 n.adapt = 0, thin=1, trace=FALSE, adaptive = TRUE)

## Density Prior1 Prior2 SDProp Min Max Init
## P dnorm 30.3854857 2 2.0 25 35 29.48378094
## S dnorm -0.4102641 1 0.5 -2 2 -0.03999752
## Chain 1
## Best likelihood for:
## P = 29.4899351019249
## S = -0.0250671091818515

plot(tsdL_Lo_IndianNE_logistic, resultmcmc = result_mcmc_tsd_LoIndianNE)


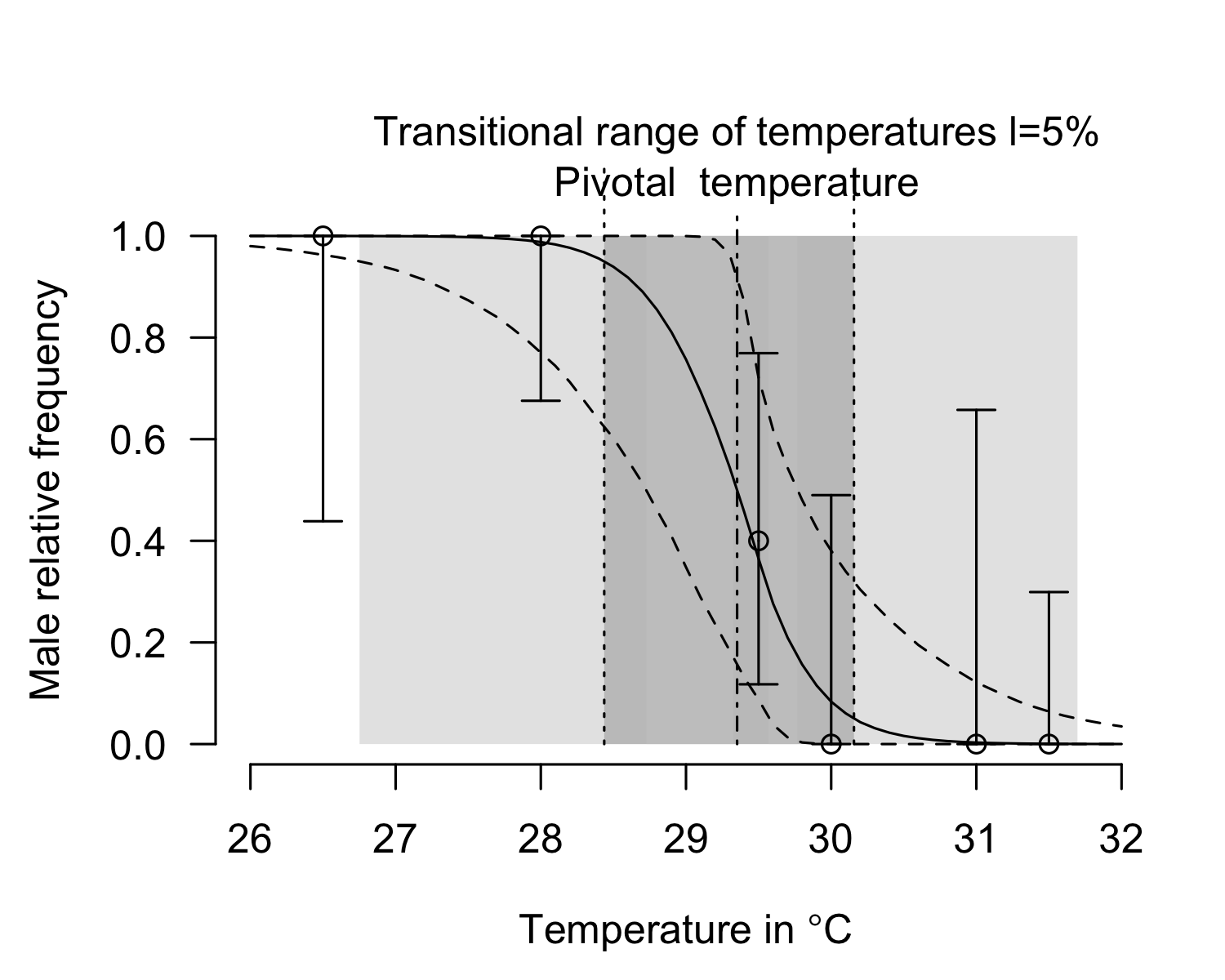


plot(result_mcmc_tsd_LoIndianNE, parameters = "S", xlim=c(-2, 2))


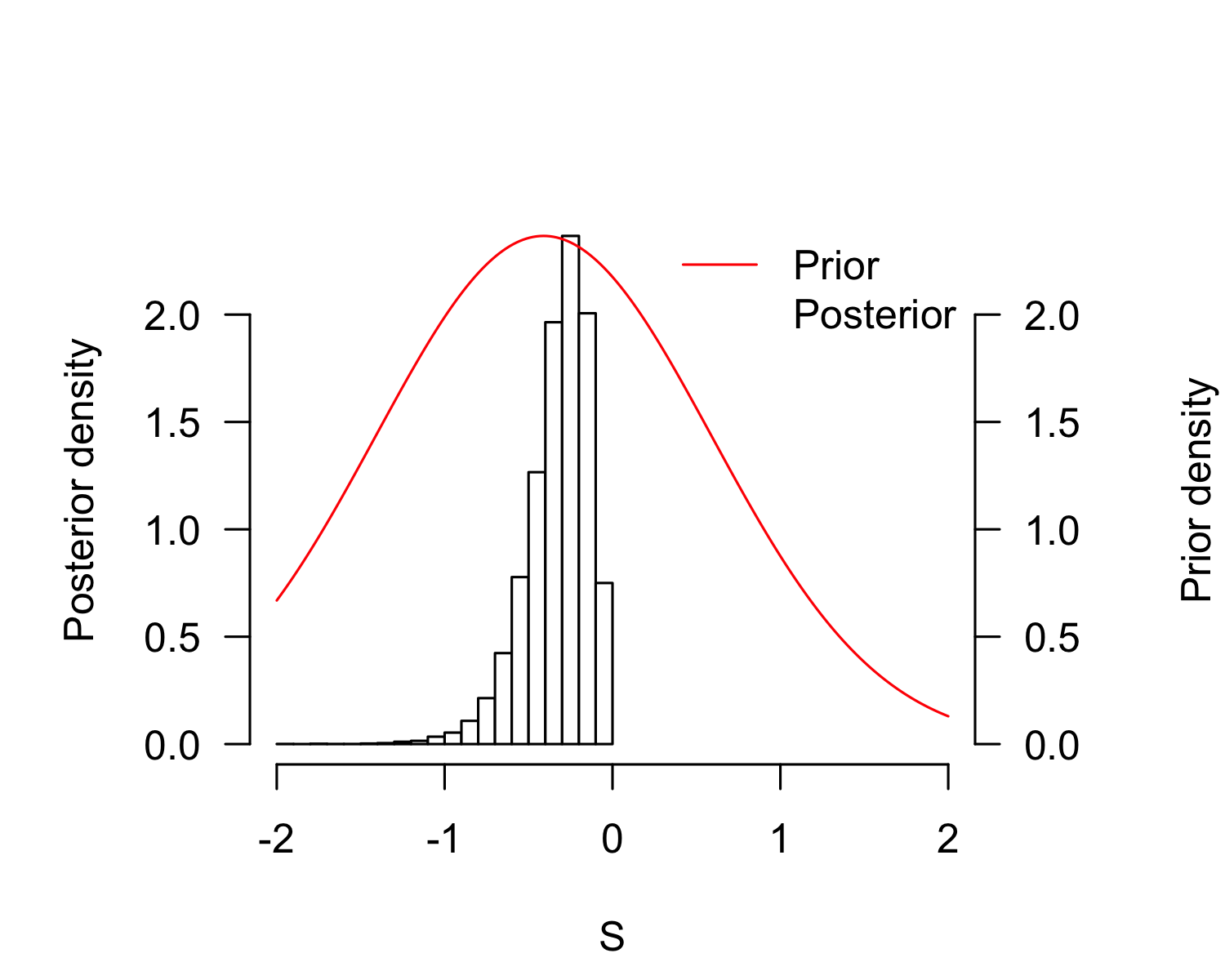


plot(result_mcmc_tsd_LoIndianNE, parameters = "P", xlim=c(25, 35))


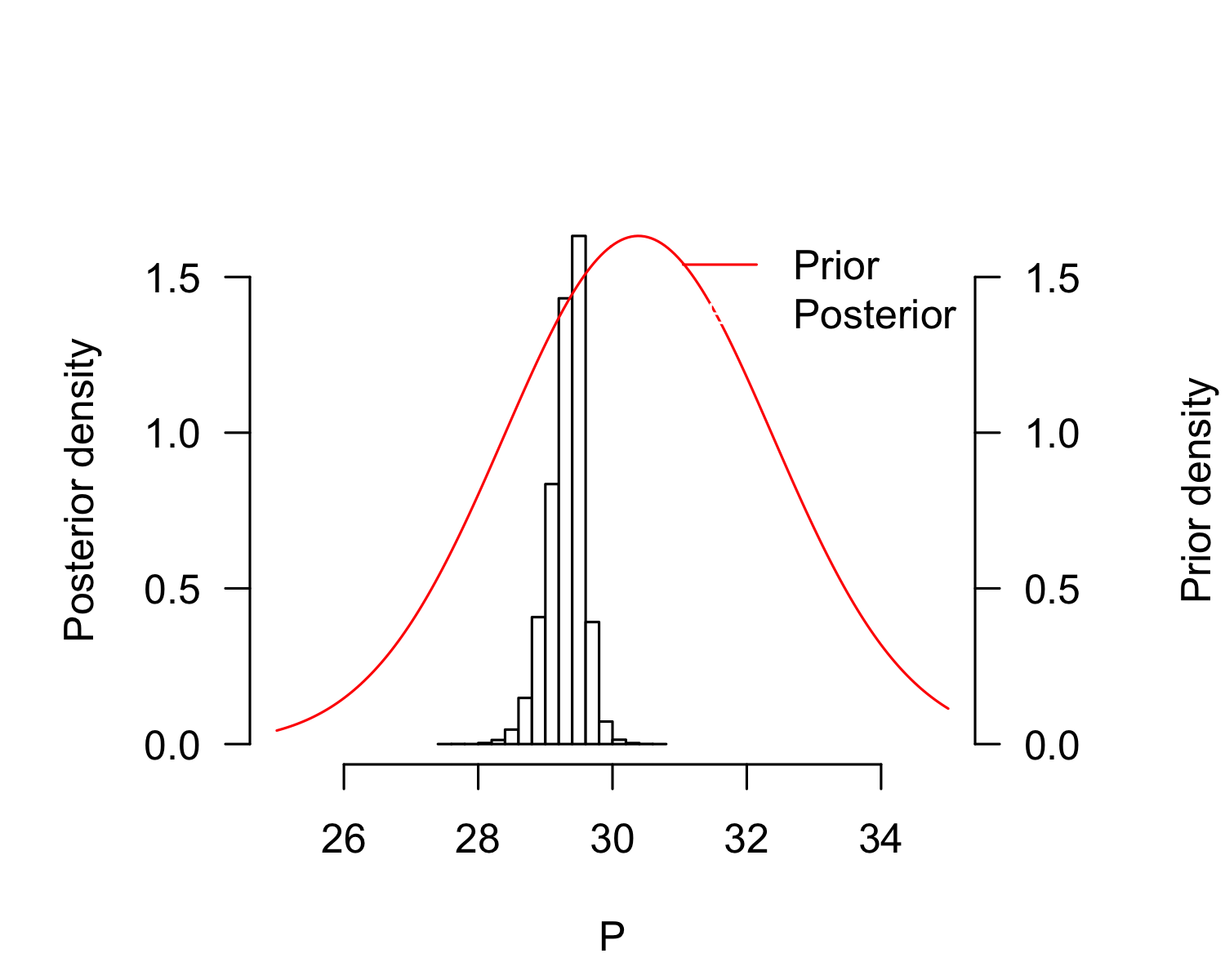


P_TRT_result_mcmc_tsd_LoIndianNE <- P_TRT(resultmcmc = result_mcmc_tsd_LoIndianNE,
 equation = "logistic", replicate.CI = 100000)
P_TRT_result_mcmc_tsd_LoIndianNE$P_TRT_quantiles

## lower.limit.TRT higher.limit.TRT TRT PT
## 2.5% 26.71379 29.56948 0.3310552 28.71002
## 50% 28.44407 30.15611 1.7316376 29.35182
## 97.5% 29.32937 31.66098 4.6805582 29.76256

layout(matrix(1:3, ncol=3))
plot(result_mcmc_tsd_LoIndianNE, parameters = "P", xlim=c(25, 35), legend = FALSE)
text(x=ScalePreviousPlot()$xlim["begin"]+ScalePreviousPlot()$xlim["range"]*0.1,
 y=ScalePreviousPlot()$ylim["end"]*0.95, labels="A", cex=2)
plot(result_mcmc_tsd_LoIndianNE, parameters = "S", xlim=c(-3, 2), legend = FALSE)
text(x=ScalePreviousPlot()$xlim["begin"]+ScalePreviousPlot()$xlim["range"]*0.1,
 y=ScalePreviousPlot()$ylim["end"]*0.95, labels="B", cex=2)
rd <- sample(1:100000, 10000)
plot(as.numeric(result_mcmc_tsd_LoIndianNE$resultMCMC[[1]][rd, "P"]),
 as.numeric(result_mcmc_tsd_LoIndianNE$resultMCMC[[1]][rd, "S"]), pch=".",
 xlab="P", ylab="S", bty="n", las=1)
par(xpd=TRUE)
text(x=ScalePreviousPlot()$xlim["begin"]+ScalePreviousPlot()$xlim["range"]*0.1,
 y=ScalePreviousPlot()$ylim["begin"]+ScalePreviousPlot()$ylim["range"]*0.95, labels="C", cex=2)


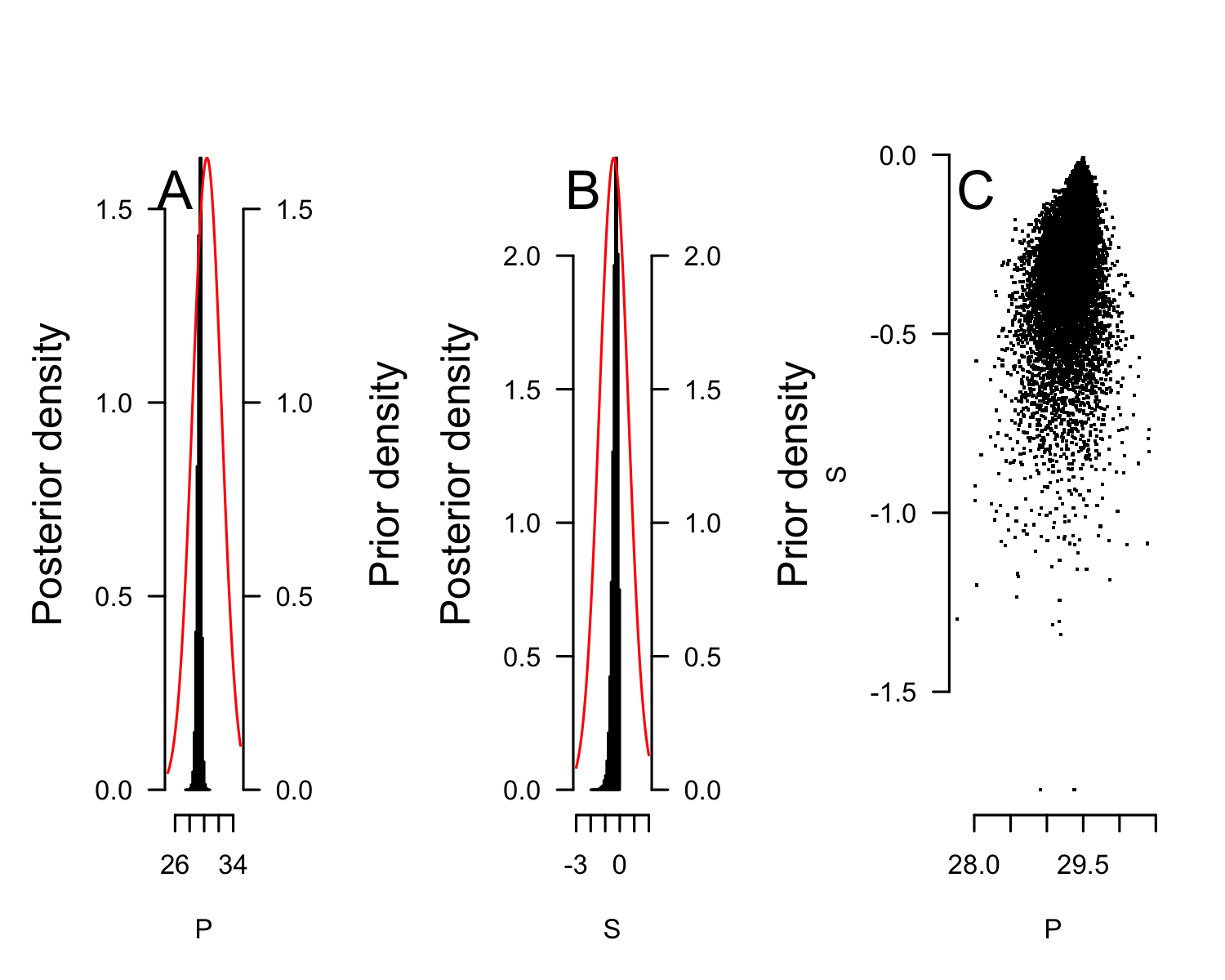


## West Atlantic

pMCMC_LoAtlanticWest <- tsd_MHmcmc_p(tsdL_Lo_AtlanticWest_logistic, accept=TRUE)
pMCMC_LoAtlanticWest[, "Prior1"] <- tsdL_Lo_Global_logistic$par


result_mcmc_tsd_LoAtlanticWest <- tsd_MHmcmc(result=tsdL_Lo_AtlanticWest_logistic,
 parametersMCMC=pMCMC_LoAtlanticWest, n.iter=100000,
 n.chains = 1,
 n.adapt = 0, thin=1, trace=FALSE, adaptive = TRUE)

## Density Prior1 Prior2 SDProp Min Max Init
## P dnorm 30.3854857 2 2.0 25 35 30.6289866
## S dnorm -0.4102641 1 0.5 -2 2 -0.3622671
## Chain 1
## Best likelihood for:
## P = 30.6289866479658
## S = -0.362267124531315

plot(tsdL_Lo_AtlanticWest_logistic, resultmcmc = result_mcmc_tsd_LoAtlanticWest)


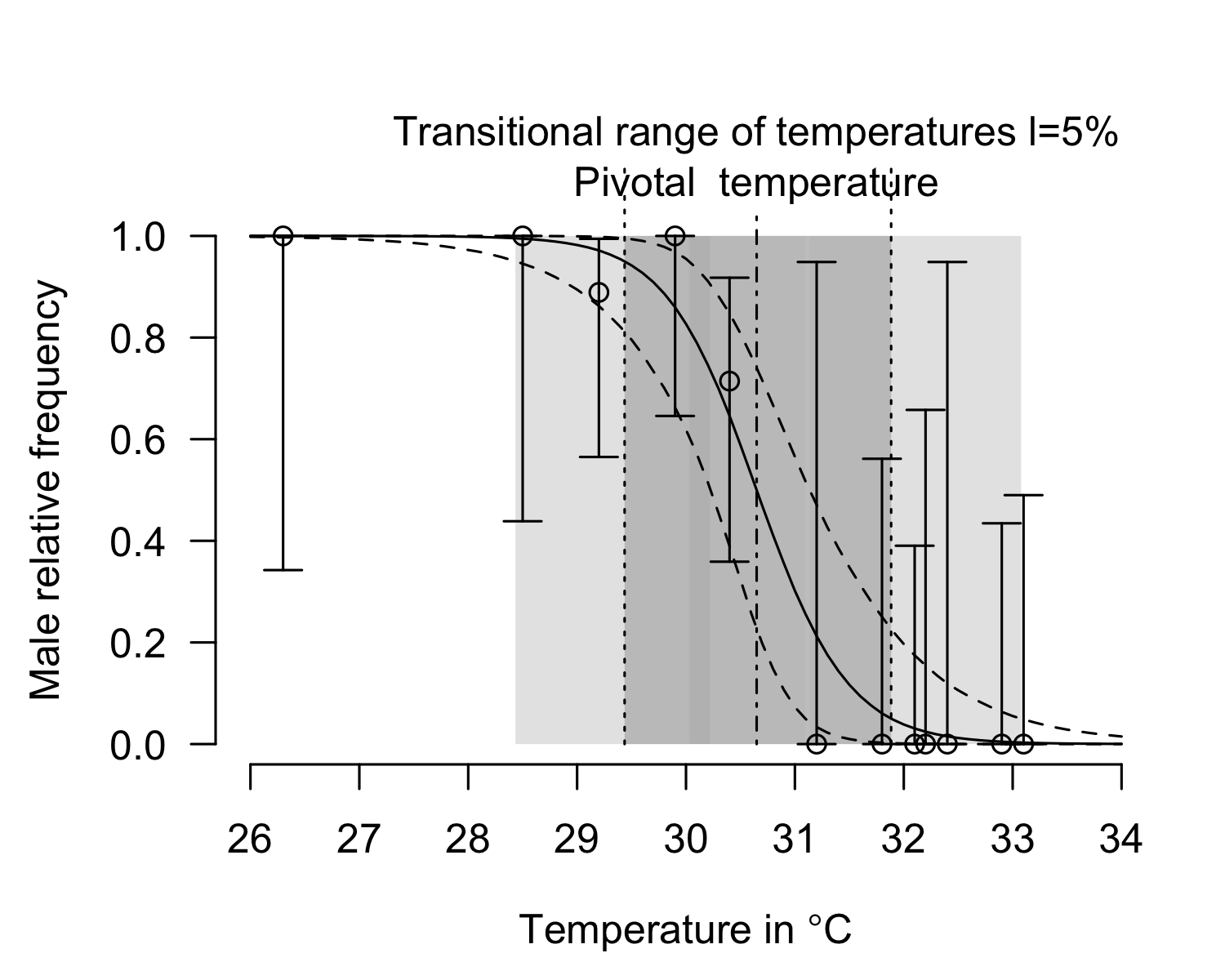


plot(result_mcmc_tsd_LoAtlanticWest, parameters = "S", xlim=c(-2, 2))


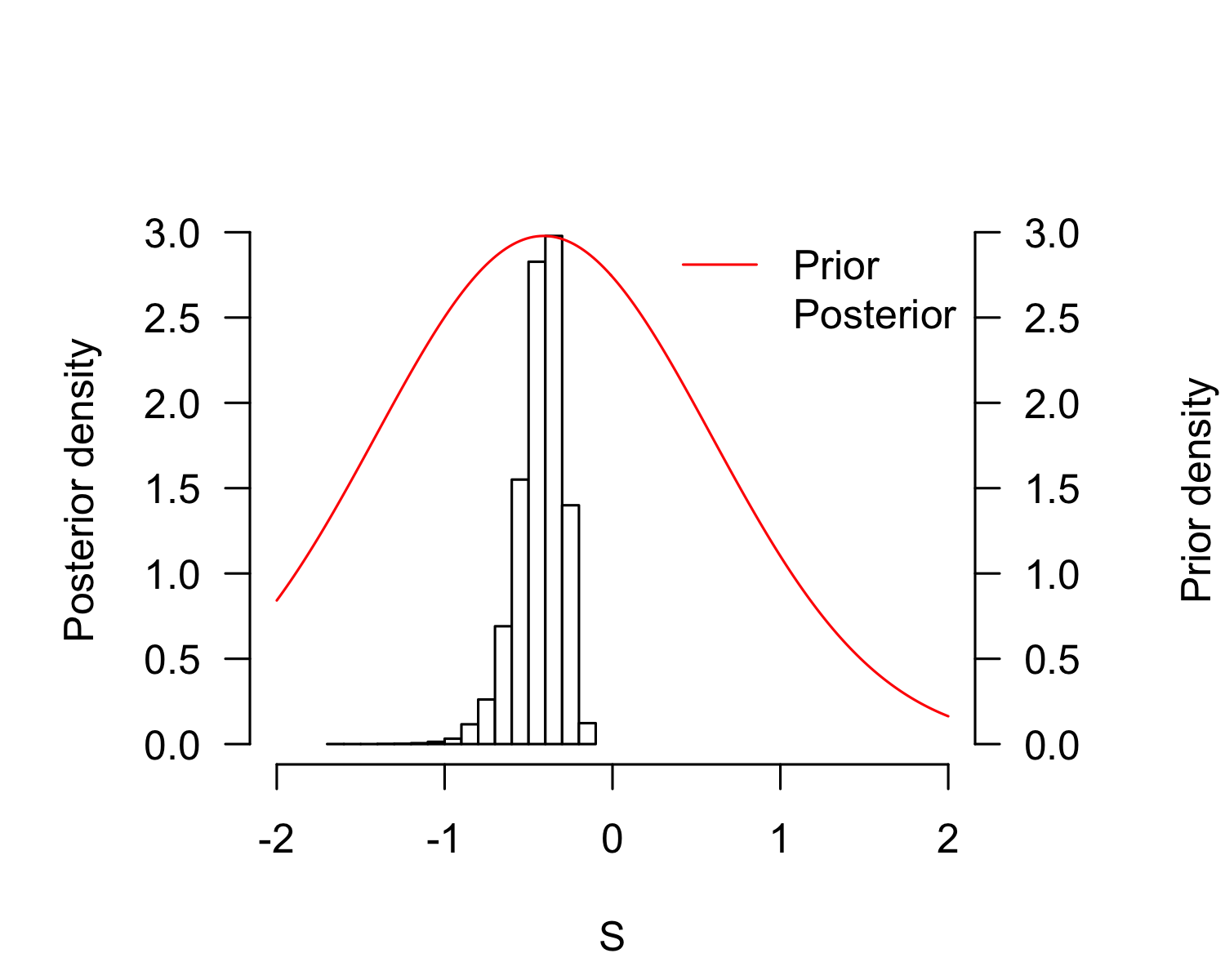


plot(result_mcmc_tsd_LoAtlanticWest, parameters = "P", xlim=c(25, 35))


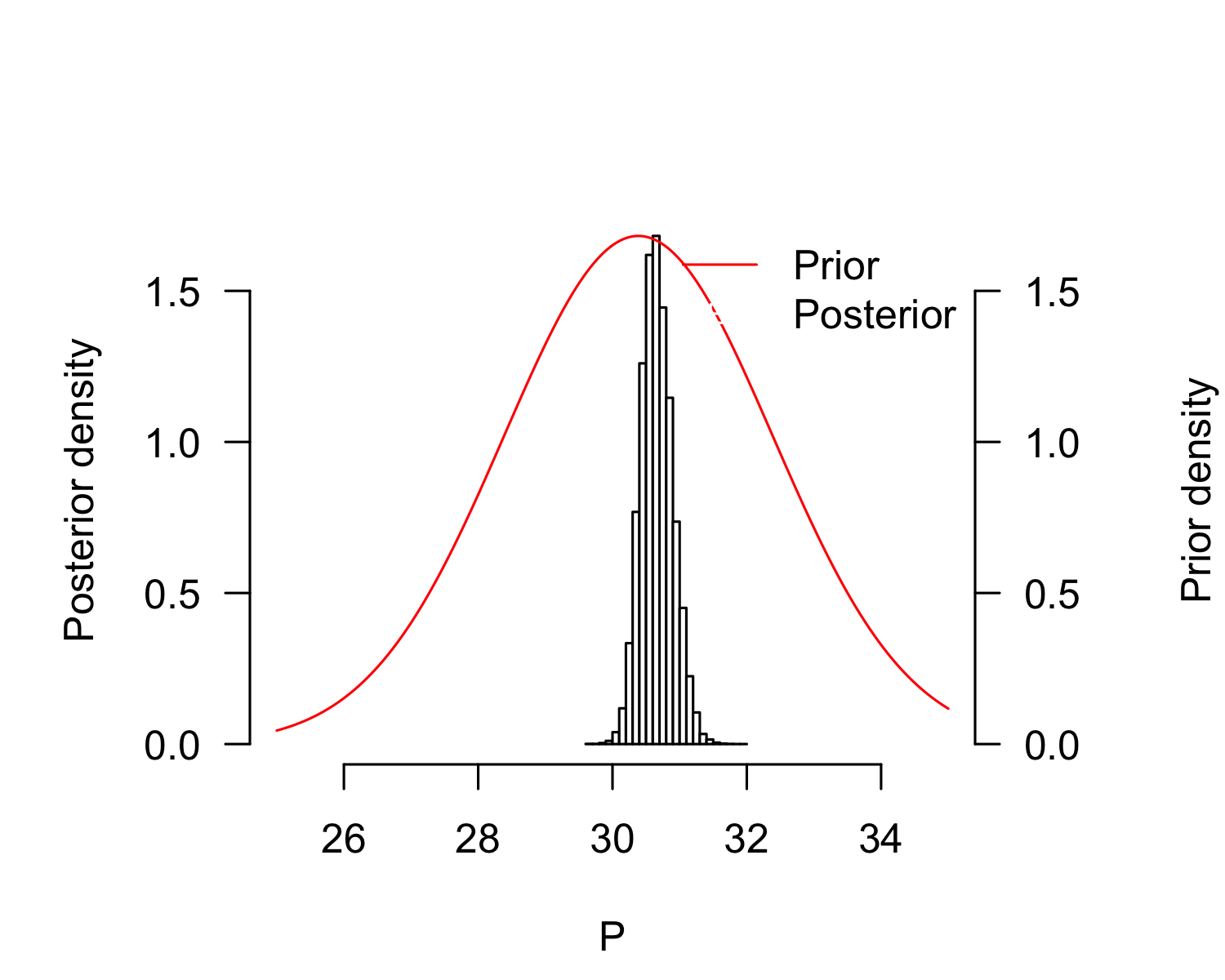


P_TRT_result_mcmc_tsd_LoAtlanticWest <- P_TRT(resultmcmc = result_mcmc_tsd_LoAtlanticWest,
 equation = "logistic", replicate.CI = 100000)
P_TRT_result_mcmc_tsd_LoAtlanticWest$P_TRT_quantiles

## lower.limit.TRT higher.limit.TRT TRT PT
## 2.5% 28.40124 31.08940 1.298434 30.23435
## 50% 29.44336 31.89074 2.442443 30.65236
## 97.5% 30.04233 33.08735 4.464554 31.14855

layout(matrix(1:3, ncol=3))
plot(result_mcmc_tsd_LoAtlanticWest, parameters = "P", xlim=c(25, 35), legend = FALSE)
text(x=ScalePreviousPlot()$xlim["begin"]+ScalePreviousPlot()$xlim["range"]*0.1,
 y=ScalePreviousPlot()$ylim["end"]*0.95, labels="A", cex=2)
plot(result_mcmc_tsd_LoAtlanticWest, parameters = "S", xlim=c(-3, 2), legend = FALSE)
text(x=ScalePreviousPlot()$xlim["begin"]+ScalePreviousPlot()$xlim["range"]*0.1,
 y=ScalePreviousPlot()$ylim["end"]*0.95, labels="B", cex=2)
rd <- sample(1:100000, 10000)
plot(as.numeric(result_mcmc_tsd_LoAtlanticWest$resultMCMC[[1]][rd, "P"]),
 as.numeric(result_mcmc_tsd_LoAtlanticWest$resultMCMC[[1]][rd, "S"]), pch=".",
 xlab="P", ylab="S", bty="n", las=1)
par(xpd=TRUE)
text(x=ScalePreviousPlot()$xlim["begin"]+ScalePreviousPlot()$xlim["range"]*0.1,
 y=ScalePreviousPlot()$ylim["begin"]+ScalePreviousPlot()$ylim["range"]*0.95, labels="C", cex=2)


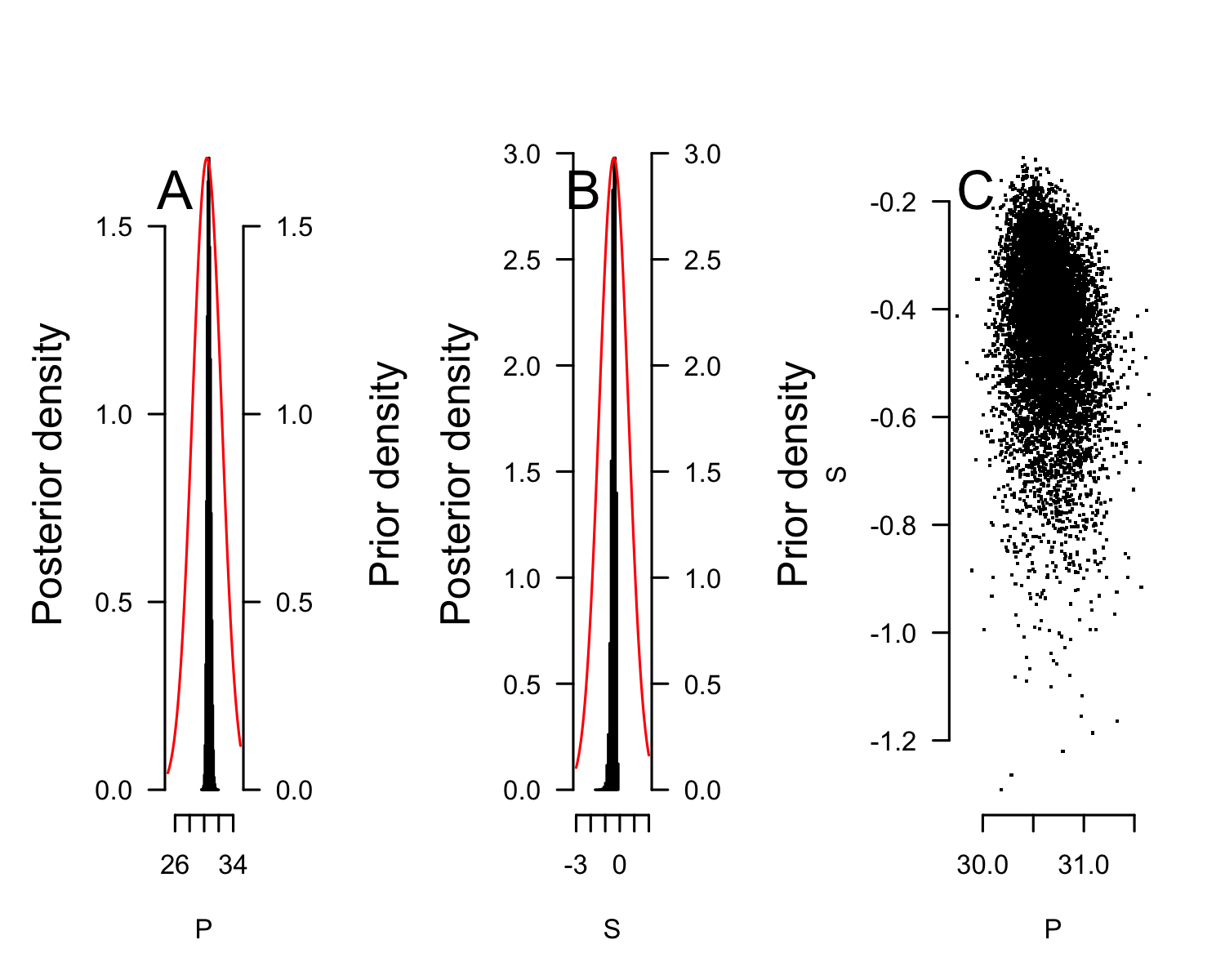


## East Pacific

pMCMC_LoPacificE <- tsd_MHmcmc_p(tsdL_Lo_PacificE_logistic, accept=TRUE)
pMCMC_LoPacificE[, "Prior1"] <- tsdL_Lo_Global_logistic$par


result_mcmc_tsd_PacificE <- tsd_MHmcmc(result=tsdL_Lo_PacificE_logistic,
 parametersMCMC=pMCMC_LoPacificE, n.iter=100000,
 n.chains = 1,
 n.adapt = 0, thin=1, trace=FALSE, adaptive = TRUE)

## Density Prior1 Prior2 SDProp Min Max Init
## P dnorm 30.3854857 2 2.0 25 35 30.4750791
## S dnorm -0.4102641 1 0.5 -2 2 -0.3853248
## Chain 1
## Best likelihood for:
## P = 30.4750791014895
## S = -0.385324829239729

plot(tsdL_Lo_PacificE_logistic, resultmcmc = result_mcmc_tsd_PacificE)


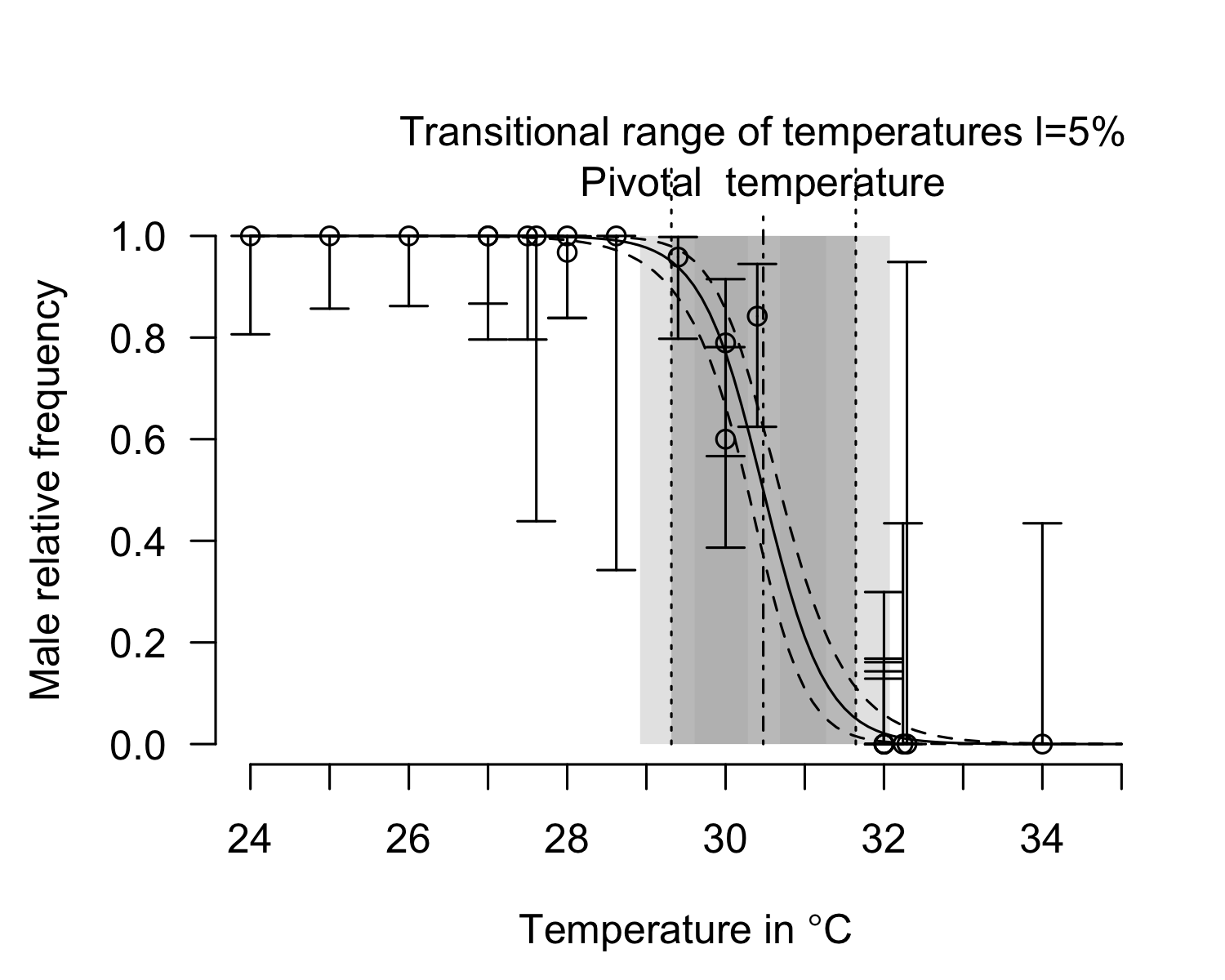


P_TRT_result_mcmc_tsd_LoPacificE <- P_TRT(resultmcmc = result_mcmc_tsd_PacificE,
 equation = "logistic",
 replicate.CI = 100000)
P_TRT_result_mcmc_tsd_LoPacificE$P_TRT_quantiles

## lower.limit.TRT higher.limit.TRT TRT PT
## 2.5% 28.93365 31.26839 1.764278 30.28095
## 50% 29.31656 31.64509 2.329158 30.47636
## 97.5% 29.61364 32.06524 3.020205 30.68458

# pdf(file = "Figure 3.pdf", width=10, height = 4, pointsize = 12)
layout(matrix(1:3, ncol=3))
plot(result_mcmc_tsd_PacificE, parameters = "P", xlim=c(25, 35), legend = FALSE)
text(x=ScalePreviousPlot()$xlim["begin"]+ScalePreviousPlot()$xlim["range"]*0.1,
 y=ScalePreviousPlot()$ylim["end"]*0.95, labels="A", cex=2)
plot(result_mcmc_tsd_PacificE, parameters = "S", xlim=c(-3, 2), ylim=c(0, 8), legend = FALSE)
text(x=ScalePreviousPlot()$xlim["begin"]+ScalePreviousPlot()$xlim["range"]*0.1,
 y=ScalePreviousPlot()$ylim["end"]*0.95, labels="B", cex=2)
rd <- sample(1:100000, 10000)
plot(as.numeric(result_mcmc_tsd_PacificE$resultMCMC[[1]][rd, "P"]),
 as.numeric(result_mcmc_tsd_PacificE$resultMCMC[[1]][rd, "S"]), pch=".",
 xlab="P", ylab="S", bty="n", las=1)
par(xpd=TRUE)
text(x=ScalePreviousPlot()$xlim["begin"]+ScalePreviousPlot()$xlim["range"]*0.1,
 y=ScalePreviousPlot()$ylim["begin"]+ScalePreviousPlot()$ylim["range"]*0.95, labels="C", cex=2)


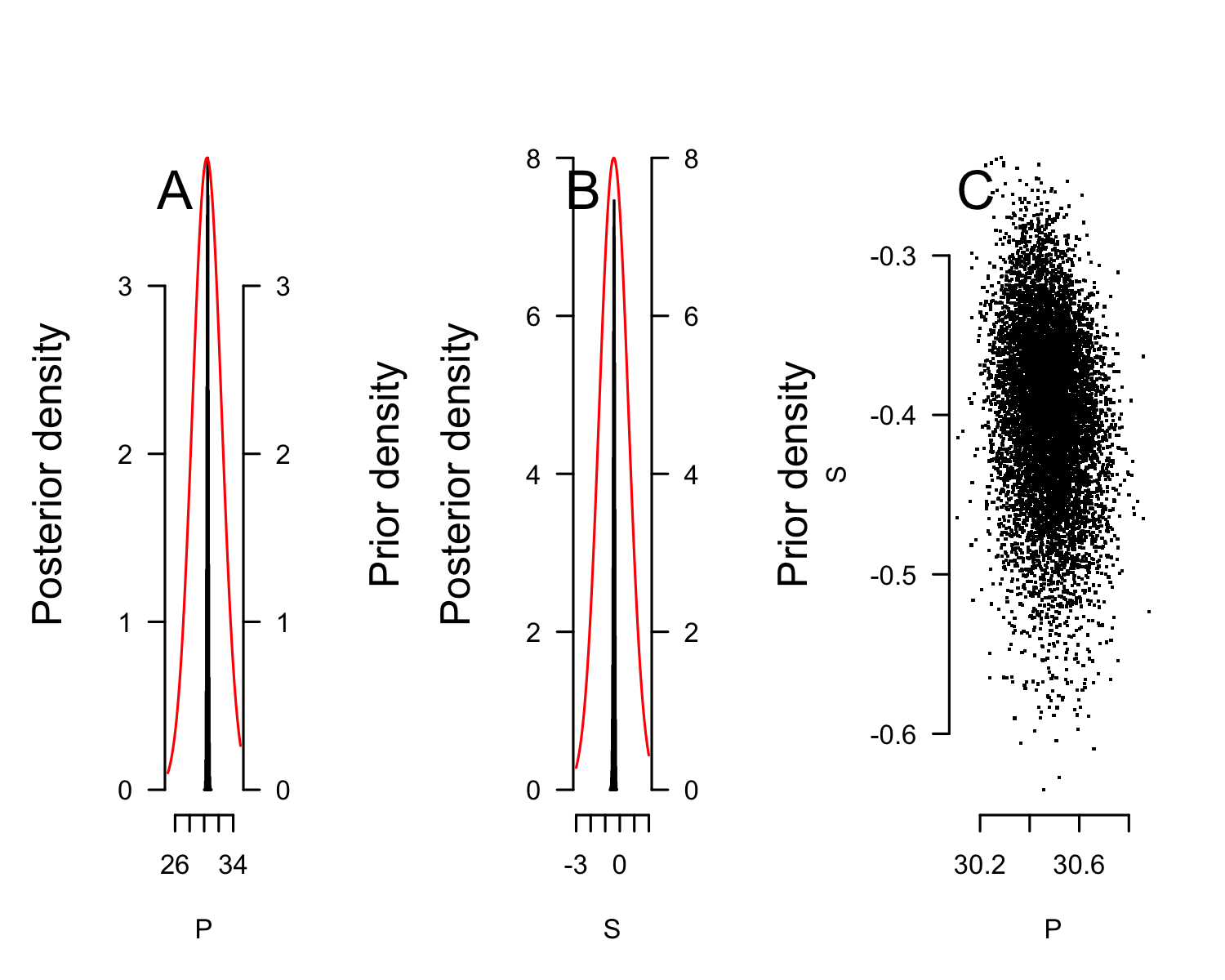


# dev.off()

## Plot of all series

pdf(file = "Figure 5.pdf", width=10, height = 14, pointsize = 14)
layout(mat = matrix(1:6, ncol=2, byrow = FALSE))
plot(tsdL_Lo_PacificE_logistic, resultmcmc = result_mcmc_tsd_PacificE, xlim=c(24, 34))
text(x=ScalePreviousPlot()$xlim["begin"]+ScalePreviousPlot()$xlim["range"]*0.02,
 y=ScalePreviousPlot()$ylim["begin"]+ScalePreviousPlot()$ylim["range"]*0.1, labels="East Pacific", cex=2, pos=4)
plot(tsdL_Lo_PacificE_Mexico_logistic, resultmcmc = result_mcmc_tsd_LoPacificE_Mexico, xlim=c(24, 34))
text(x=ScalePreviousPlot()$xlim["begin"]+ScalePreviousPlot()$xlim["range"]*0.02,
 y=ScalePreviousPlot()$ylim["begin"]+ScalePreviousPlot()$ylim["range"]*0.1, labels="Mexico", cex=2, pos=4)

plot(tsdL_Lo_PacificE_CostaRica_logistic, resultmcmc = result_mcmc_tsd_LoPacificE_CostaRica, xlim=c(24, 34))
text(x=ScalePreviousPlot()$xlim["begin"]+ScalePreviousPlot()$xlim["range"]*0.02,
 y=ScalePreviousPlot()$ylim["begin"]+ScalePreviousPlot()$ylim["range"]*0.1, labels="Costa Rica", cex=2, pos=4)

plot(tsdL_Lo_AtlanticWest_logistic, resultmcmc = result_mcmc_tsd_LoAtlanticWest, xlim=c(24, 34))
text(x=ScalePreviousPlot()$xlim["begin"]+ScalePreviousPlot()$xlim["range"]*0.02,
 y=ScalePreviousPlot()$ylim["begin"]+ScalePreviousPlot()$ylim["range"]*0.1, labels="West Atlantic", cex=2, pos=4)

plot(x=tsdL_Lo_IndianNE_logistic, resultmcmc = result_mcmc_tsd_LoIndianNE, xlim=c(24, 34))
text(x=ScalePreviousPlot()$xlim["begin"]+ScalePreviousPlot()$xlim["range"]*0.02,
 y=ScalePreviousPlot()$ylim["begin"]+ScalePreviousPlot()$ylim["range"]*0.1, labels=" Northeast Indian", cex=2, pos=4)
dev.off()

# TRT and P relationship for 4 locations from Bayesian analysis with logistic model

Costa Rica and Mexico (East Pacific RMU) and India (Northeast Indian RMU) cannot be differentiated.

Brazil (West Atlantic RMU) is different from all others.

# pdf(file = "Figure 6.pdf", width = 7, height = 7, pointsize = 14)
layout(1)
par(mar=c(4, 4, 1, 1))
rd <- runif(20000, min=1, max = 100001)
plot(x = P_TRT_result_mcmc_tsd_LoPacificE_Mexico$P_TRT[rd, "PT"],
 y = P_TRT_result_mcmc_tsd_LoPacificE_Mexico$P_TRT[rd, "TRT"],
 pch=".", xlim=c(27, 33), ylim=c(0, 8), xlab="Pivotal temperature", ylab="Transitional range of temperatures 5%", bty="n", las=1, col=gray.colors(4)[4])
rd <- runif(20000, min=1, max = 100001)
points(x = P_TRT_result_mcmc_tsd_LoIndianNE$P_TRT[rd, "PT"],
 y = P_TRT_result_mcmc_tsd_LoIndianNE$P_TRT[rd, "TRT"],
 pch=".", col=gray.colors(4)[2])
rd <- runif(5000, min=1, max = 100001)
points(x = P_TRT_result_mcmc_tsd_LoAtlanticWest$P_TRT[rd, "PT"],
 y = P_TRT_result_mcmc_tsd_LoAtlanticWest$P_TRT[rd, "TRT"],
 pch=".", col=gray.colors(4)[1])
rd <- runif(5000, min=1, max = 100001)
points(x = P_TRT_result_mcmc_tsd_LoPacificE_CostaRica$P_TRT[rd, "PT"],
 y = P_TRT_result_mcmc_tsd_LoPacificE_CostaRica$P_TRT[rd, "TRT"],
 pch=".", col=gray.colors(4)[3])
rd <- runif(1000, min=1, max = 100001)
points(x = P_TRT_result_mcmc_tsd_LoAtlanticWest$P_TRT[rd, "PT"],
 y = P_TRT_result_mcmc_tsd_LoAtlanticWest$P_TRT[rd, "TRT"],
 pch=".", col=gray.colors(4)[1])


l <- 0.80
ellipse <- dataEllipse(x=P_TRT_result_mcmc_tsd_LoPacificE_Mexico$P_TRT[rd, "PT"],
 y=P_TRT_result_mcmc_tsd_LoPacificE_Mexico$P_TRT[rd, "TRT"],
 levels=l,
 draw=FALSE)
polygon(ellipse[, 1], ellipse[, 2], border = "black", col=NULL, lwd=1)
segments(x0=30.2, x1=31,
 y0=0.4, y1=0.1)
text(x=31.1, y=0.08,
 labels = "Mexico", pos=4)


ellipse <- dataEllipse(x=P_TRT_result_mcmc_tsd_LoIndianNE$P_TRT[rd, "PT"],
 y=P_TRT_result_mcmc_tsd_LoIndianNE$P_TRT[rd, "TRT"],
 levels=l,
 draw=FALSE)
polygon(ellipse[, 1], ellipse[, 2], border ="black", col=NULL, lwd=1)
segments(x0=28.8, x1=28,
 y0=2, y1=2)
text(x=28, y=1.95,
 labels = "India", pos=2)

ellipse <- dataEllipse(x=P_TRT_result_mcmc_tsd_LoAtlanticWest$P_TRT[rd, "PT"],
 y=P_TRT_result_mcmc_tsd_LoAtlanticWest$P_TRT[rd, "TRT"],
 levels=l,
 draw=FALSE)
polygon(ellipse[, 1], ellipse[, 2], border = "black", col=NULL, lwd=1)
segments(x0=31, x1=32,
 y0=2, y1=2)
text(x=32.05, y=1.95,
 labels = "Brazil", pos=4)

ellipse <- dataEllipse(x=P_TRT_result_mcmc_tsd_LoPacificE_CostaRica$P_TRT[rd, "PT"],
 y=P_TRT_result_mcmc_tsd_LoPacificE_CostaRica$P_TRT[rd, "TRT"],
 levels=l,
 draw=FALSE)
polygon(ellipse[, 1], ellipse[, 2], border = "black", col=NULL, lwd=1)
segments(x0=30.5, x1=32,
 y0=3, y1=3)
text(x=32.05, y=2.95,
 labels = "Costa Rica", pos=4)

legend("topleft",
 legend=c("Brazil, West Atlantic", "India, Northeast Indian",
 "Costa Rica, East Pacific", "Mexico, East Pacific"),
 pch=rep(19, 4),
 col=gray.colors(4), cex=0.8)


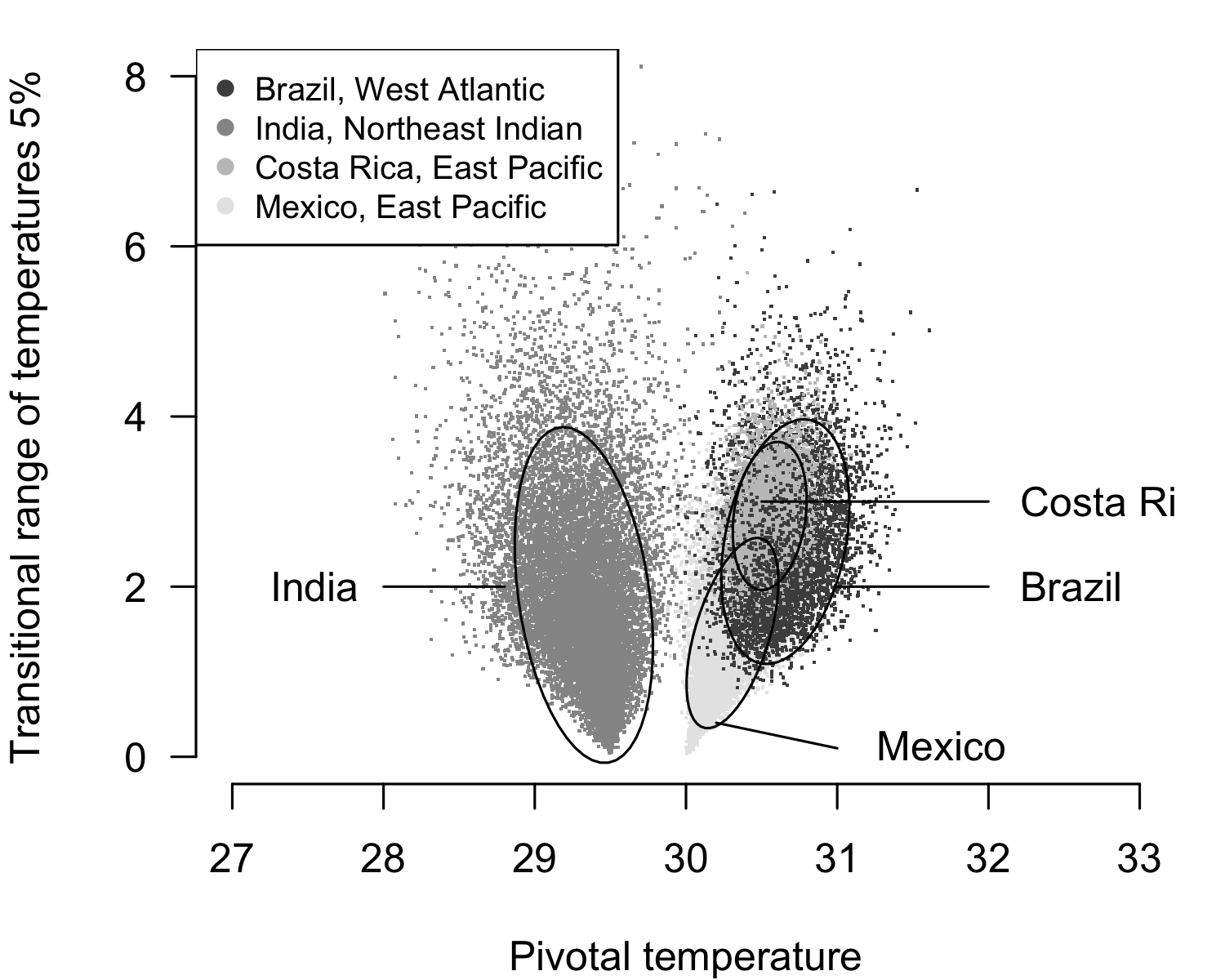


# dev.off()

# Tentative to use MCMC with flexit model

## East Pacific

tsdL_Lo_PacificE_flexit <- tsdL_Lo_Global_flexit
tsdL_Lo_PacificE_flexit$males <- Lo_PacificE$Males
tsdL_Lo_PacificE_flexit$females <- Lo_PacificE$Females
tsdL_Lo_PacificE_flexit$N <- Lo_PacificE$Males + Lo_PacificE$Females
tsdL_Lo_PacificE_flexit$temperatures <- Lo_PacificE$Incubation.temperature

pMCMC_LoPacificE <- tsd_MHmcmc_p(tsdL_Lo_PacificE_flexit, accept=TRUE)

result_mcmc_tsd_LoPacificE_flexit <- tsd_MHmcmc(result=tsdL_Lo_PacificE_flexit,
 parametersMCMC=pMCMC_LoPacificE, n.iter=100000,
 n.chains = 1,
 n.adapt = 0, thin=1, trace=FALSE, adaptive = TRUE)

## Density Prior1 Prior2 SDProp Min Max Init
## P dnorm 30.5691666 2 2.0 25.0000 35.0000 30.5691666
## S dnorm -0.7919917 1 0.5 -2.0000 2.0000 -0.7919917
## K1 dnorm -1.7179119 20 0.5 -101.7179 100.0000 -1.7179119
## K2 dnorm 200.0001724 20 0.5 -100.0000 300.0002 200.0001724
## Chain 1
## Best likelihood for:
## P = 30.9513542554092
## S = -0.340461190262008
## K1 = 1.39991097865777
## K2 = 195.754307249595

layout(1)
plot(tsdL_Lo_PacificE_flexit, resultmcmc = result_mcmc_tsd_LoPacificE_flexit)


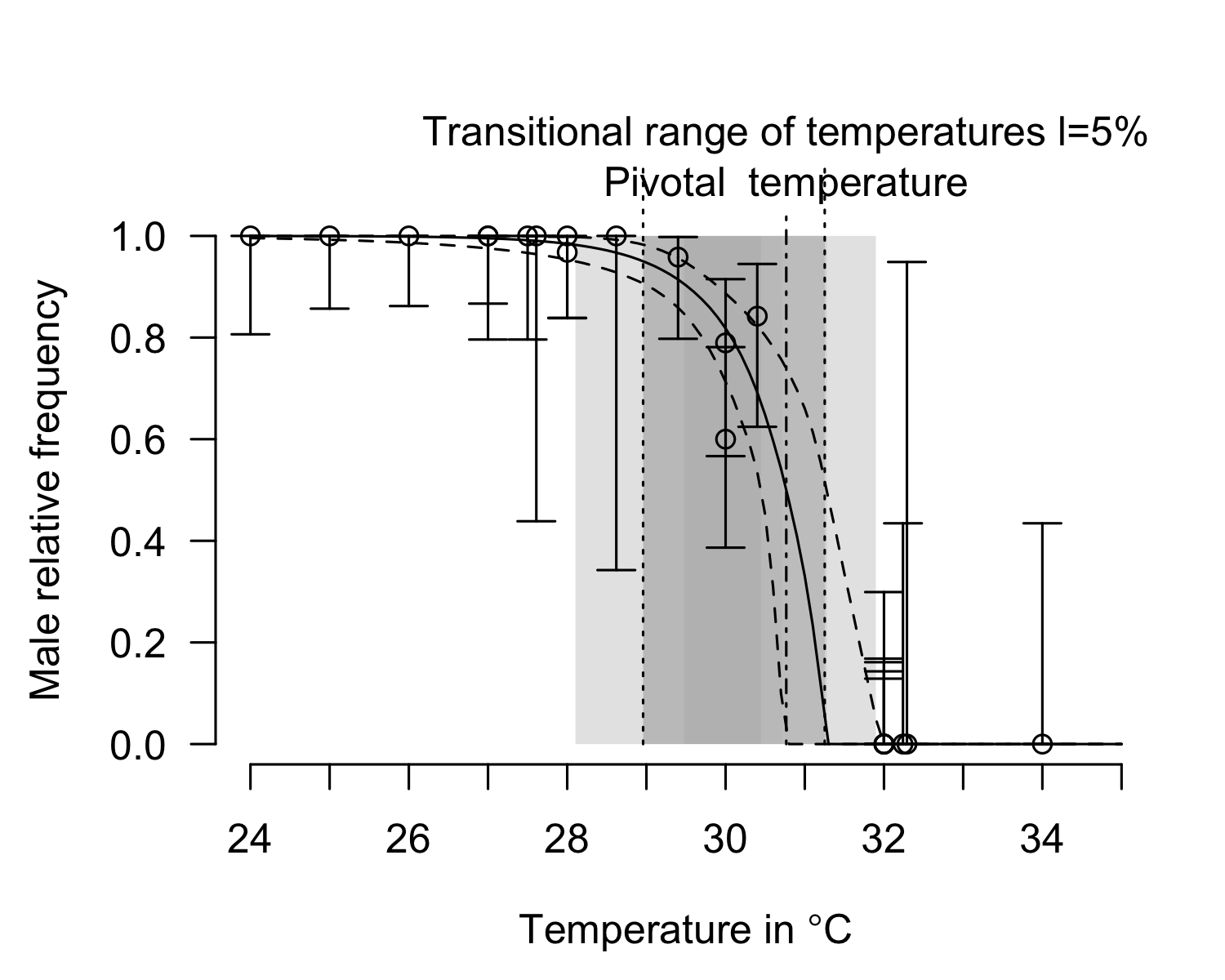


plot(result_mcmc_tsd_LoPacificE_flexit, parameters = "S", xlim=c(-2, 2))


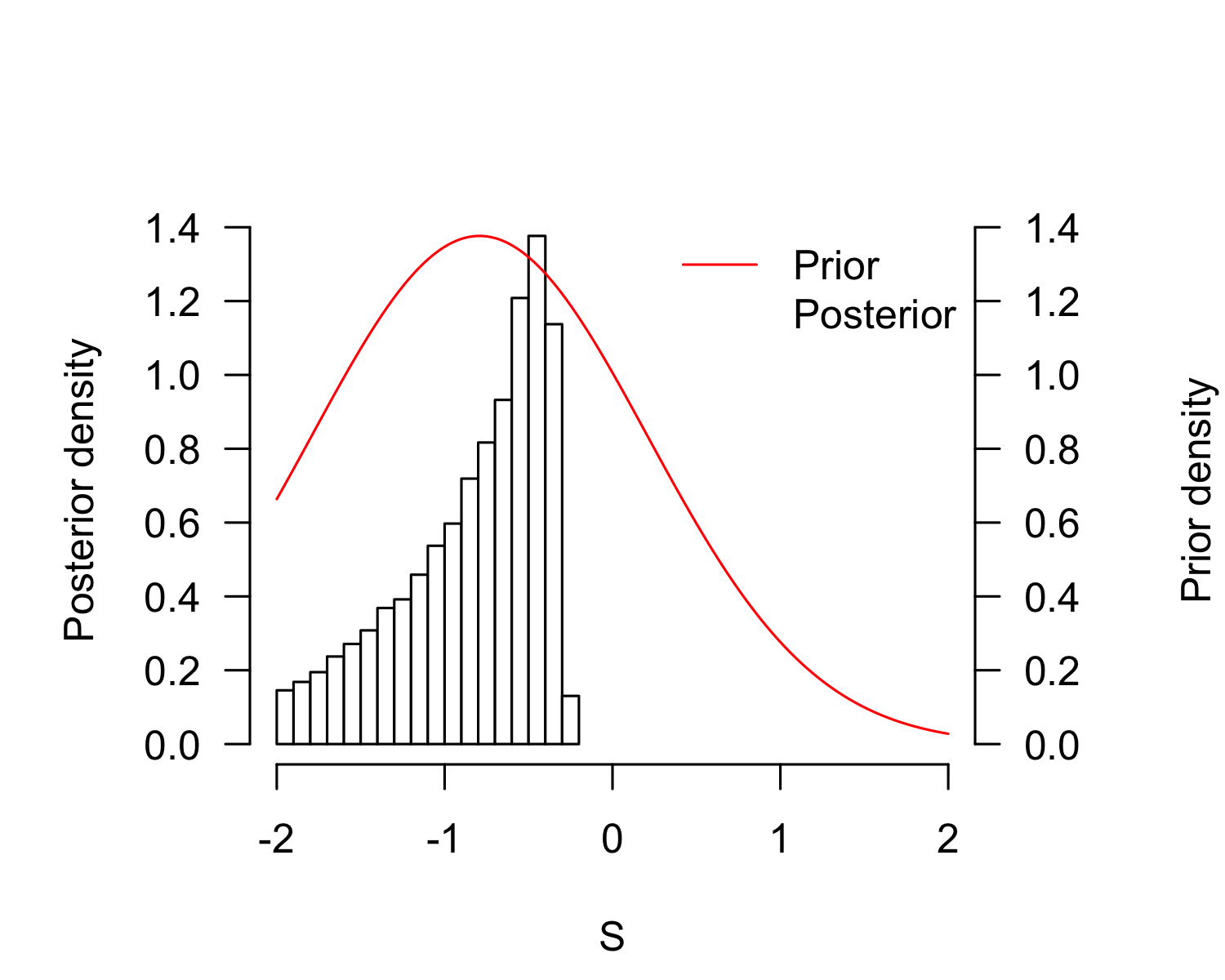


plot(result_mcmc_tsd_LoPacificE_flexit, parameters = "P", xlim=c(25, 35))


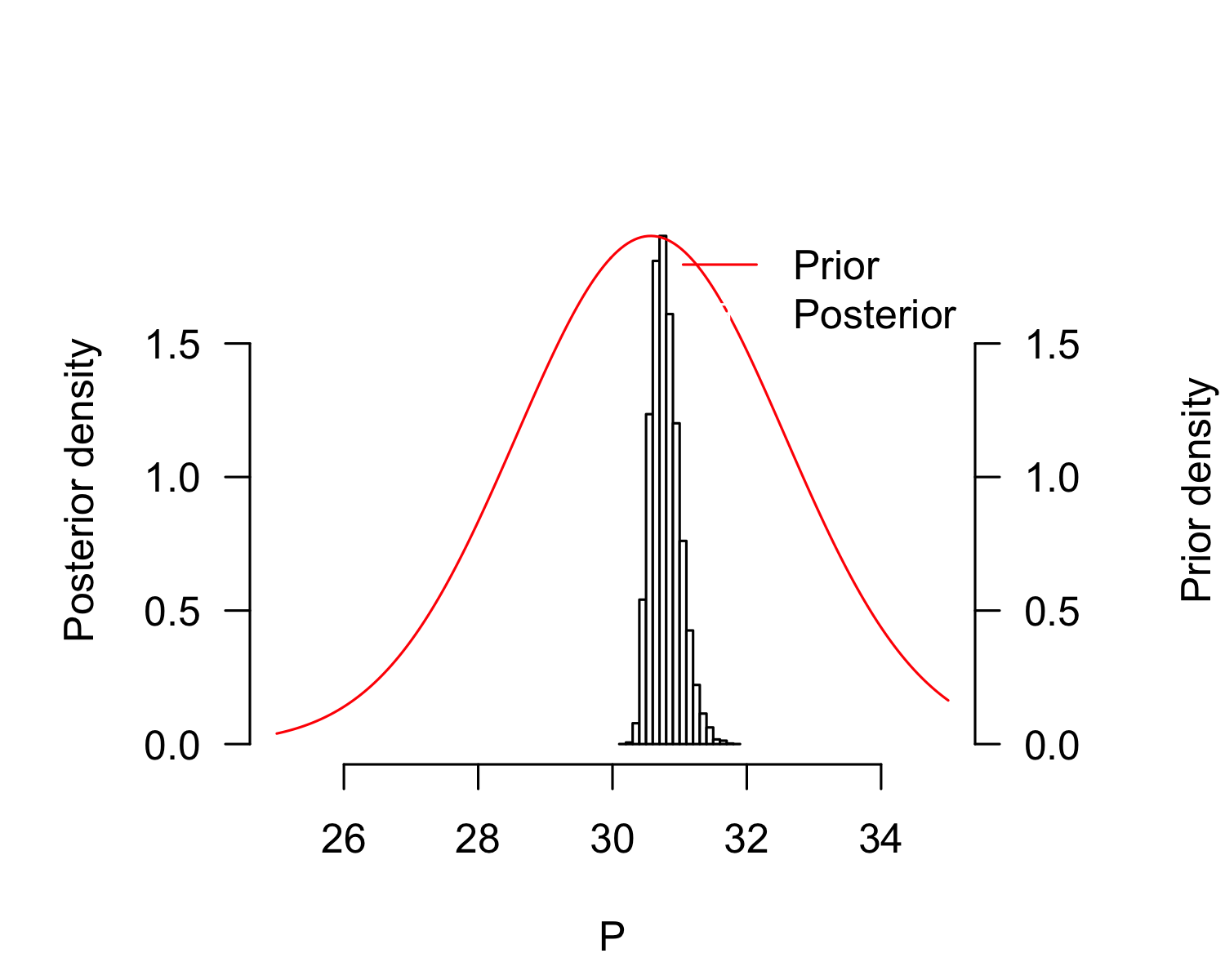


plot(result_mcmc_tsd_LoPacificE_flexit, parameters = "K1", xlim=c(-10, 10))


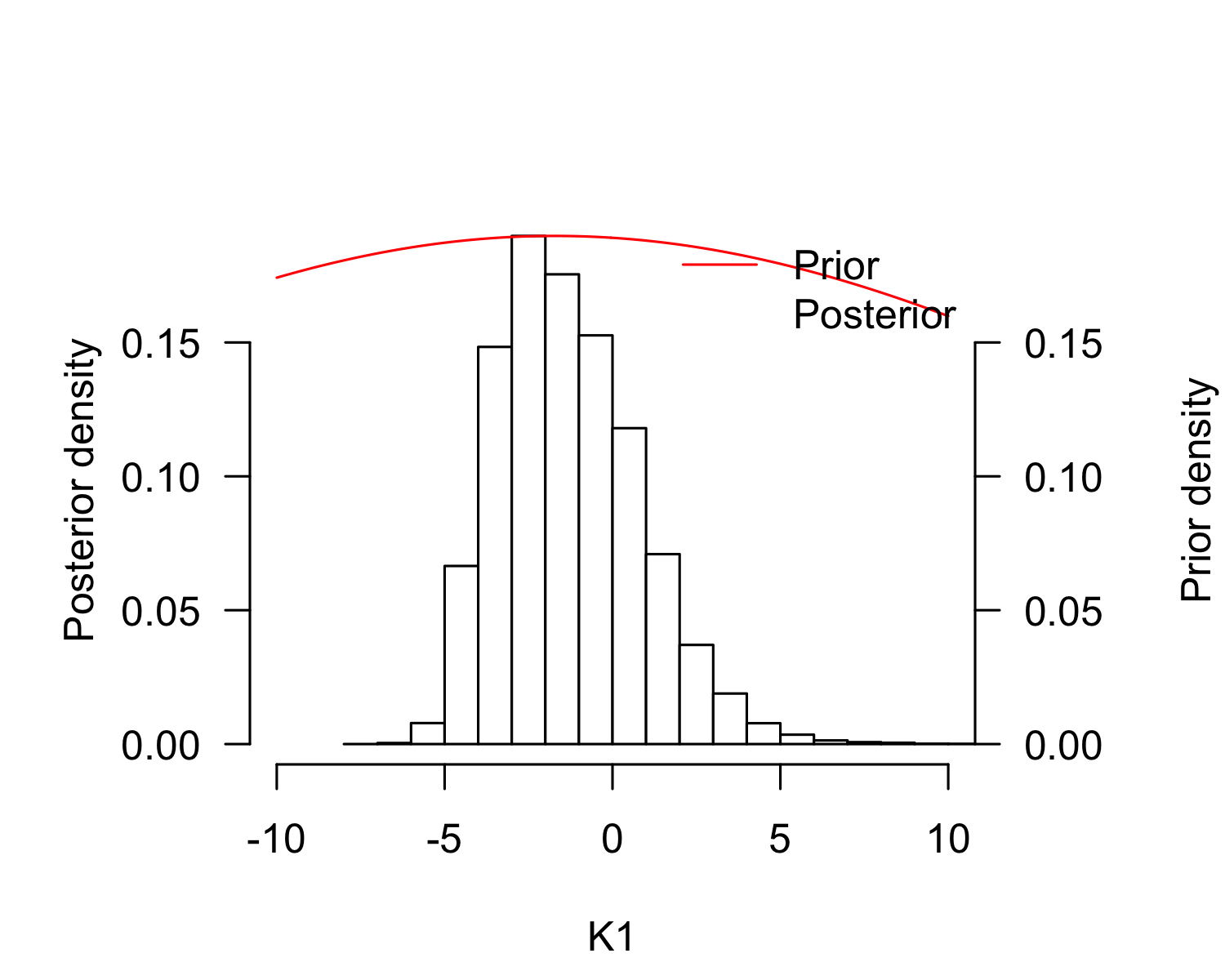


plot(result_mcmc_tsd_LoPacificE_flexit, parameters = "K2", xlim=c(150, 250))


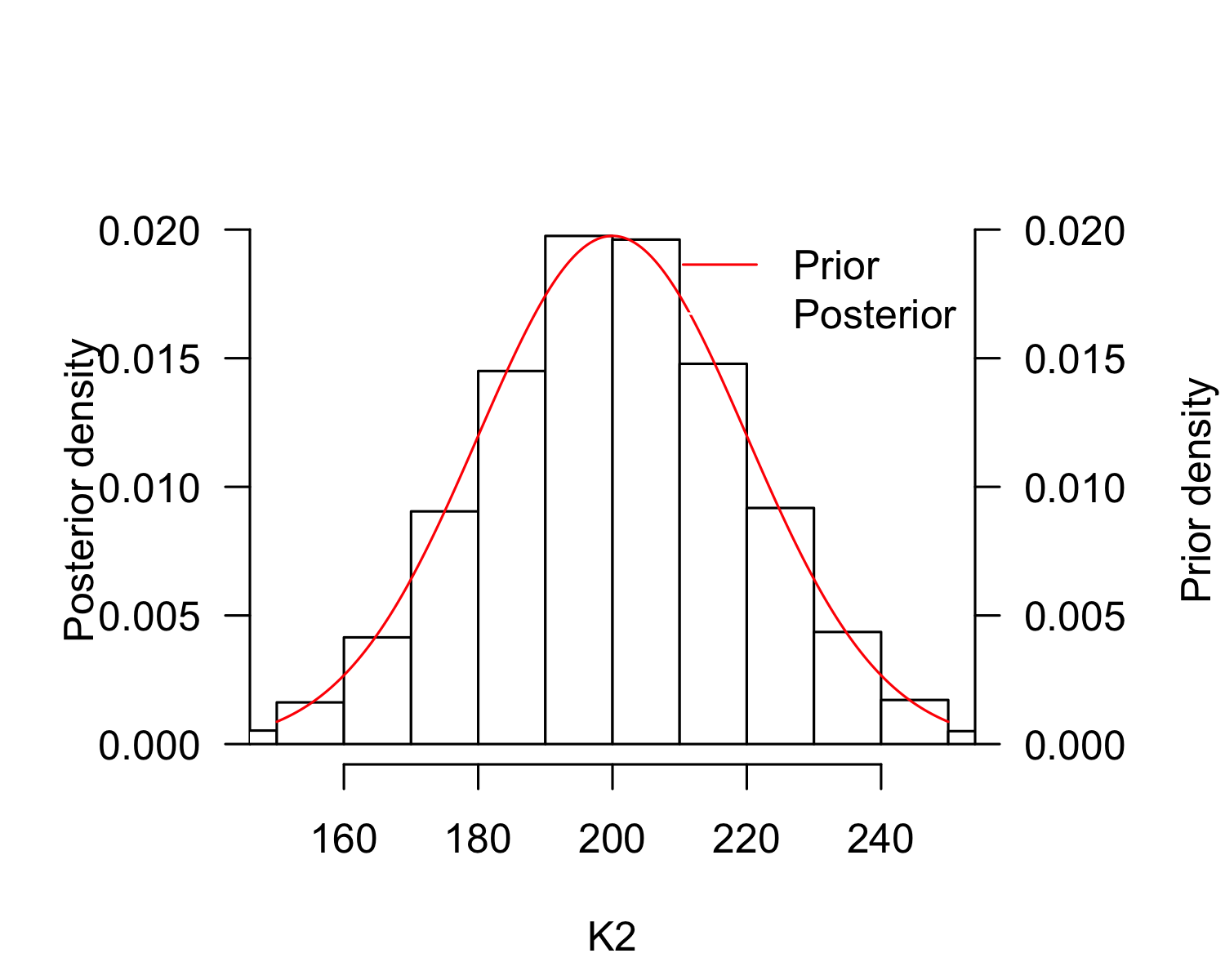


P_TRT_result_mcmc_tsd_LoPacificE_flexit <- P_TRT(resultmcmc = result_mcmc_tsd_LoPacificE_flexit,
 replicate.CI = 100000)
P_TRT_result_mcmc_tsd_LoPacificE_flexit$P_TRT_quantiles

## lower.limit.TRT higher.limit.TRT TRT PT
## 2.5% 28.10009 30.71286 1.490922 30.44826
## 50% 28.95989 31.25560 2.341591 30.76635
## 97.5% 29.46477 31.89440 3.404256 31.27724

## Costa Rica, East Pacific

tsdL_Lo_PacificE_CostaRica_flexit <- tsdL_Lo_Global_flexit
tsdL_Lo_PacificE_CostaRica_flexit$males <- Lo_PacificE_CostaRica$Males
tsdL_Lo_PacificE_CostaRica_flexit$females <- Lo_PacificE_CostaRica$Females
tsdL_Lo_PacificE_CostaRica_flexit$N <- Lo_PacificE_CostaRica$Males + Lo_PacificE_CostaRica$Females
tsdL_Lo_PacificE_CostaRica_flexit$temperatures <- Lo_PacificE_CostaRica$Incubation.temperature

pMCMC_LoPacificE_CostaRica <- tsd_MHmcmc_p(tsdL_Lo_PacificE_CostaRica_flexit, accept=TRUE)

result_mcmc_tsd_LoPacificE_CostaRica_flexit <- tsd_MHmcmc(result=tsdL_Lo_PacificE_CostaRica_flexit,
 parametersMCMC=pMCMC_LoPacificE_CostaRica, n.iter=100000,
 n.chains = 1,
 n.adapt = 0, thin=1, trace=FALSE, adaptive = TRUE)

## Density Prior1 Prior2 SDProp Min Max Init
## P dnorm 30.5691666 2 2.0 25.0000 35.0000 30.5691666
## S dnorm -0.7919917 1 0.5 -2.0000 2.0000 -0.7919917
## K1 dnorm -1.7179119 20 0.5 -101.7179 100.0000 -1.7179119
## K2 dnorm 200.0001724 20 0.5 -100.0000 300.0002 200.0001724
## Chain 1
## Best likelihood for:
## P = 31.0225610053176
## S = -0.363213933100986
## K1 = 0.319651189186973
## K2 = 203.320851417758

plot(tsdL_Lo_PacificE_CostaRica_flexit, resultmcmc = result_mcmc_tsd_LoPacificE_CostaRica_flexit)


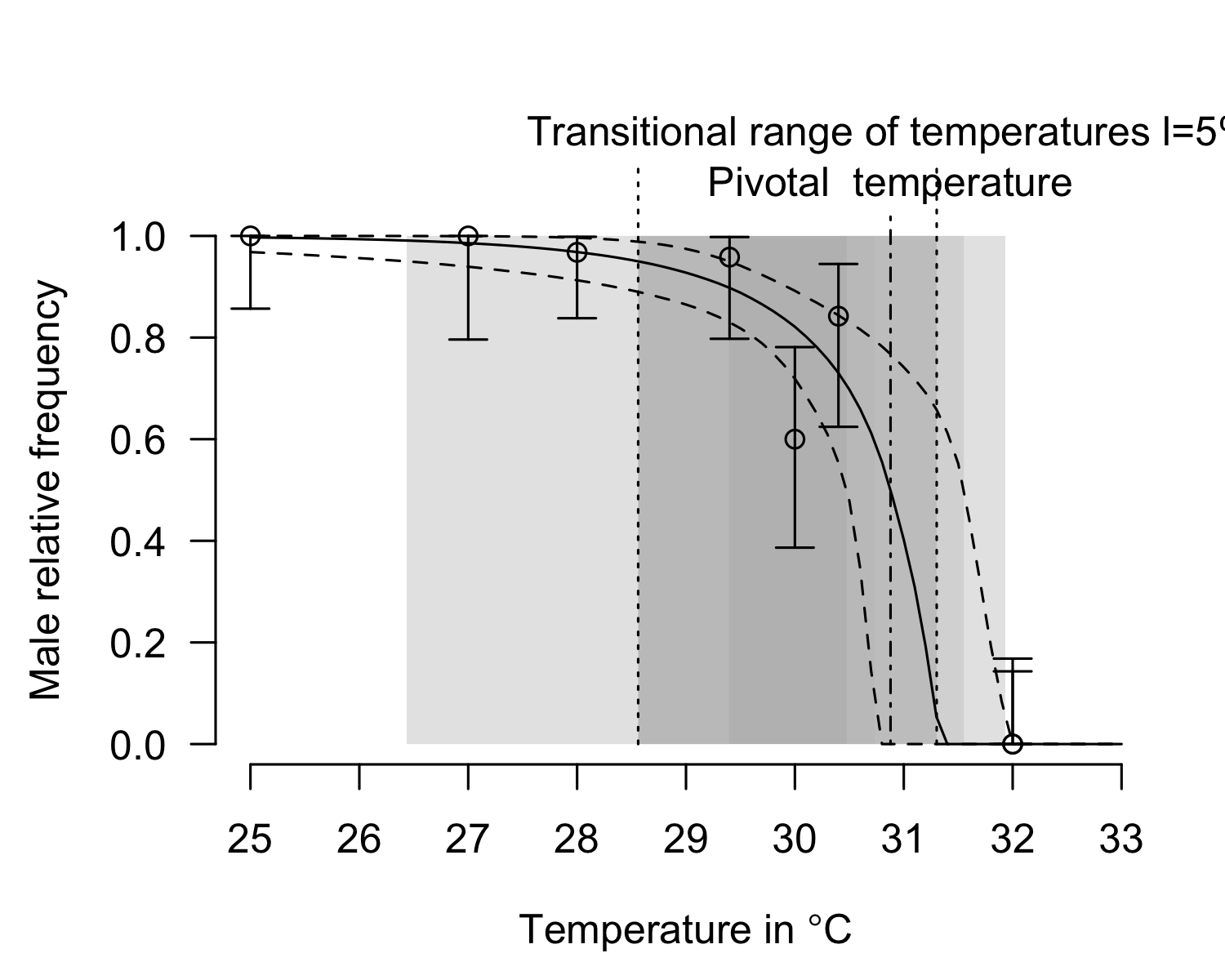


plot(result_mcmc_tsd_LoPacificE_CostaRica_flexit, parameters = "S", xlim=c(-2, 2))


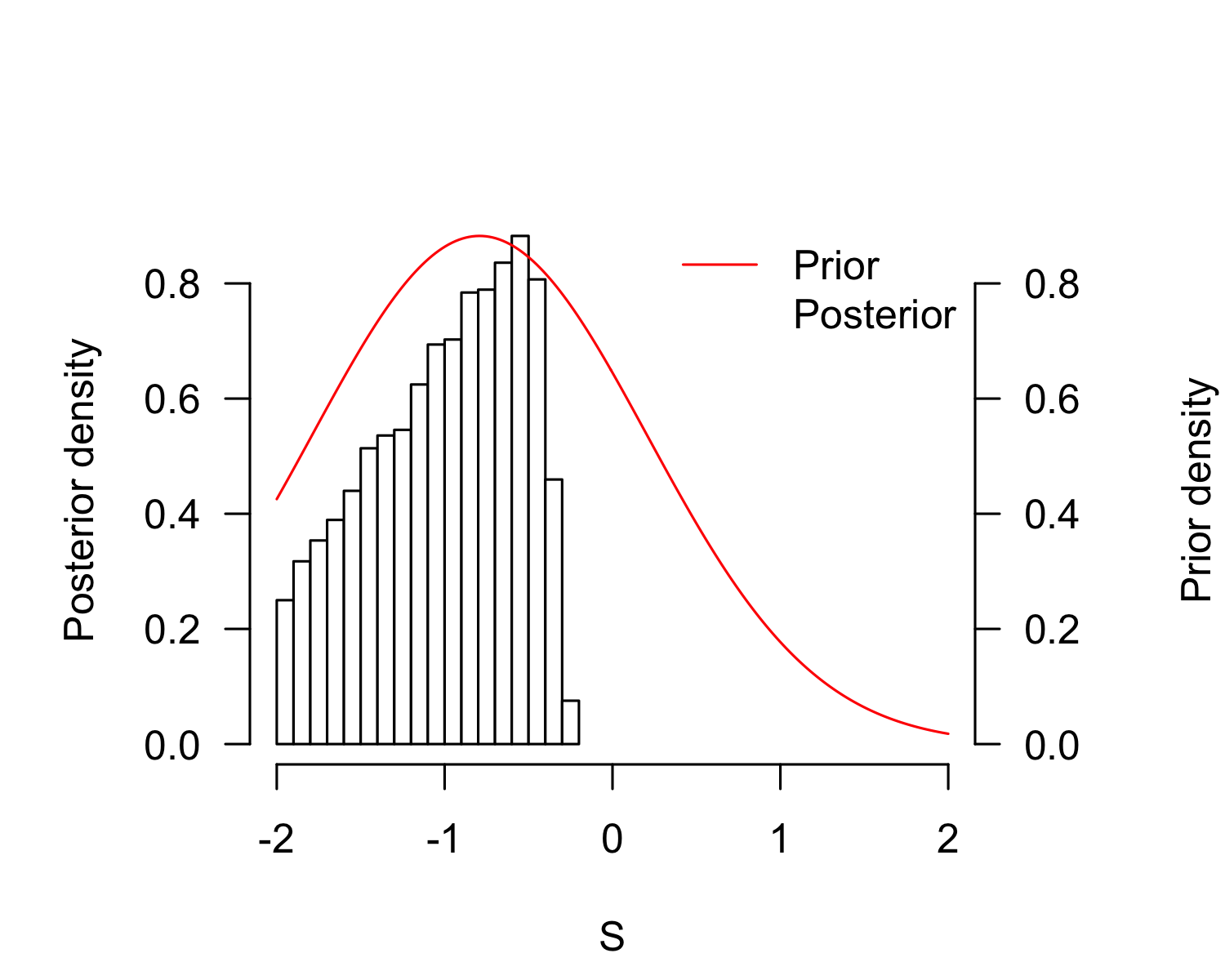


plot(result_mcmc_tsd_LoPacificE_CostaRica_flexit, parameters = "P", xlim=c(25, 35))


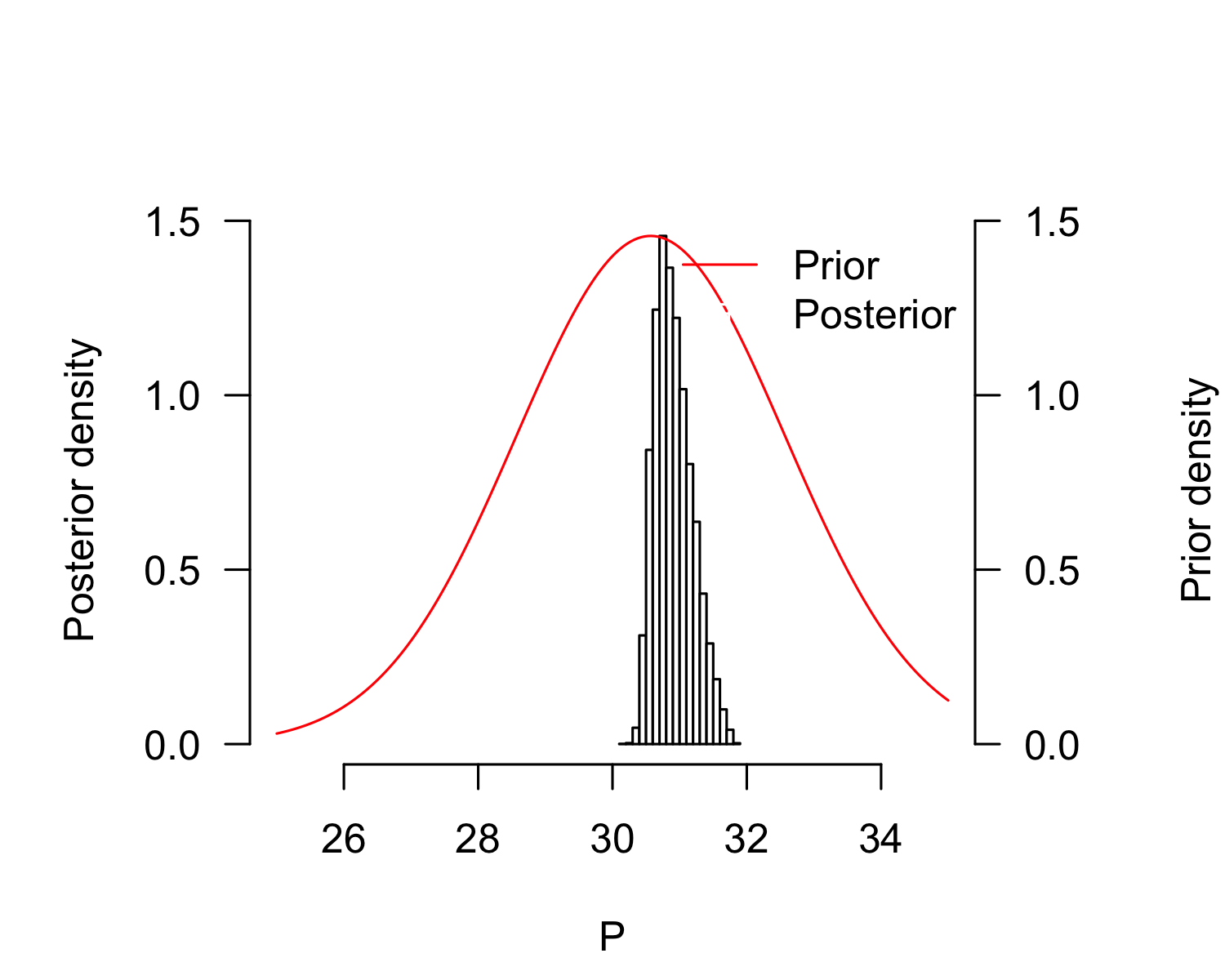


plot(result_mcmc_tsd_LoPacificE_CostaRica_flexit, parameters = "K1", xlim=c(-10, 10))


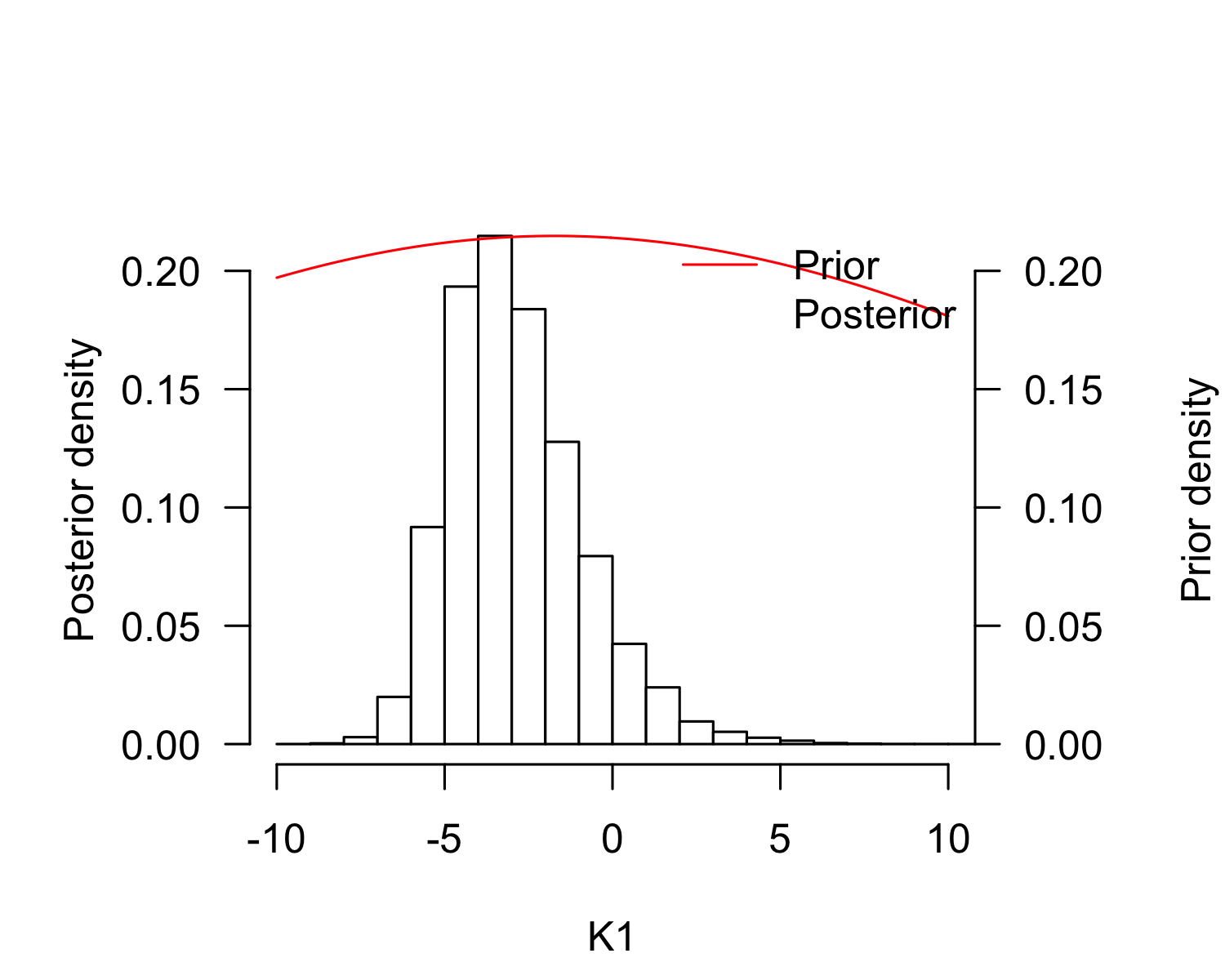


plot(result_mcmc_tsd_LoPacificE_CostaRica_flexit, parameters = "K2", xlim=c(150, 250))


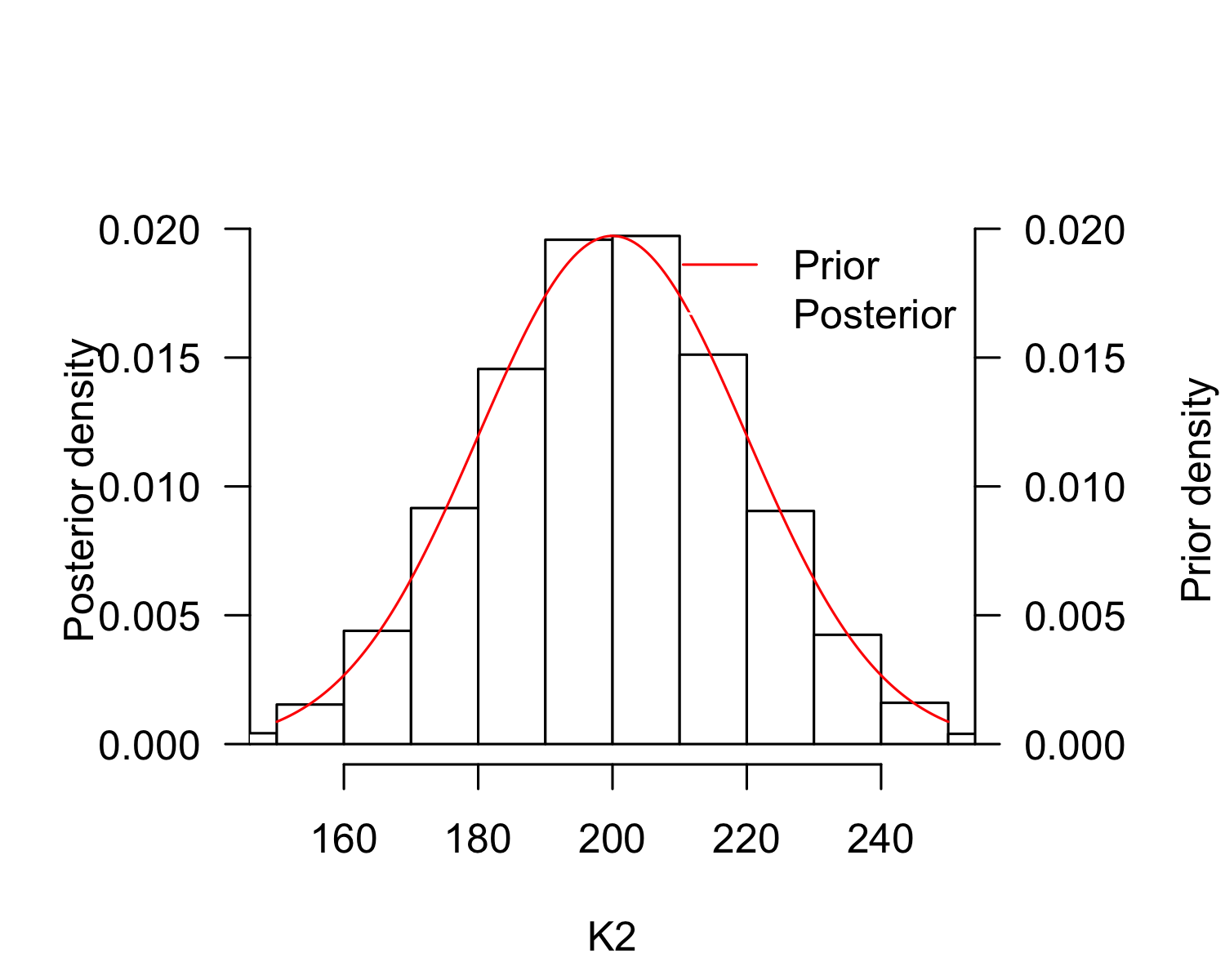


P_TRT_result_mcmc_tsd_LoPacificE_CostaRica_flexit <- P_TRT(resultmcmc = result_mcmc_tsd_LoPacificE_CostaRica_flexit,
 replicate.CI = 100000)
P_TRT_result_mcmc_tsd_LoPacificE_CostaRica_flexit$P_TRT_quantiles

## lower.limit.TRT higher.limit.TRT TRT PT
## 2.5% 26.37939 30.74322 1.631191 30.47712
## 50% 28.55880 31.29604 2.772855 30.88055
## 97.5% 29.38881 31.92885 5.133827 31.53774

## Mexico, East Pacific

tsdL_Lo_PacificE_Mexico_flexit <- tsdL_Lo_Global_flexit
tsdL_Lo_PacificE_Mexico_flexit$males <- Lo_PacificE_Mexico$Males
tsdL_Lo_PacificE_Mexico_flexit$females <- Lo_PacificE_Mexico$Females
tsdL_Lo_PacificE_Mexico_flexit$N <- Lo_PacificE_Mexico$Males + Lo_PacificE_Mexico$Females
tsdL_Lo_PacificE_Mexico_flexit$temperatures <- Lo_PacificE_Mexico$Incubation.temperature

pMCMC_LoPacificE_Mexico <- tsd_MHmcmc_p(tsdL_Lo_PacificE_Mexico_flexit, accept=TRUE)

result_mcmc_tsd_LoPacificE_Mexico_flexit <- tsd_MHmcmc(result=tsdL_Lo_PacificE_Mexico_flexit,
 parametersMCMC=pMCMC_LoPacificE_Mexico, n.iter=100000,
 n.chains = 1,
 n.adapt = 0, thin=1, trace=FALSE, adaptive = TRUE)

## Density Prior1 Prior2 SDProp Min Max Init
## P dnorm 30.5691666 2 2.0 25.0000 35.0000 30.5691666
## S dnorm -0.7919917 1 0.5 -2.0000 2.0000 -0.7919917
## K1 dnorm -1.7179119 20 0.5 -101.7179 100.0000 -1.7179119
## K2 dnorm 200.0001724 20 0.5 -100.0000 300.0002 200.0001724
## Chain 1
## Best likelihood for:
## P = 30.7351700460265
## S = -0.310682816808138
## K1 = 29.9506335477079
## K2 = 208.890825724273

plot(tsdL_Lo_PacificE_Mexico_flexit, resultmcmc = result_mcmc_tsd_LoPacificE_Mexico_flexit)


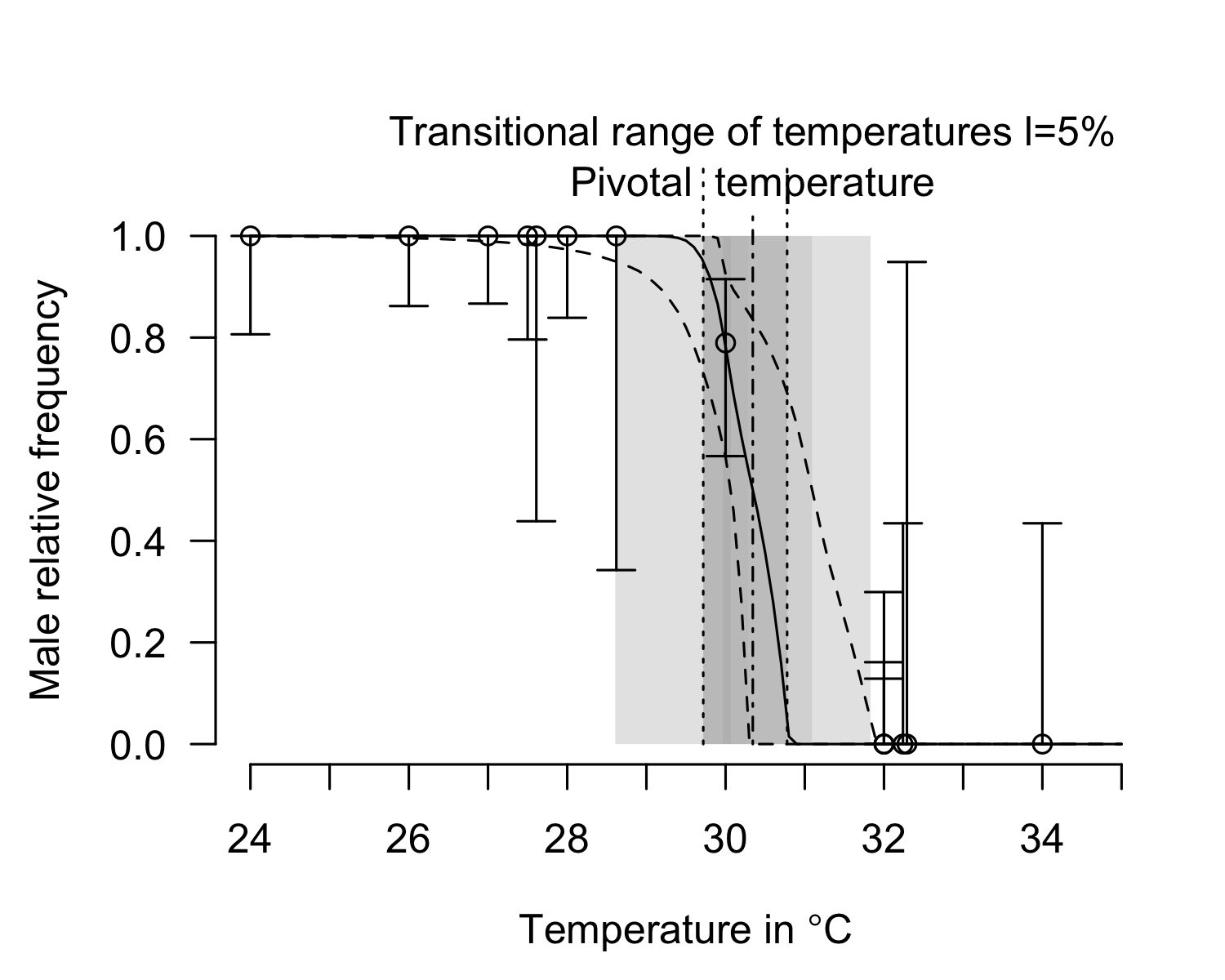


plot(result_mcmc_tsd_LoPacificE_Mexico_flexit, parameters = "S", xlim=c(-2, 2))


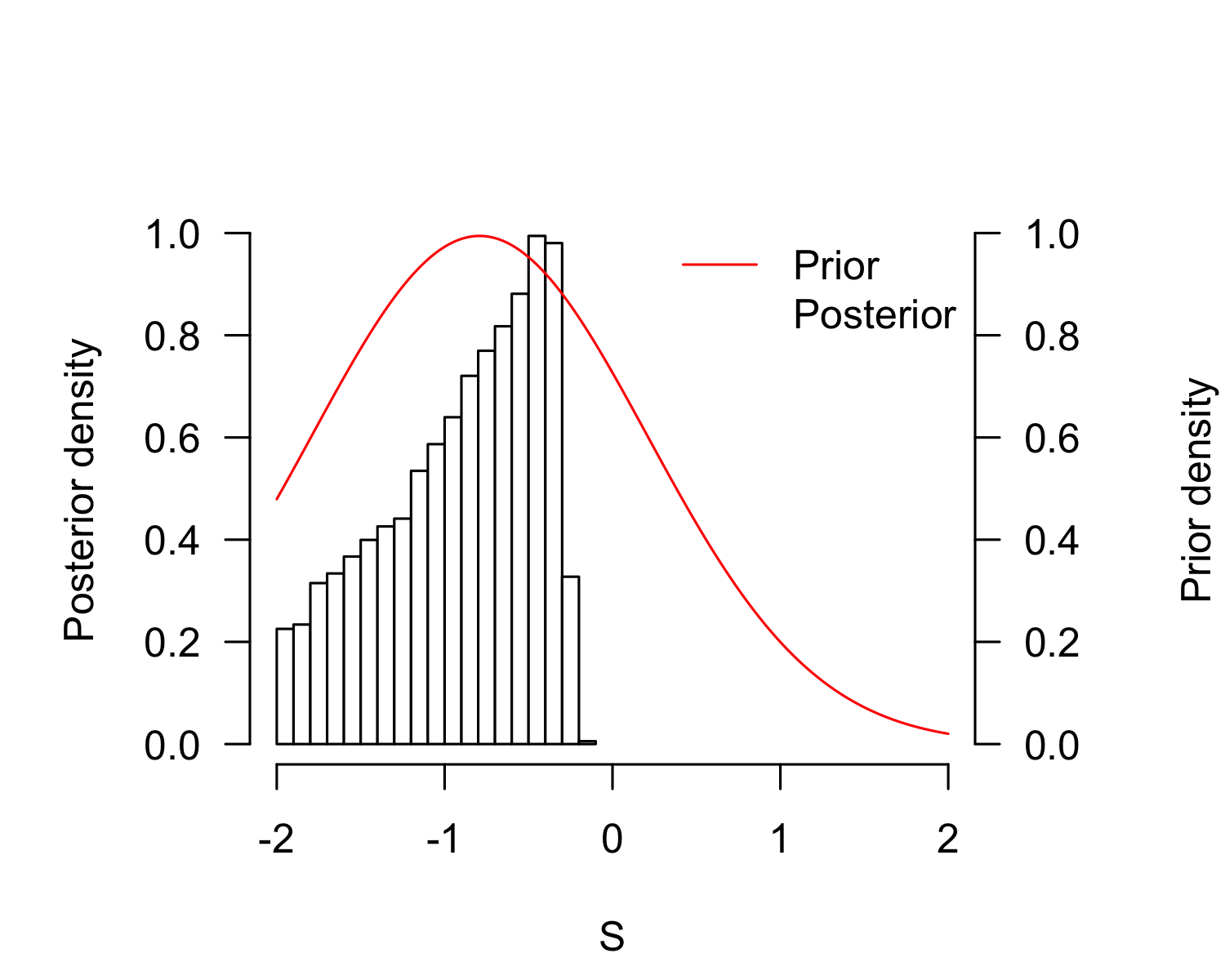


plot(result_mcmc_tsd_LoPacificE_Mexico_flexit, parameters = "P", xlim=c(25, 35))


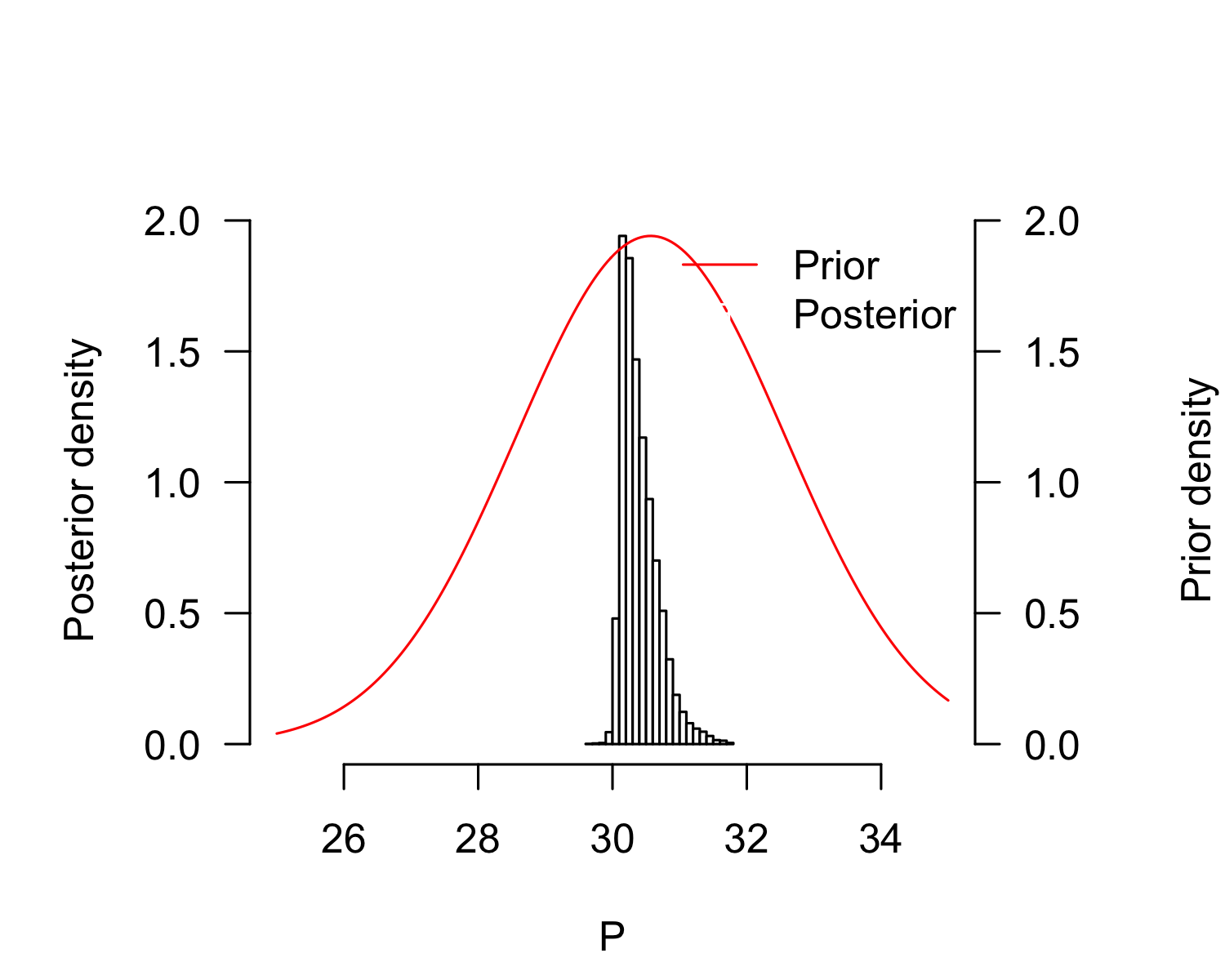


plot(result_mcmc_tsd_LoPacificE_Mexico_flexit, parameters = "K1", xlim=c(-100, 100))


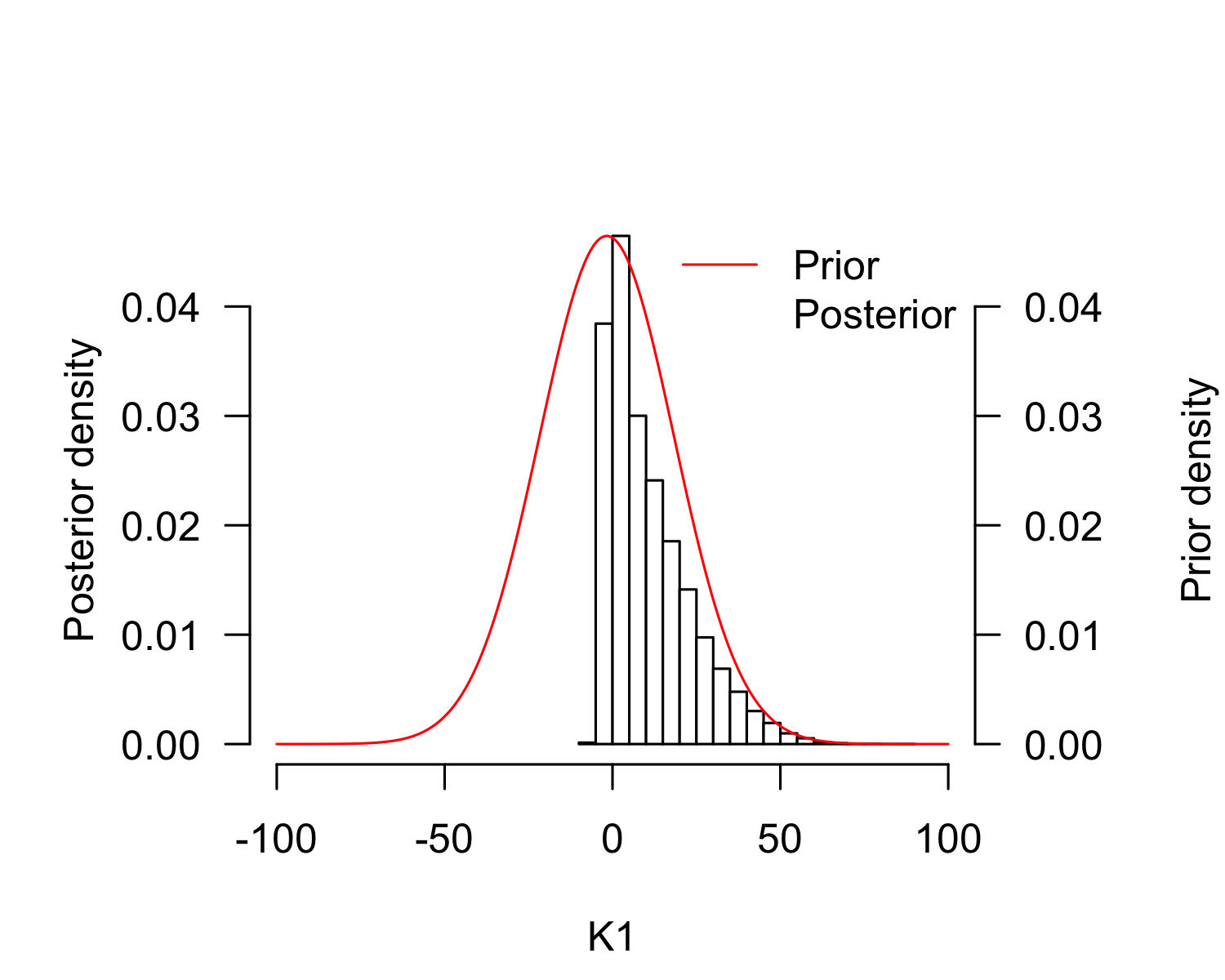


plot(result_mcmc_tsd_LoPacificE_Mexico_flexit, parameters = "K2", xlim=c(150, 250))


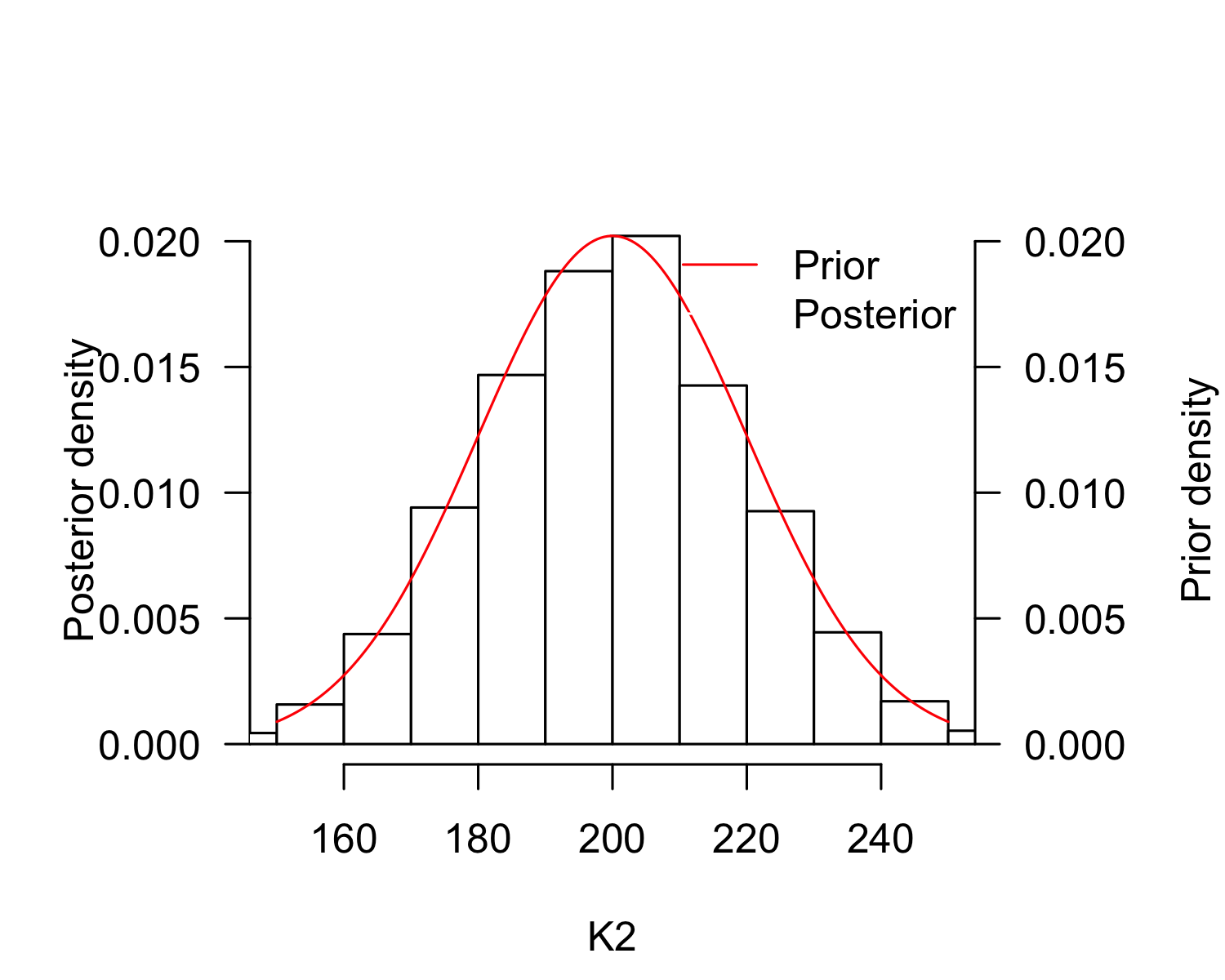


P_TRT_result_mcmc_tsd_LoPacificE_Mexico_flexit <- P_TRT(resultmcmc = result_mcmc_tsd_LoPacificE_Mexico_flexit,
 replicate.CI = 100000)
P_TRT_result_mcmc_tsd_LoPacificE_Mexico_flexit$P_TRT_quantiles

## lower.limit.TRT higher.limit.TRT TRT PT
## 2.5% 28.60570 30.28569 0.3872126 30.06975
## 50% 29.71546 30.77548 1.1585251 30.34325
## 97.5% 29.96556 31.83527 2.7631336 31.09226

## Brazil, West Atlantic

tsdL_Lo_AtlanticWest_Brazil_flexit <- tsdL_Lo_Global_flexit
tsdL_Lo_AtlanticWest_Brazil_flexit$males <- Lo_AtlanticWest$Males
tsdL_Lo_AtlanticWest_Brazil_flexit$females <- Lo_AtlanticWest$Females
tsdL_Lo_AtlanticWest_Brazil_flexit$N <- Lo_AtlanticWest$Males + Lo_AtlanticWest$Females
tsdL_Lo_AtlanticWest_Brazil_flexit$temperatures <- Lo_AtlanticWest$Incubation.temperature

pMCMC_LoAtlanticWest_Brazil <- tsd_MHmcmc_p(tsdL_Lo_AtlanticWest_Brazil_flexit, accept=TRUE)

result_mcmc_tsd_LoAtlanticWest_flexit <- tsd_MHmcmc(result=tsdL_Lo_AtlanticWest_Brazil_flexit,
 parametersMCMC=pMCMC_LoAtlanticWest_Brazil, n.iter=100000,
 n.chains = 1,
 n.adapt = 0, thin=1, trace=FALSE, adaptive = TRUE)

## Density Prior1 Prior2 SDProp Min Max Init
## P dnorm 30.5691666 2 2.0 25.0000 35.0000 30.5691666
## S dnorm -0.7919917 1 0.5 -2.0000 2.0000 -0.7919917
## K1 dnorm -1.7179119 20 0.5 -101.7179 100.0000 -1.7179119
## K2 dnorm 200.0001724 20 0.5 -100.0000 300.0002 200.0001724
## Chain 1
## Best likelihood for:
## P = 30.7325912403388
## S = -1.99274951656594
## K1 = -4.16712224436954
## K2 = 211.281253256487

plot(tsdL_Lo_AtlanticWest_Brazil_flexit, resultmcmc = result_mcmc_tsd_LoAtlanticWest_flexit)


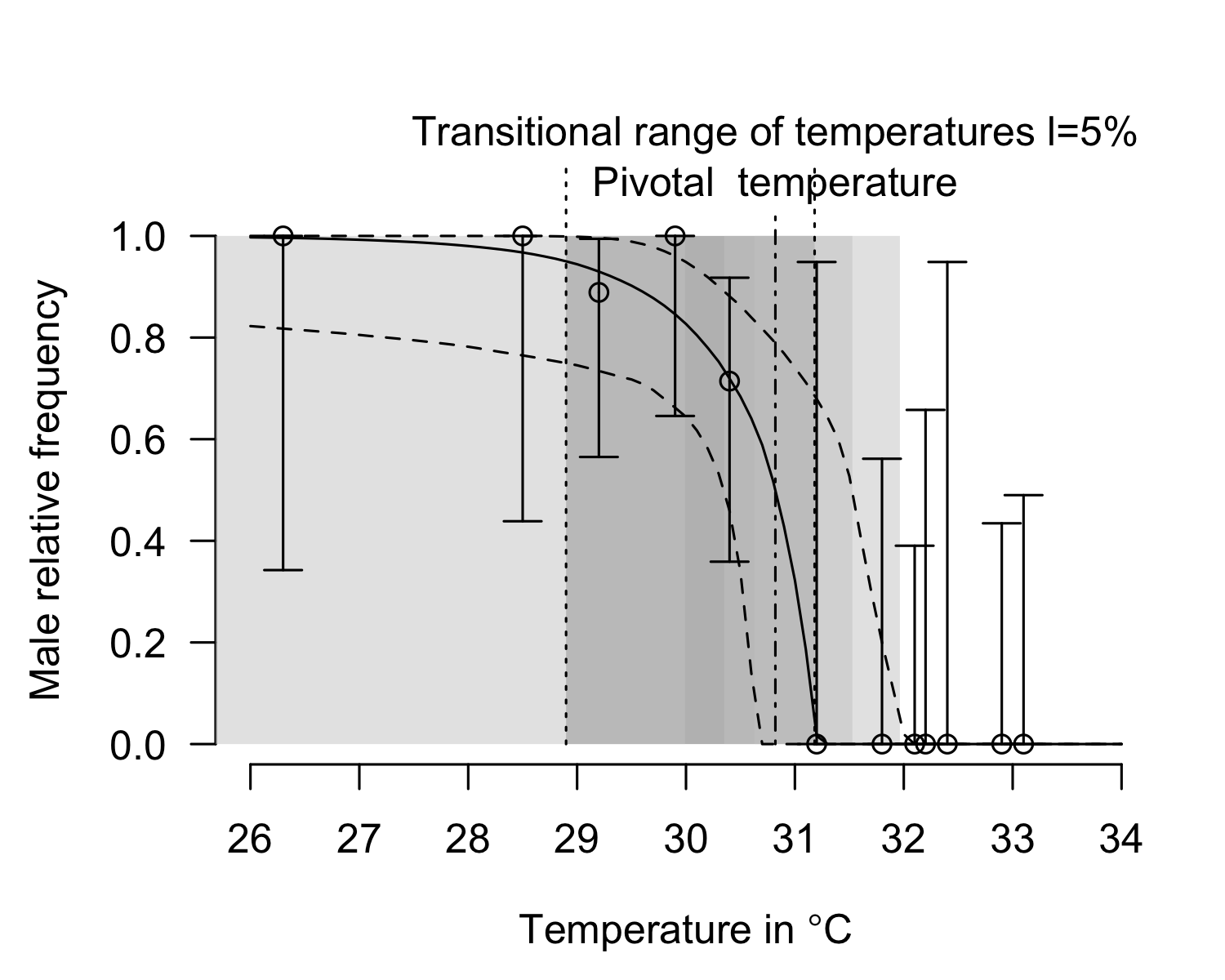


plot(result_mcmc_tsd_LoAtlanticWest_flexit, parameters = "S", xlim=c(-2, 2))


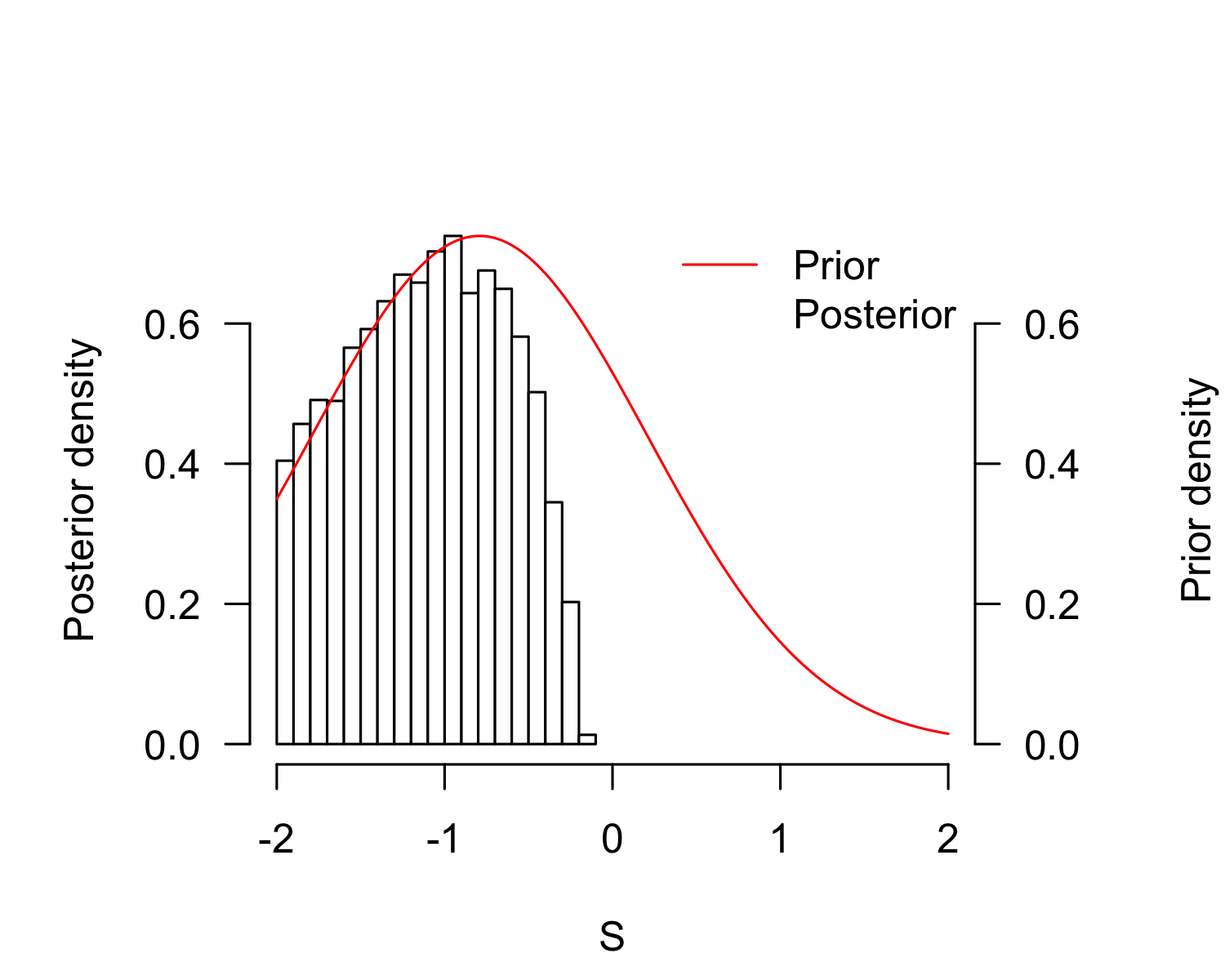


plot(result_mcmc_tsd_LoAtlanticWest_flexit, parameters = "P", xlim=c(25, 35))


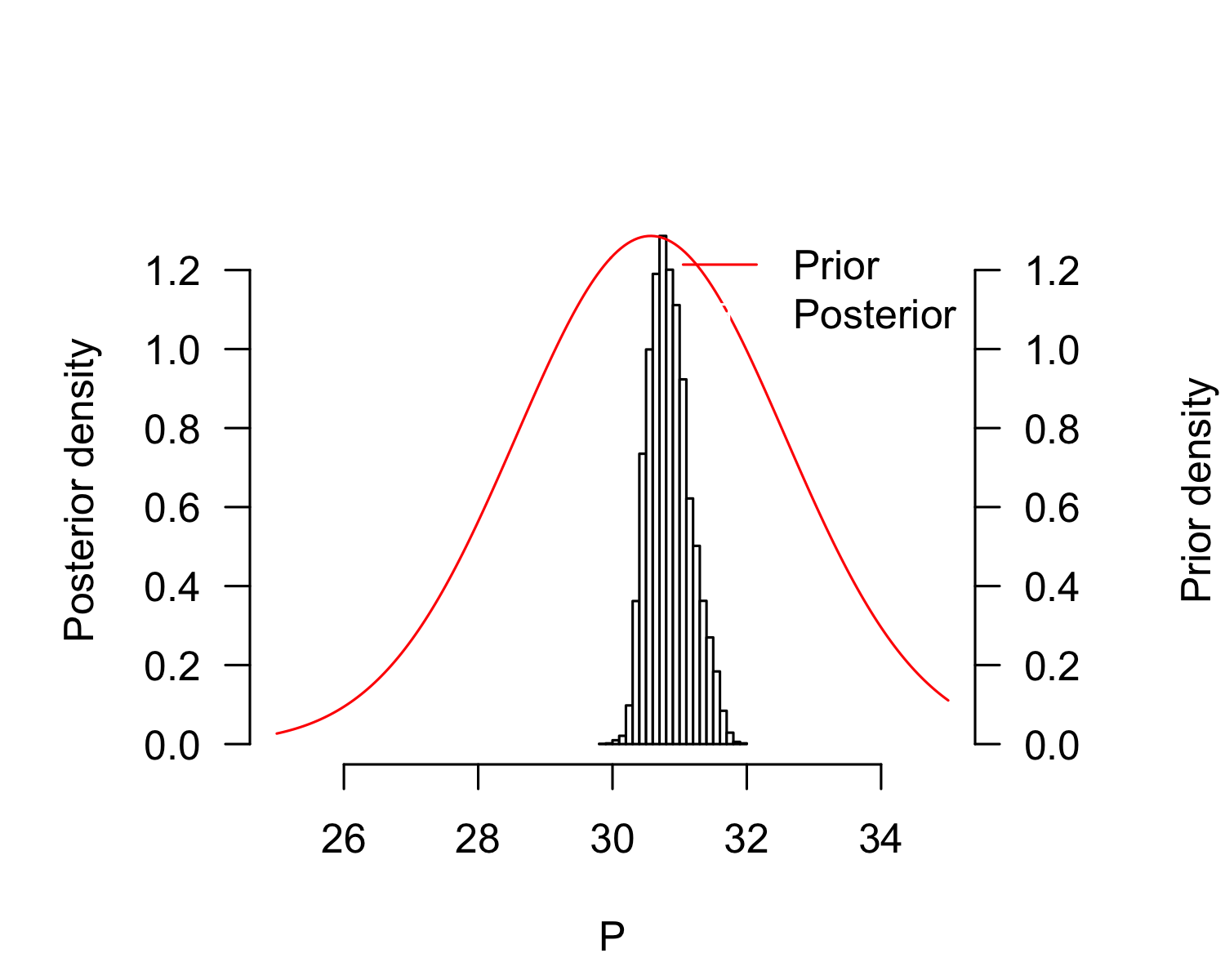


plot(result_mcmc_tsd_LoAtlanticWest_flexit, parameters = "K1", xlim=c(-100, 100))


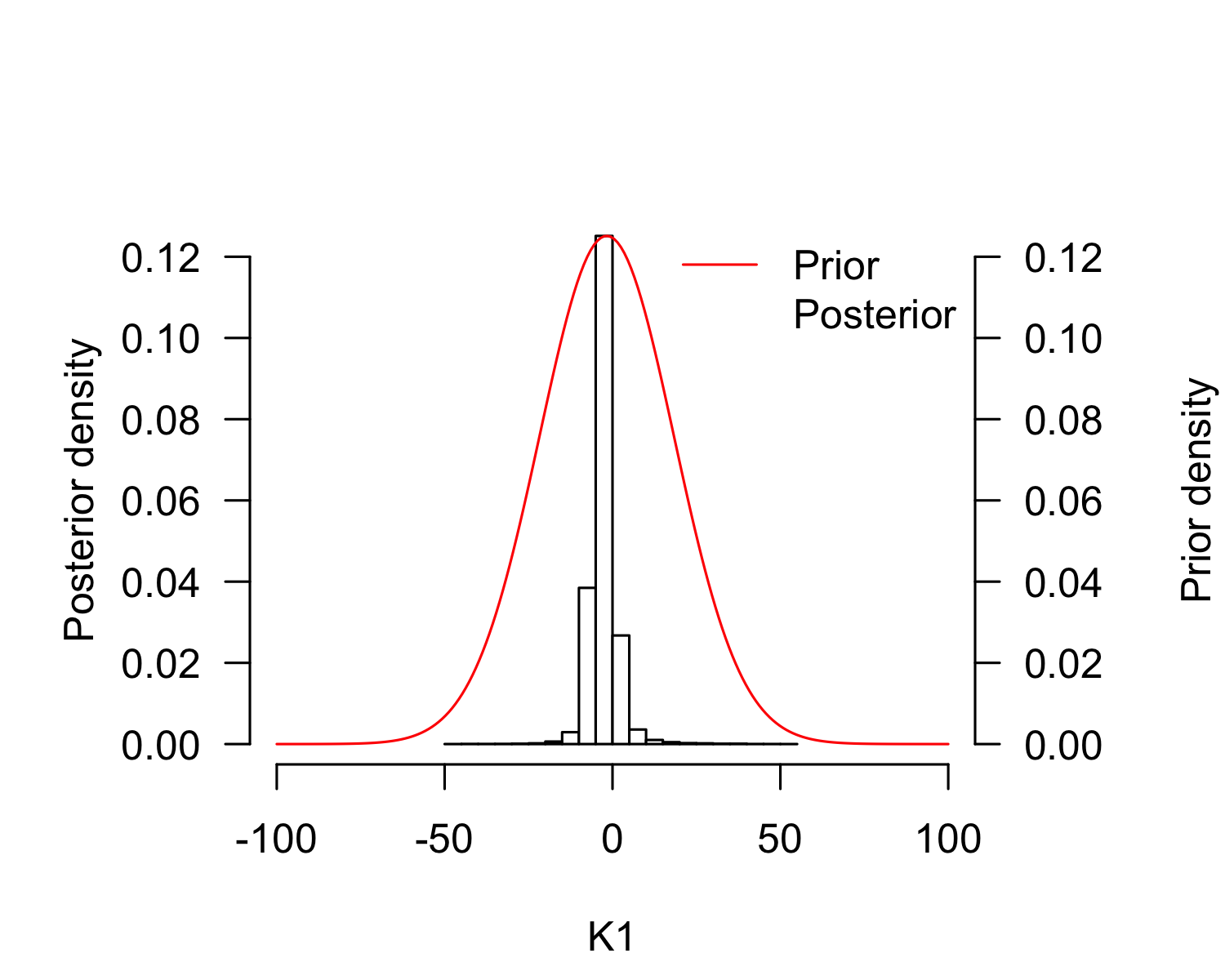


plot(result_mcmc_tsd_LoAtlanticWest_flexit, parameters = "K2", xlim=c(150, 250))


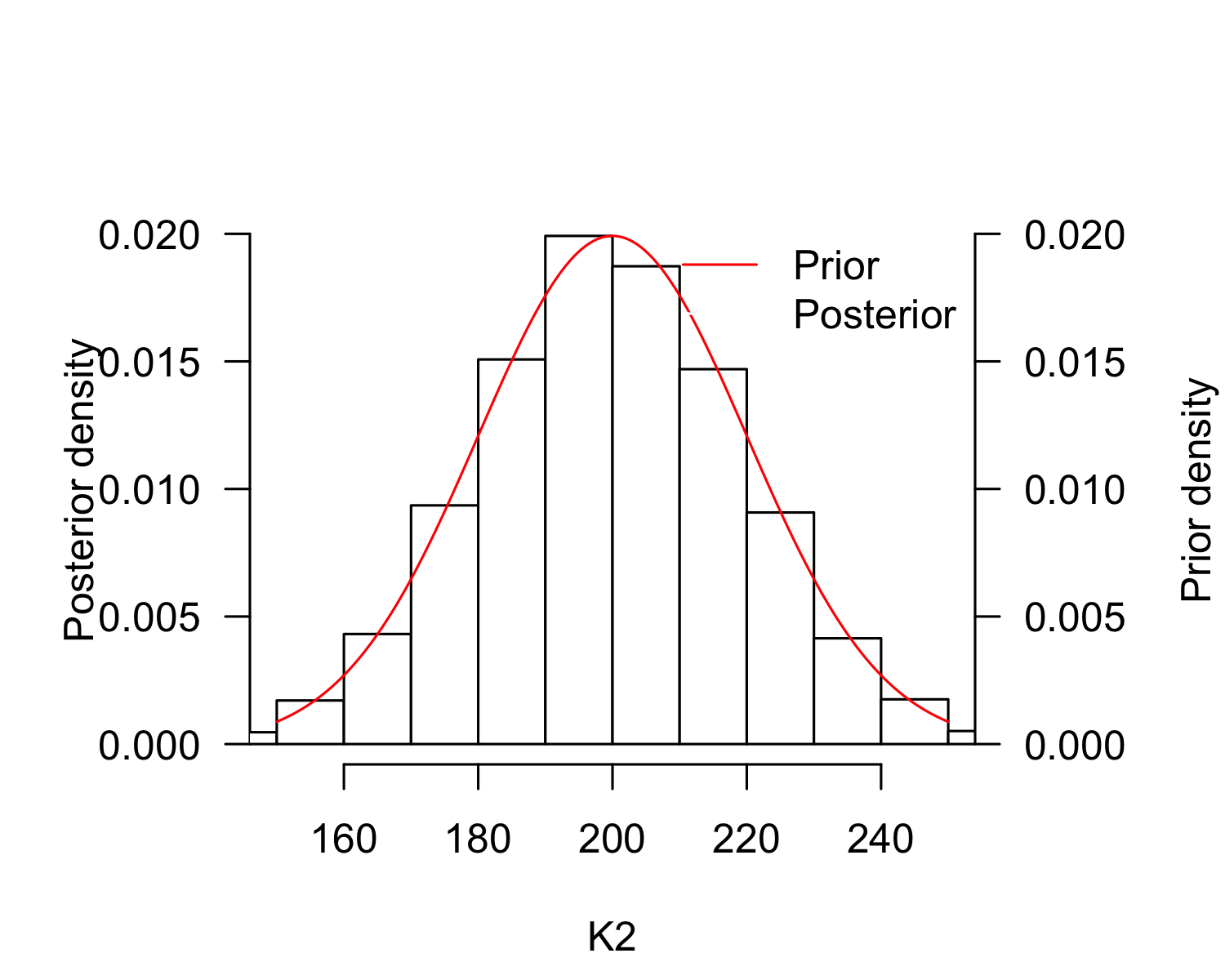


P_TRT_result_mcmc_tsd_LoAtlanticWest_flexit <- P_TRT(resultmcmc = result_mcmc_tsd_LoAtlanticWest_flexit,
 replicate.CI = 100000)
P_TRT_result_mcmc_tsd_LoAtlanticWest_flexit$P_TRT_quantiles

## lower.limit.TRT higher.limit.TRT TRT PT
## 2.5% 4.86480 30.63256 1.022502 30.34801
## 50% 28.88977 31.18099 2.384746 30.82443
## 97.5% 29.98359 31.95719 26.501739 31.51915

## India, Northeast India

tsdL_Lo_IndianNE_India_flexit <- tsdL_Lo_Global_flexit
tsdL_Lo_IndianNE_India_flexit$males <- Lo_IndianNE$Males
tsdL_Lo_IndianNE_India_flexit$females <- Lo_IndianNE$Females
tsdL_Lo_IndianNE_India_flexit$N <- Lo_IndianNE$Males + Lo_IndianNE$Females
tsdL_Lo_IndianNE_India_flexit$temperatures <- Lo_IndianNE$Incubation.temperature

pMCMC_LoIndianNE_India <- tsd_MHmcmc_p(tsdL_Lo_AtlanticWest_Brazil_flexit, accept=TRUE)

result_mcmc_tsd_LoIndianNE_India_flexit <- tsd_MHmcmc(result=tsdL_Lo_IndianNE_India_flexit,
 parametersMCMC=pMCMC_LoIndianNE_India, n.iter=100000,
 n.chains = 1,
 n.adapt = 0, thin=1, trace=FALSE, adaptive = TRUE)

## Density Prior1 Prior2 SDProp Min Max Init
## P dnorm 30.5691666 2 2.0 25.0000 35.0000 30.5691666
## S dnorm -0.7919917 1 0.5 -2.0000 2.0000 -0.7919917
## K1 dnorm -1.7179119 20 0.5 -101.7179 100.0000 -1.7179119
## K2 dnorm 200.0001724 20 0.5 -100.0000 300.0002 200.0001724
## Chain 1
## Best likelihood for:
## P = 29.430156927522
## S = -1.30497169509643
## K1 = 20.5264103398801
## K2 = 216.008888238821

plot(tsdL_Lo_IndianNE_India_flexit, resultmcmc = result_mcmc_tsd_LoIndianNE_India_flexit)


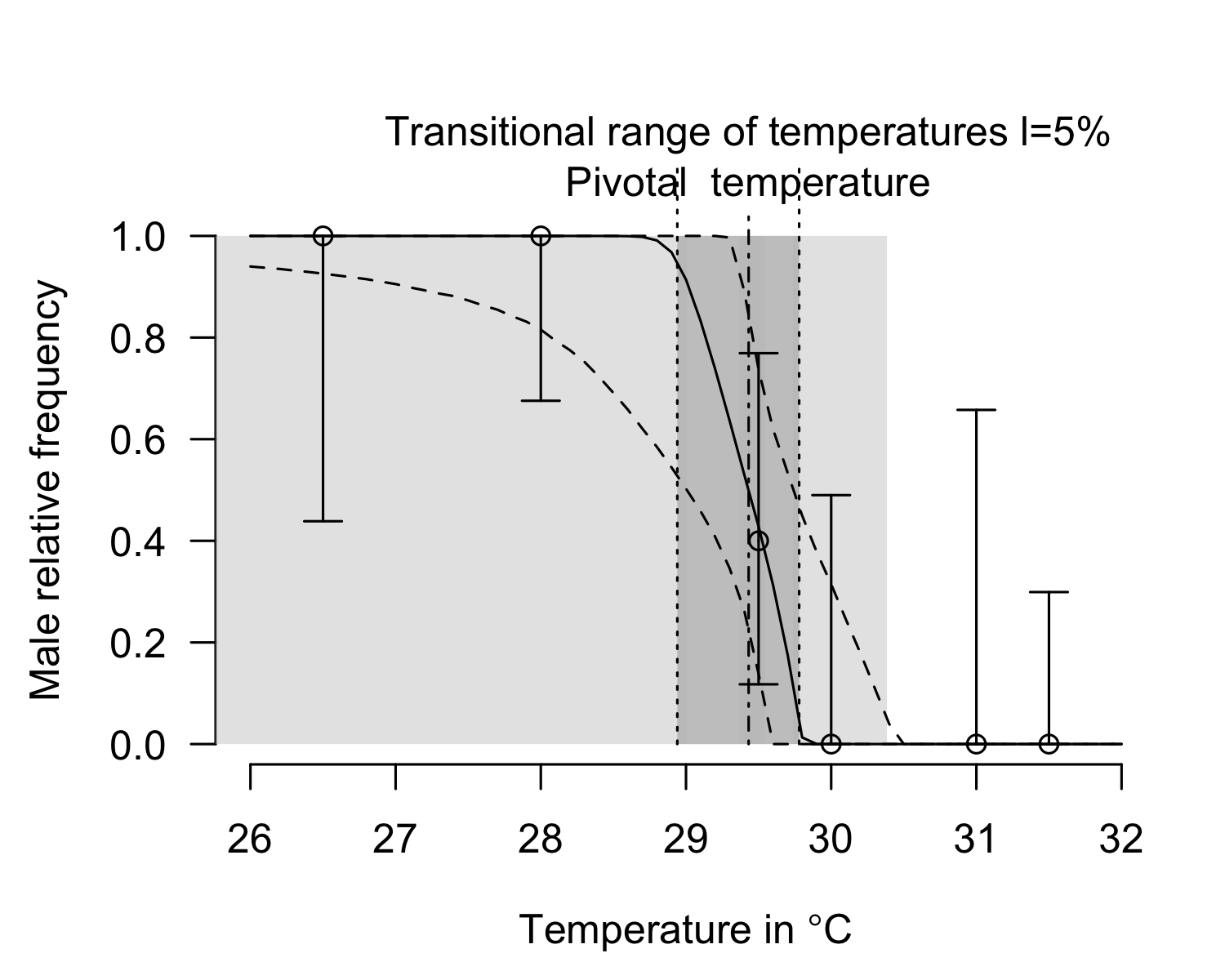


plot(result_mcmc_tsd_LoIndianNE_India_flexit, parameters = "S", xlim=c(-2, 2))


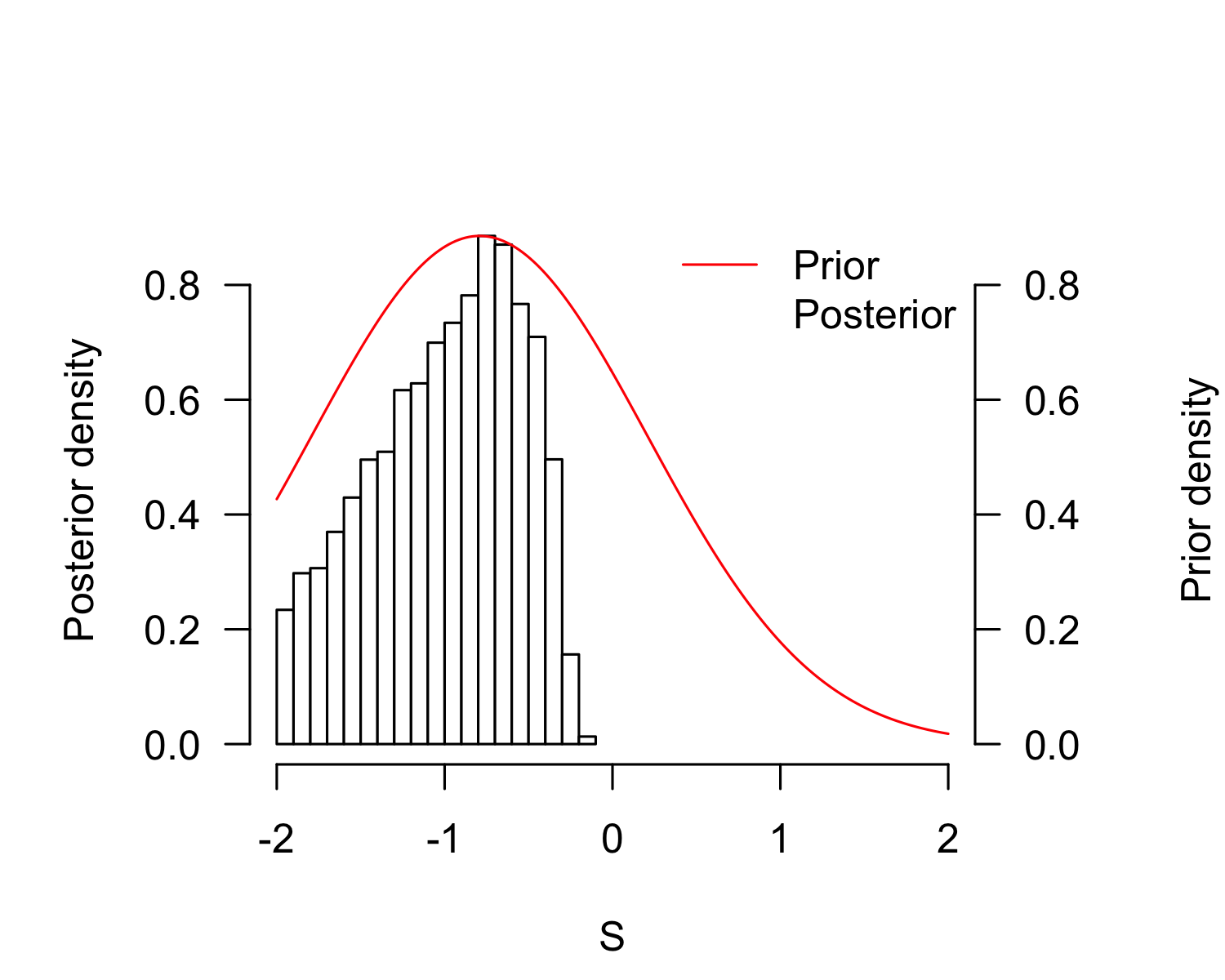


plot(result_mcmc_tsd_LoIndianNE_India_flexit, parameters = "P", xlim=c(25, 35))


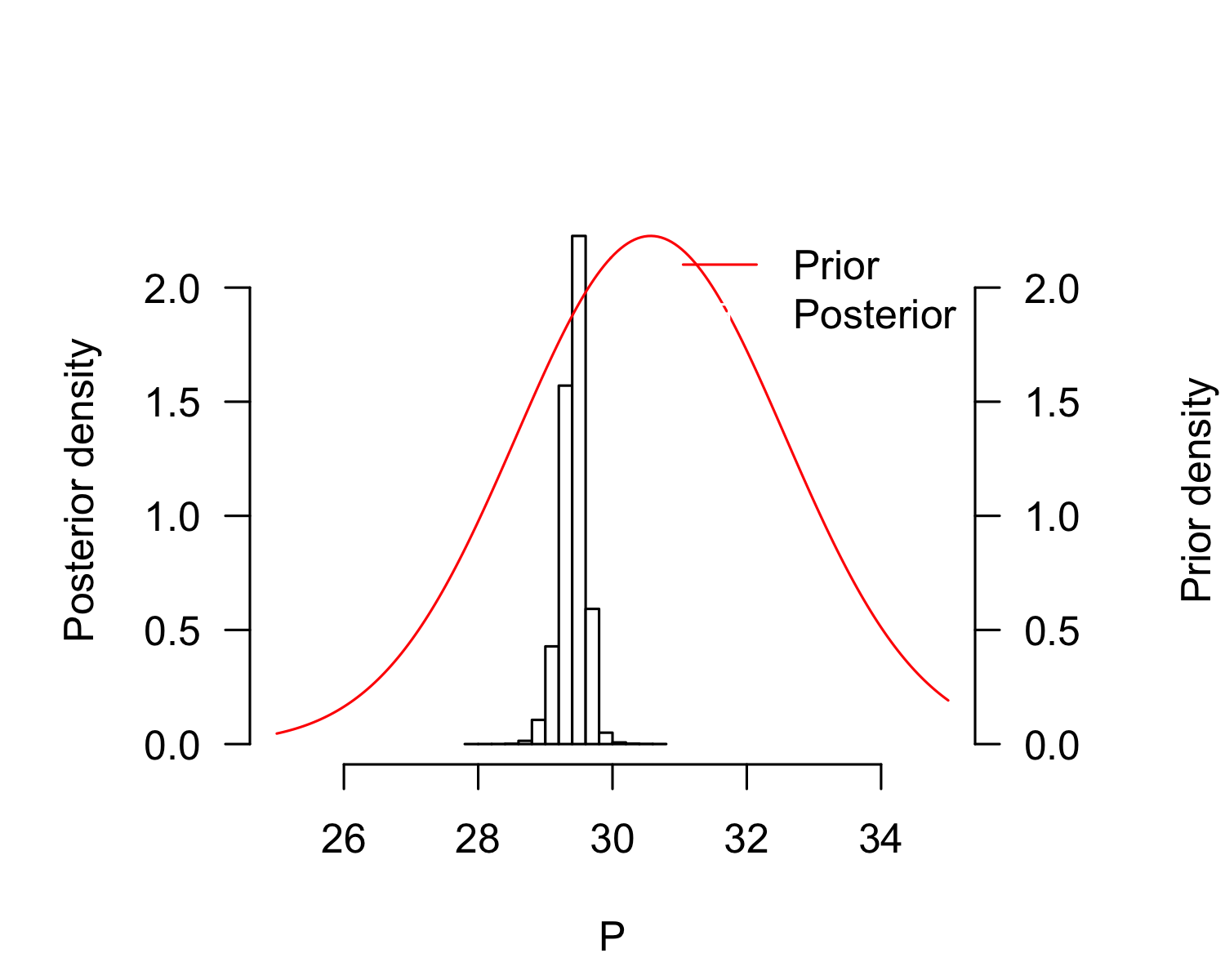


plot(result_mcmc_tsd_LoIndianNE_India_flexit, parameters = "K1", xlim=c(-100, 100))


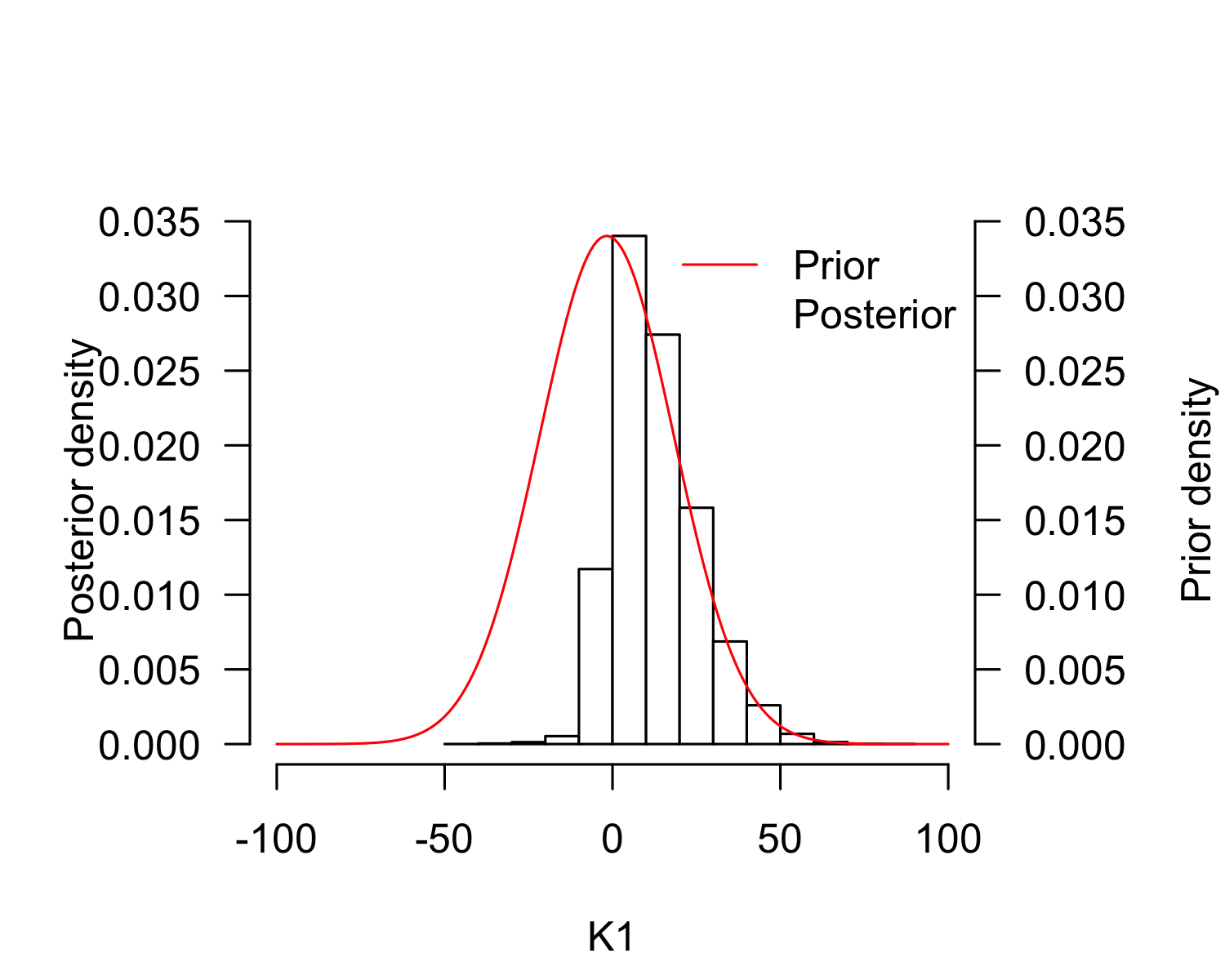


plot(result_mcmc_tsd_LoIndianNE_India_flexit, parameters = "K2", xlim=c(150, 250))


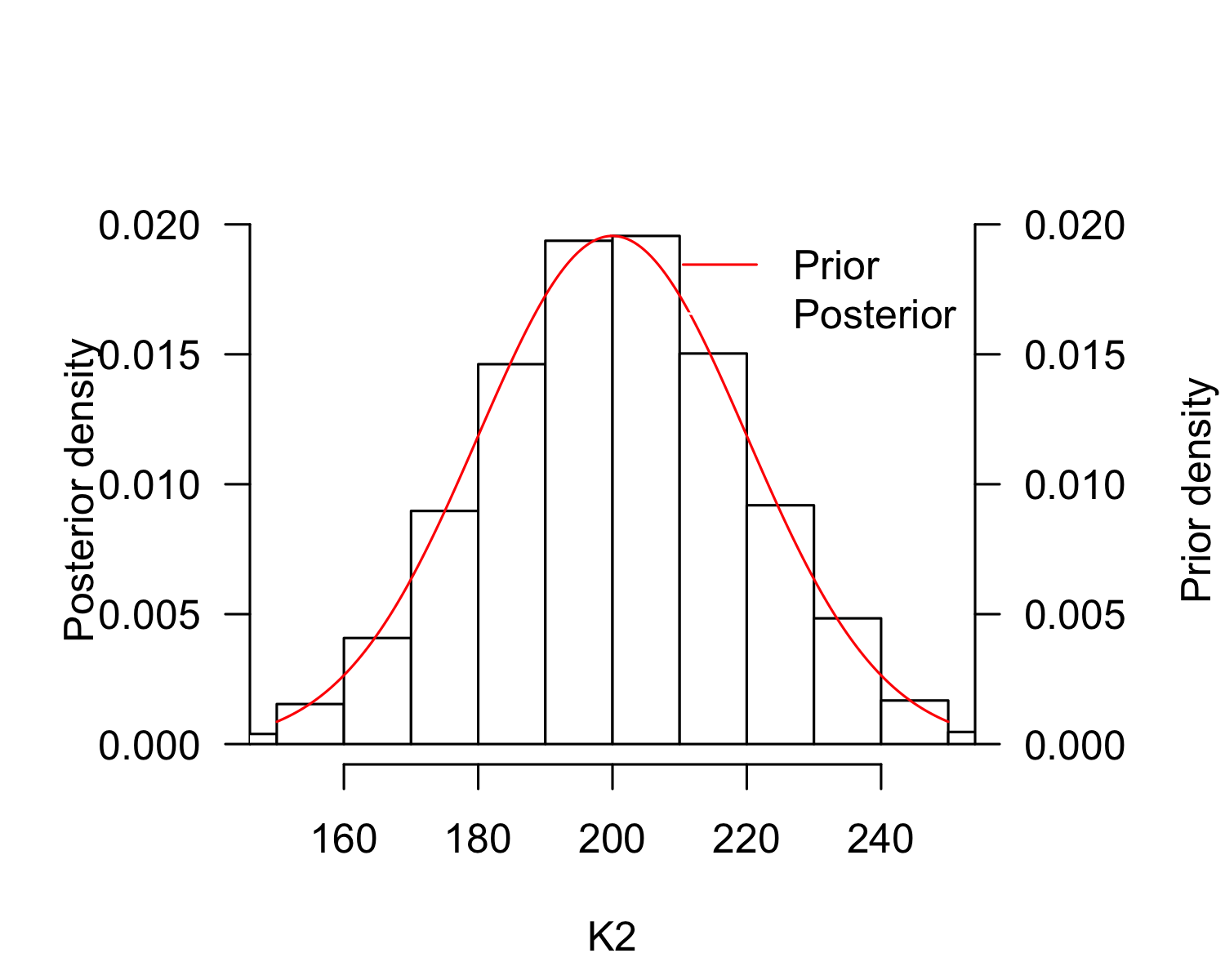


P_TRT_result_mcmc_tsd_LoIndianNE_India_flexit <- P_TRT(resultmcmc = result_mcmc_tsd_LoIndianNE_India_flexit,
 replicate.CI = 100000)
P_TRT_result_mcmc_tsd_LoIndianNE_India_flexit$P_TRT_quantiles

## lower.limit.TRT higher.limit.TRT TRT PT
## 2.5% 25.38478 29.54651 0.3643755 29.00472
## 50% 28.93513 29.77959 0.8716298 29.43097
## 97.5% 29.36709 30.41551 4.4958914 29.74213

# TRT and P relationship for 4 locations from Bayesian analysis with flexit model

Costa Rica (RMU Pacific East), Mexico (RMU Pacific East), and Brazil (Atlantic West) cannot be differentiated.

On the other hand, India (RMU Atlantic West) is different from all others.

par(mar=c(4, 4, 1, 1))
rd <- runif(20000, min=1, max = 100001)
plot(x = P_TRT_result_mcmc_tsd_LoPacificE_Mexico_flexit$P_TRT[rd, "PT"],
 y = P_TRT_result_mcmc_tsd_LoPacificE_Mexico_flexit$P_TRT[rd, "TRT"],
 pch=".", xlim=c(27, 33), ylim=c(0, 8), xlab="Pivotal temperature", ylab="Transitional range of temperatures 5%", bty="n", las=1, col="black")
points(x = P_TRT_result_mcmc_tsd_LoPacificE_CostaRica_flexit$P_TRT[rd, "PT"],
 y = P_TRT_result_mcmc_tsd_LoPacificE_CostaRica_flexit$P_TRT[rd, "TRT"],
 pch=".", col="red")
points(x = P_TRT_result_mcmc_tsd_LoIndianNE_India_flexit$P_TRT[rd, "PT"],
 y = P_TRT_result_mcmc_tsd_LoIndianNE_India_flexit$P_TRT[rd, "TRT"],
 pch=".", col="green")
points(x = P_TRT_result_mcmc_tsd_LoAtlanticWest_flexit$P_TRT[rd, "PT"],
 y = P_TRT_result_mcmc_tsd_LoAtlanticWest_flexit$P_TRT[rd, "TRT"],
 pch=".", col="blue")
points(x = P_TRT_result_mcmc_tsd_LoPacificE_CostaRica_flexit$P_TRT[rd, "PT"],
 y = P_TRT_result_mcmc_tsd_LoPacificE_CostaRica_flexit$P_TRT[rd, "TRT"],
 pch=".", col="red")
points(x = P_TRT_result_mcmc_tsd_LoPacificE_Mexico_flexit$P_TRT[rd, "PT"],
 y = P_TRT_result_mcmc_tsd_LoPacificE_Mexico_flexit$P_TRT[rd, "TRT"],
 pch=".", col="black")
legend("topleft",
 legend=c("Mexico, East Pacific", "Costa Rica, East Pacific",
 "Brazil, West Atlantic", "India, Northeast Indian"),
 pch=rep(19, 4),
 col=c("black", "red", "blue", "green"), cex=0.8)


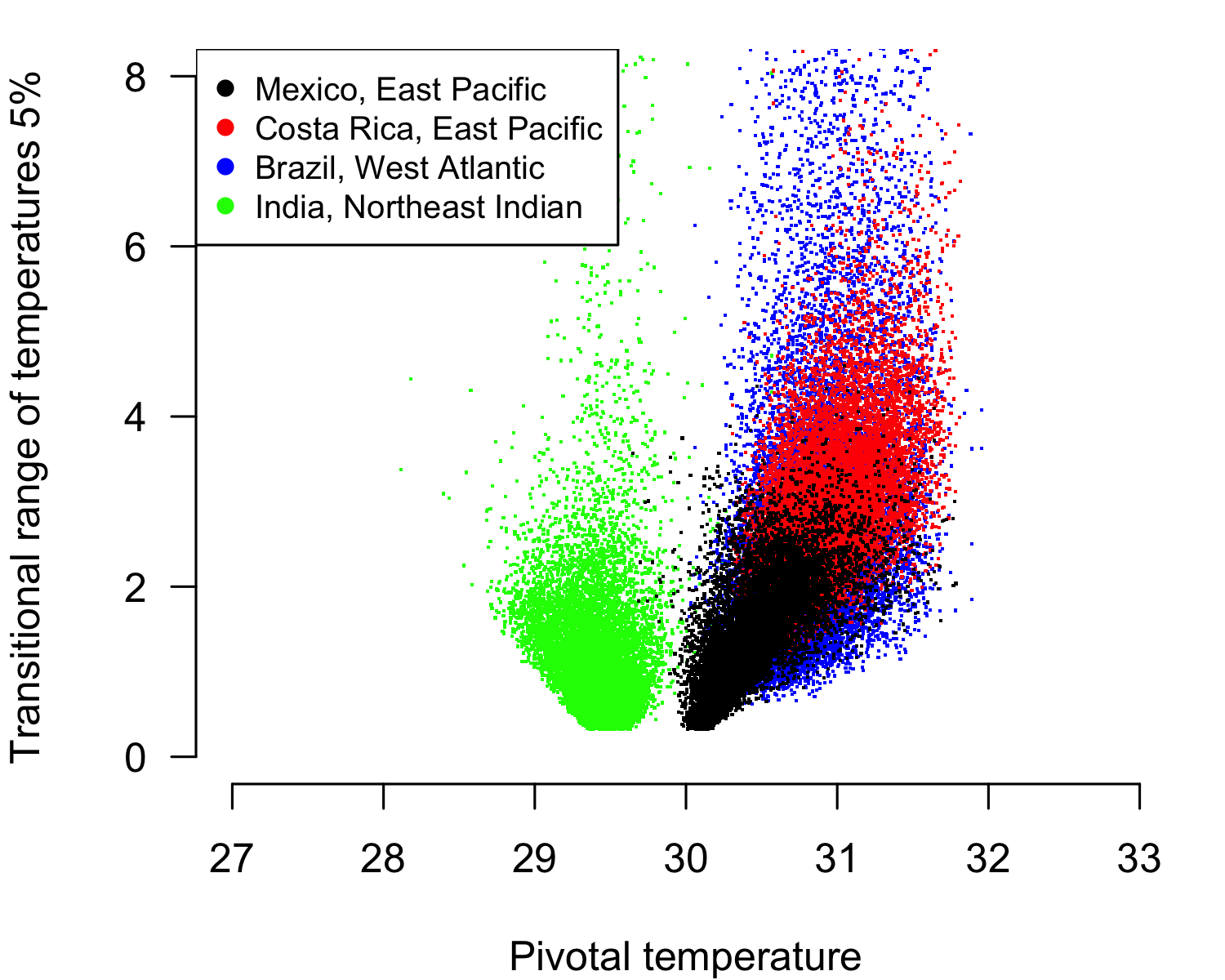


# Prepare the Table 3

result <- tsdL_Lo_IndianNE_logistic
out1 <- data.frame(model="Logistic",
 P=paste(round(result$par["P"], digits = 2), "SE", round(result$SE["P"], digits = 2)),
 S=paste(round(result$par["S"], digits = 2), "SE", round(result$SE["S"], digits = 2)),
 K1="",
 K2="",
 LnL=as.character(round(result$value, digits = 2)),
 Deviance=as.character(round(result$deviance, digits = 2)),
 df=as.character(round(result$df, digits = 0)),
 p.value=as.character(round(result$pvalue, digits = 6)),
 p.Deviance.Null.model=as.character(round(result$NullDeviancePvalue, digits = 2))
)

result <- tsdL_Lo_AtlanticWest_logistic
out1 <- rbind(out1, data.frame(model="Logistic",
 P=paste(round(result$par["P"], digits = 2), "SE", round(result$SE["P"], digits = 2)),
 S=paste(round(result$par["S"], digits = 2), "SE", round(result$SE["S"], digits = 2)),
 K1="",
 K2="",
 LnL=as.character(round(result$value, digits = 2)),
 Deviance=as.character(round(result$deviance, digits = 2)),
 df=as.character(round(result$df, digits = 0)),
 p.value=as.character(round(result$pvalue, digits = 6)),
 p.Deviance.Null.model=as.character(round(result$NullDeviancePvalue, digits = 2))
)
)

result <- tsdL_Lo_PacificE_logistic
out1 <- rbind(out1, data.frame(model="Logistic",
 P=paste(round(result$par["P"], digits = 2), "SE", round(result$SE["P"], digits = 2)),
 S=paste(round(result$par["S"], digits = 2), "SE", round(result$SE["S"], digits = 2)),
 K1="",
 K2="",
 LnL=as.character(round(result$value, digits = 2)),
 Deviance=as.character(round(result$deviance, digits = 2)),
 df=as.character(round(result$df, digits = 0)),
 p.value=as.character(round(result$pvalue, digits = 6)),
 p.Deviance.Null.model=as.character(round(result$NullDeviancePvalue, digits = 2))
)
)

result <- tsdL_Lo_PacificE_CostaRica_logistic
out1 <- rbind(out1, data.frame(model="Logistic",
 P=paste(round(result$par["P"], digits = 2), "SE", round(result$SE["P"], digits = 2)),
 S=paste(round(result$par["S"], digits = 2), "SE", round(result$SE["S"], digits = 2)),
 K1="",
 K2="",
 LnL=as.character(round(result$value, digits = 2)),
 Deviance=as.character(round(result$deviance, digits = 2)),
 df=as.character(round(result$df, digits = 0)),
 p.value=as.character(round(result$pvalue, digits = 6)),
 p.Deviance.Null.model=as.character(round(result$NullDeviancePvalue, digits = 2))
)
)

result <- tsdL_Lo_PacificE_Mexico_logistic
out1 <- rbind(out1, data.frame(model="Logistic",
 P=paste(round(result$par["P"], digits = 2), "SE", round(result$SE["P"], digits = 2)),
 S=paste(round(result$par["S"], digits = 2), "SE", round(result$SE["S"], digits = 2)),
 K1="",
 K2="",
 LnL=as.character(round(result$value, digits = 2)),
 Deviance=as.character(round(result$deviance, digits = 2)),
 df=as.character(round(result$df, digits = 0)),
 p.value=as.character(round(result$pvalue, digits = 6)),
 p.Deviance.Null.model=as.character(round(result$NullDeviancePvalue, digits = 2))
)
)

# write.xlsx(x=out1, file="Table3.xlsx", asTable = FALSE)

save.image("ALLResults.Rdata")
